# Caffeine intake and late dry age-related macular degeneration: tea’s protective role—insights from NHANES and Mendelian randomization

**DOI:** 10.1186/s40942-026-00813-6

**Published:** 2026-02-16

**Authors:** Hongli Yang, Boshi Liu, Yunxi Zhang, Zhanhe Zhang, Huang Tan, Xiaorong Li

**Affiliations:** Tianjin Key Laboratory of Retinal Functions and Diseases, Tianjin Branch of National Clinical Research Center for Ocular Disease, Eye Institute and School of Optometry, Tianjin Medical University Eye Hospital, No. 251, Fukang Road, Tianjin, 300384 China

**Keywords:** Age-related macular degeneration, Geographic atrophy, Caffeine, Tea, NHANES, Mendelian randomization

## Abstract

**Background:**

Although caffeine is widely consumed and has demonstrated neuroprotective effects, its role in age-related macular degeneration (AMD) remains unclear, particularly across disease subtypes and dietary sources such as tea and coffee.

**Methods:**

We analyzed 2005–2008 NHANES data using weighted logistic regression and restricted cubic splines to assess the dose–response relationship between caffeine intake and the prevalence of early and late AMD. Two-sample Mendelian randomization (MR) using GWAS summary statistics was employed to evaluate causal effects of tea and coffee consumption on AMD subtypes. Furthermore, a two-step MR approach was utilized to identify potential immune-mediated pathways.

**Results:**

NHANES data showed that caffeine intake was inversely associated with late AMD (fully adjusted OR = 0.65, 95% CI: 0.44–0.96). Dose–response modeling revealed an L-shaped nonlinear relationship (P for nonlinear = 0.046), indicating that the protective effect of caffeine plateaued once daily intake exceeded approximately 110–150 mg. MR analysis further supported a causal protective association between tea consumption and dry AMD, including geographic atrophy (OR = 0.44, 95% CI: 0.20–0.97), which may be partially attributable to immunological mechanisms, specifically the downregulation of secretory regulatory T cells (% of CD4 + Tregs) and CD45RA- CD4 + T cell (% of CD4 + T cell). In contrast, coffee consumption showed no significant effect.

**Conclusions:**

Tea, a specific source of caffeine typically corresponding to moderate intake levels, may confer protection against dry AMD, including geographic atrophy, potentially through modulation of immune cell profiles. These findings suggest a potential preventive strategy and warrant further clinical investigation.

**Graphical abstract:**

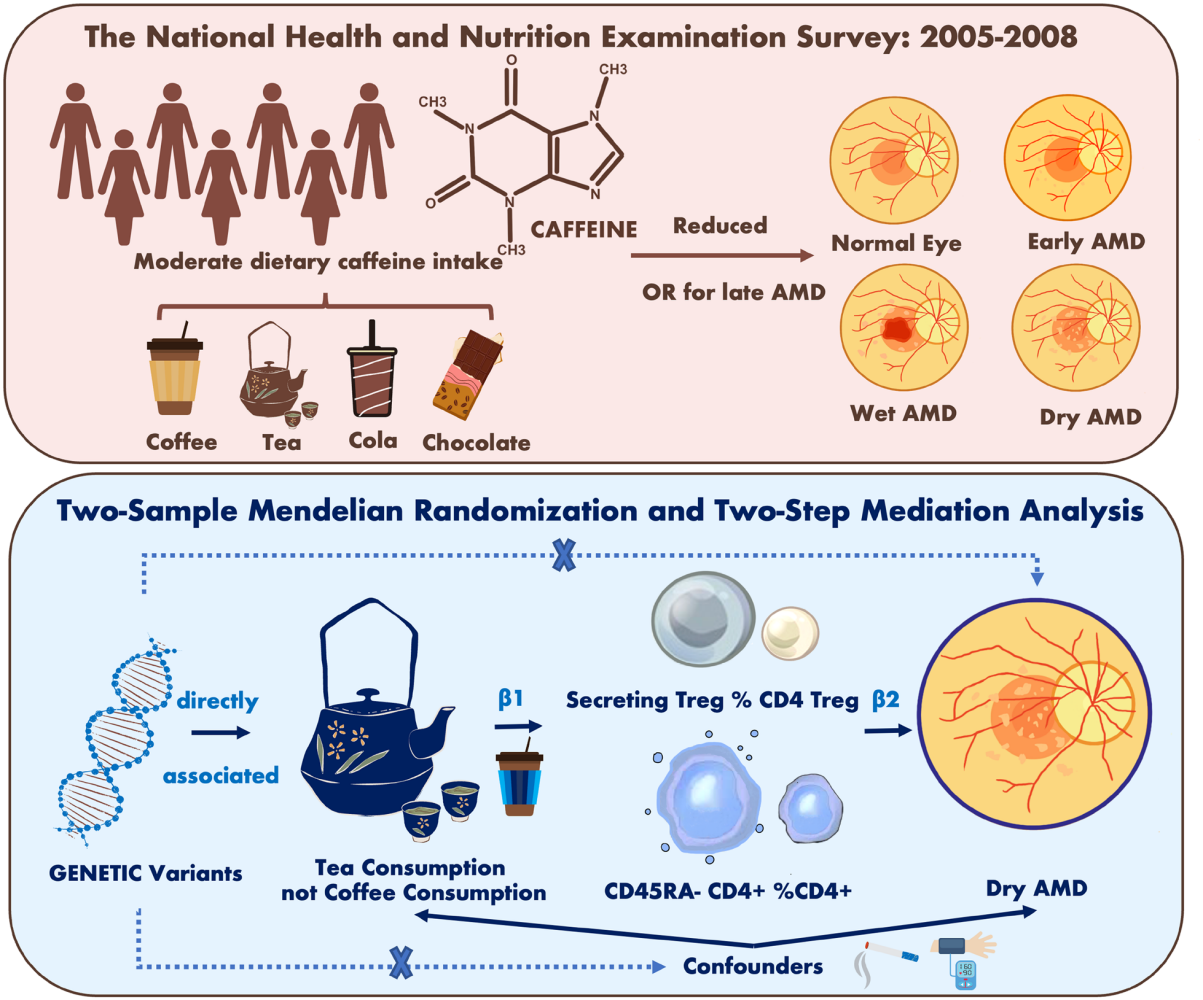

**Supplementary Information:**

The online version contains supplementary material available at 10.1186/s40942-026-00813-6.

## Background

Age-related macular degeneration (AMD) is a leading cause of irreversible vision loss in older adults worldwide [1]. With the rapid aging of the population, its late-stage manifestations—neovascular (wet) AMD and geographic atrophy (GA, late-stage dry AMD) [2]—pose a substantial and growing economic and societal burden [3–5]. Although anti–vascular endothelial growth factor (anti-VEGF) therapy has revolutionized the prognosis of wet AMD in many patients, treatment responses vary considerably across individuals. More critically, effective therapeutic options for late-stage dry AMD remain largely unavailable in many regions [6], highlighting the urgent need for preventive strategies.

Chronic inflammation and immune dysregulation are widely recognized as central mechanisms in AMD pathogenesis [7, 8]. Accumulating evidence indicates that pro-inflammatory T-cell subsets contribute to retinal degeneration and disease progression [9–11]. Caffeine, a widely consumed bioactive compound primarily derived from tea and coffee, has been reported to exert neuroprotective effects through anti-inflammatory and neuroimmunomodulatory pathways [12–19]. However, conventional observational studies examining the association between caffeine intake and AMD risk have yielded inconsistent findings. For instance, the Beaver Dam Eye Study reported no significant association between caffeine intake and early AMD [20], while two other studies suggesting a protective effect are often confounded by complex dietary patterns or inadequate adjustment for covariates [21, 22].

In recent years, Mendelian randomization (MR) has emerged as a powerful approach for strengthening causal inference in nutritional epidemiology and age-related eye diseases. Emerging MR studies have not only supported a protective role of water intake against age-related cataracts and diabetic retinopathy [23], but have also highlighted the contribution of gut microbiota—such as Lactobacillus—to visual and neurological health through the eye–brain–gut axis [24]. Importantly, MR investigations into caffeine and AMD have revealed a potential “source paradox” that was not apparent in traditional epidemiological studies. Specifically, a two-sample MR analysis of genetically predicted plasma caffeine levels demonstrated protective effects against cataracts and glaucoma but found no significant association with AMD [25]. In contrast, another MR study identified a causal association between instant coffee consumption and an increased risk of dry AMD [26]. This discrepancy—characterized by an overall lack of effect from plasma caffeine but potential harm from specific sources—suggests that caffeine cannot be evaluated as an isolated nutrient. Instead, its impact must be contextualized within its specific dietary carriers, such as tea and coffee [27, 28].

Against this background, we adopted a multidimensional analytical strategy to address these knowledge gaps. First, we utilized data from the National Health and Nutrition Examination Survey (NHANES) to investigate the association between caffeine intake and AMD stages. Second, to overcome the inherent limitations of observational analyses and to disentangle the proposed source-specific effects, we conducted two-sample MR analyses to evaluate the potential causal roles of tea and coffee—the two principal caffeine sources—on AMD subtypes. Finally, we explored the contribution of immune cell traits as potential mediators, aiming to elucidate biologically plausible pathways underlying the observed associations and to inform future prevention strategies.

## Methods

### Overall study design

This study employed two complementary analytical approaches. First, a cross-sectional analysis was conducted using data from the 2005–2008 National Health and Nutrition Examination Survey (NHANES) to examine the association and dose–response relationship between daily caffeine intake and different stages of age-related macular degeneration (AMD). Second, a bidirectional two-sample Mendelian randomization (MR) analysis was applied to evaluate the potential causal effects of genetically predicted coffee and tea consumption on AMD subtypes, including early AMD, dry AMD (including geographic atrophy [GA]), and wet AMD. In addition, a two-step MR approach was used to investigate immune cell subsets as potential mediators of these associations. Each analytical strategy has its strengths and limitations: the cross-sectional analysis provides population-level epidemiological evidence, whereas the MR analysis strengthens causal inference by minimizing confounding and reverse causation through the use of genetic instruments.

### Cross-sectional study

#### Study design and participant recruitment

In the cross-sectional analysis section, we analyzed data from two consecutive cycles of the NHANES (2005–2006 and 2007–2008), as these represented the cycles containing both comprehensive digital fundus photography and detailed dietary caffeine intake assessments [29]. NHANES employs a complex, stratified, multistage probability sampling design to obtain a nationally representative sample of the noninstitutionalized U.S. civilian population. The NHANES study protocol was approved by the National Center for Health Statistics Ethics Review Board and conducted in accordance with the Declaration of Helsinki. Written informed consent was obtained from all participants.

**Fig. 1:**
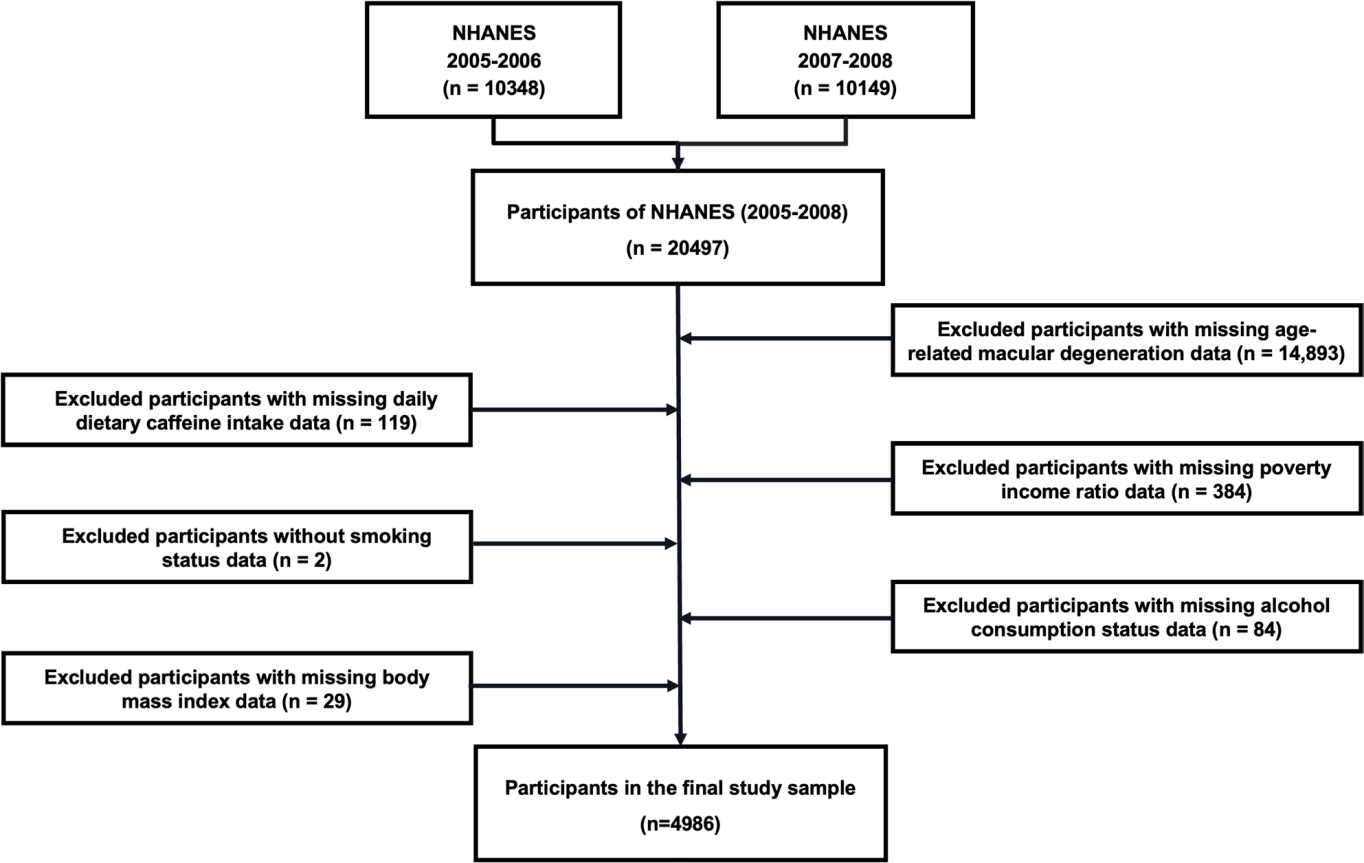
Flow chart of the study design

Participants were eligible for inclusion if they met the following criteria: (1) age ≥ 40 years; (2) completion of at least one valid 24-hour dietary recall interview; and (3) availability of gradable fundus photographs for AMD assessment in at least one eye. A total of 5,485 participants met these criteria. Participants with missing data on key covariates—including poverty income ratio, smoking status, alcohol use, or body mass index (*n* = 499)—were subsequently excluded. The final analytical sample consisted of 4,986 participants. The participant selection process is summarized in Fig. 1. All NHANES data are publicly available at https://www.cdc.gov/nchs/nhanes/ (accessed November 30, 2024).

#### Assessment of dietary caffeine consumption

The primary exposure variable was daily dietary caffeine intake (0.1 g/day). Data on daily dietary caffeine intake (mg/day) were obtained from the “Dietary Interview - Total Nutrient Intakes, First Day” and “Dietary Interview - Total Nutrient Intakes, Second Day” files. In NHANES, dietary intake is assessed using two 24-hour dietary recall interviews: the first conducted in-person at the Mobile Examination Center (MEC) and the second via telephone 3–10 days later. All reported caffeinated foods and beverages—including coffee, tea, energy drinks, chocolate, and regular, low-calorie, or diet sodas—were entered into the Food and Nutrient Database for Dietary Studies (FNDDS) (http://www.ars.usda.gov/ba/bhnrc/fsrg, accessed November 30, 2024) to calculate daily caffeine intake [30]. For participants who completed both dietary recalls, the mean intake from the two days was calculated to provide a more robust estimate of habitual consumption. For those with only the first recall, the initial 24-hour intake value was used.

#### Retinal photography and definition of AMD

The primary outcome was the presence of early or late age-related macular degeneration, assessed using fundus photography. In NHANES, 45-degree nonmydriatic digital retinal images were captured using a Canon CR6-45NM camera in participants aged 40 years and older. Images were assessed by two or more experienced evaluators at the University of Wisconsin. Early AMD was defined by the presence of drusen and/or pigmentary abnormalities, while late AMD was defined by the presence of exudative changes and/or geographic atrophy. When valid images were available for both eyes, the worse eye determined the AMD grade.

#### Study covariates

Potential confounders included demographic characteristics, lifestyle factors, systemic disease complications, and ocular conditions. Demographic variables—age (categorized as < 45, 45–64, or ≥ 65 years), sex, race/ethnicity (non-Hispanic White, non-Hispanic Black, Mexican American, or other), education level (< high school, high school diploma/high school graduate/General Educational Development (GED) or equivalent, > high school), and poverty income ratio (PIR)—were obtained from the NHANES Demographic Variables & Sample Weights module. PIR was calculated by dividing household income by the poverty threshold adjusted for family size; PIR < 1.0 indicated income below the poverty level.

Health-related lifestyle factors included smoking status, alcohol consumption, body mass index (BMI), and total daily dietary calorie intake. Smoking status was based on responses to the “Smoking – Cigarette Use” questionnaire and classified as: current smokers (≥ 100 cigarettes in lifetime and currently smoking), former smokers (≥ 100 cigarettes in lifetime but no longer smoking), and never smokers (< 100 cigarettes in lifetime). Alcohol consumption was categorized as nondrinking (≤ 12 times per year), drinking 1–5 times per month, or > 5 times per month over the past year. BMI (kg/m²) was calculated from measured height and weight, obtained from the Body Measures module. Total daily dietary calorie intake (kcal) was averaged from the first and second 24-hour dietary recalls; for participants with only the first recall available, that recall was used.

Systemic disease complications considered in the analysis included diabetes, hypertension, and a history of cardiovascular disease (CVD). Diabetes was defined by any one of the following: a prior physician diagnosis; current use of insulin or diabetes medication; glycated hemoglobin (HbA1c) > 6.5%; or fasting glucose > 126 mg/dL. Hypertension was defined as a prior physician diagnosis, an average systolic blood pressure ≥ 140 mmHg, or an average diastolic blood pressure ≥ 90 mmHg. History of CVD was defined as a self-reported physician diagnosis of congestive heart failure, coronary artery disease, angina, heart attack, or stroke. Ocular history included self-reported cataract surgery, or a physician-diagnosed glaucoma based on questionnaire data.

### Mendelian randomization

#### Study design

Mendelian randomization (MR) relies on three core instrumental variable (IV) assumptions: (1) IVs must be significantly associated with exposure factors (coffee and tea consumption); (2) IVs should be uncorrelated with confounders; and (3) IVs should influence AMD stages solely through coffee and tea consumption, not other pathways.

We conducted a bidirectional two-sample MR analysis to explore causal relationships between genetically predicted coffee and tea consumption—the primary caffeine sources [31]—and the risk of early, dry (including geographic atrophy), and wet AMD. Given the extensive evidence that overactive immune responses contribute to AMD development [32], we further performed a two-step MR analysis to investigate potential immune-mediated pathways. Specifically, we incorporated 17 out of 731 immune cell types previously identified by Wei et al. as significantly associated with AMD through univariate, bidirectional, and multivariable MR analyses [33]. The study was conducted in accordance with the STROBE-MR reporting guidelines (see Supplementary Materials for checklist).

#### Data source

Summary-level GWAS data used in this study were obtained from publicly available datasets based on European cohorts, with all participants of European descent. Data on coffee and tea consumption—treated as binary variables based on the question “Did you drink coffee/tea yesterday?“—were sourced from the UK Biobank via the MRC IEU GWAS database (https://gwas.mrcieu.ac.uk/, accessed November 30, 2024) [34]. Early AMD outcomes were derived from summary statistics provided by the International AMD Genomics Consortium (IAMDGC), which included data from 11 cohorts (*n* = 105,248; 14,034 cases and 91,214 controls) [35]. Data for dry and wet AMD were obtained from the FinnGen research project (https://r11.finngen.fi/, accessed November 30, 2024), which categorizes age-related central vision loss due to retinal degeneration into wet AMD and dry AMD, including geographic atrophy (GA).

The mediator—immune cell type—was derived from a high-density genotyping array consisting of approximately 22 million single-nucleotide polymorphisms (SNPs), based on Sardinian sequences, as reported in the GWAS Catalog [36]. In MR analysis, sample overlap between exposure and outcome datasets can introduce bias and increase the risk of type I error. In our study, some early AMD outcome data from the UK Biobank partially overlapped with the exposure data on caffeinated beverage consumption. To account for this, we estimated the degree of bias and the probability of type I error using a publicly available tool (https://sb452.shinyapps.io/overlap/, accessed November 30, 2024) [37]. The FinnGen study, which aggregates data from nine Finnish biobank GWASs, and the immune cell type GWAS, conducted in participants from Sardinia (Mediterranean Italy), are genetically and geographically distinct from the UK Biobank cohort. Therefore, sample overlap across other exposures, mediators, and outcomes is likely minimal, reducing the risk of bias in our MR analyses. Detailed data sources are listed in Table S1. All GWAS summary-level data were derived from publicly available resources with prior ethical approval and written informed consent from all participants.

#### Instrumental variable selection and data harmonization

To meet the three core assumptions of MR, we applied the following criteria for selecting SNPs as instrumental variables (IVs): (1) Relevance: SNPs were initially selected based on genome-wide significance (*P* < 5 × 10⁻⁸). Due to the limited number of SNPs identified for coffee and tea consumption, this threshold was relaxed to *P* < 5 × 10⁻⁶ to ensure an adequate number of instruments for MR analyses [38, 39]. For the 17 immune cell types previously reported by Wei et al., we used a stricter threshold of *P* < 1 × 10⁻⁸, consistent with their methodology; if no SNPs met this criterion, we applied a relaxed threshold of *P* < 5 × 10⁻⁶. (2) Independence: Linkage disequilibrium (LD) between SNPs was addressed using a 10,000 kb window and an r² threshold of < 0.001 to ensure independence. (3) Instrument strength: The strength of each IV was assessed using the F-statistic, calculated as F = R²(n − k − 1) / [k(1 − R²)], where R² = 2 × MAF × (1 − MAF) × β²; MAF is the minor allele frequency, n is the sample size, and k is the number of IVs. All selected SNPs had F-statistics > 10, indicating sufficient instrument strength and minimizing the risk of weak instrument bias. (4) Harmonization and confounder exclusion: SNP effect alleles for the outcome were harmonized with those for the exposure based on allele letters and frequencies, and palindromic SNPs were excluded to avoid ambiguity. To ensure selected SNPs were not associated with potential confounders, we used the LDtrait Tool (https://ldlink.nih.gov/?tab=ldtrait, accessed November 30, 2024), with an r² threshold of 0.8.

#### Mediation analysis

We performed a two-step mediation MR analysis based on a three-step framework. First, the total causal effect of the exposure on the outcome was estimated (β₀). Second, the causal effect of the exposure on the mediator was assessed (β₁). Third, the causal effect of the mediator on the outcome was estimated (β₂). Evidence of mediation was considered present when all three associations were statistically significant [40]. The mediation effect was calculated using the two-step MR formula: Mediation effect = β₁ × β₂, and the proportion of the total effect mediated (mediation ratio) was calculated as: Mediation ratio = (β₁ × β₂ / β₀) × 100% [41].

#### Sensitivity analysis

Sensitivity analysis is crucial to ensure the reliability of MR results. Heterogeneity among instrumental variables was assessed using Cochran’s Q statistic; with *P* > 0.05 indicating low heterogeneity. In the presence of heterogeneity, a random-effects inverse variance-weighted (IVW) model was applied. Horizontal pleiotropy was evaluated using the intercept from MR-Egger regression. We assessed the validity of the no measurement error (NOME) assumption in MR-Egger analyses using the I²_GX_ statistic, which quantifies the impact of uncertainty in SNP–exposure associations on causal estimates. Additionally, leave-one-out (LOO) analysis was performed to identify and account for influential outliers. Funnel plots were generated to visually inspect the presence of directional pleiotropy. Detailed sensitivity analysis results—including the number of SNPs, F-statistic ranges, Cochran’s Q statistics, MR-Egger intercepts, I²GX values, LOO plots, and funnel plots—are provided in the Supplementary Materials.

### Statistical analysis

All statistical analyses were performed using R software (version 4.4.1). To maximize sample representativeness and minimize selection bias arising from incomplete second-day dietary recalls, analyses primarily applied the NHANES-recommended Day 1 dietary sample weight (WTDRD1), adjusted for the inclusion of two survey cycles. Although some participants completed two dietary recalls and their mean intake was calculated, exclusive use of the two-day dietary weight (WTDR2D) would have restricted analyses to participants completing both interviews, resulting in substantial loss of eligible AMD cases—particularly late AMD cases, which were rare. The weighting strategy adopted here is consistent with previous NHANES-based studies conducted under similar sample-size constraints [42, 43]. Participant characteristics were summarized using the tbl_svysummary function from the gtsummary package (version 2.0.3). Continuous variables were presented as weighted means ± standard error (SE), while categorical variables as unweighted counts with weighted percentages. Group comparisons used survey-weighted Pearson’s χ² tests (Rao–Scott adjustment) for categorical variables and the survey-weighted Kruskal–Wallis test for continuous variables.

Logistic regression models were fitted using svyglm function from the survey package (version 4.4.1) to evaluate the association between caffeine intake (per 0.1 g/day) and AMD stages. The crude model was unadjusted. Model 1 adjusted for demographic variables—age, sex, race, and poverty level—the latter included to account for socioeconomic effects on caffeine intake. Model 2 additionally adjusted for health-related lifestyle factors, including smoking status, alcohol consumption, body mass index, and total daily dietary calorie intake. Model 3 further included additional adjustments for clinical variables: history of CVD, hypertension, cataract surgery, and glaucoma. We used the Glm function from the rms package (version 6.8.2) to perform a weighted restricted cubic spline analysis, assessing the potential nonlinear relationship between daily dietary caffeine intake and the odds ratio of late AMD. Given the limited number of late AMD cases, three knots—the minimum required to model nonlinearity—were specified to reduce the risk of overfitting. The suggested saturation range was identified using the maximum curvature method.

Mendelian randomization was performed using the TwoSampleMR package (version 0.6.8). The inverse-variance weighted (IVW) method with random effects was the primary analysis. Sensitivity analyses included MR-Egger, weighted median, weighted mode, and simple mode. Statistical significance was defined as IVW *P* < 0.05 with concordant effect directions across all methods. Causal inference required significant forward MR and null reverse MR results. A two-tailed P value < 0.05 was considered statistically significant in this study.

## Results

### Cross-sectional study

#### Participant demographics and clinical profiles by AMD stages

A total of 4,986 participants aged 40 years or older were included in this study. Participants were categorized into three groups according to their AMD stages: no AMD, early AMD, and late AMD. Table 1 summarizes the demographic characteristics, health-related lifestyle factors, systemic disease complications, ocular conditions, and daily dietary caffeine intake for each group. Among the participants, 384 individuals (6.58%) had AMD, including 337 with early-stage AMD (5.72%) and 47 in the late stage (0.86%). Consistent with established risk profiles, participants with AMD were generally older, more likely to be non-Hispanic white, and more frequently past or current smokers. They also had significantly higher prevalence of cardiovascular disease, hypertension, a history of cataract surgery, and glaucoma. Additionally, their total daily calorie intake was significantly lower (all *P* < 0.05). As shown in Table S2, participants with late-stage AMD were more likely to be female and tended to either abstain from alcohol or consume it frequently (≥ 10 times per month), compared to those with moderate intake (1–10 times per month). Lower BMI was also observed in this group (all *P* < 0.05).

It is worth noting that the average daily caffeine intake across the entire population was 205 ± 216 mg. While participants with early AMD consumed less caffeine daily than those without AMD, the difference was not statistically significant (207 ± 218 mg vs.189 ± 178 mg, *P* = 0.4). However, participants with late AMD had significantly lower caffeine intake compared to those without AMD (207 ± 218 mg vs. 102 ± 110 mg, *P* = 0.001).

**Table 1:** Characteristics of participants stratified by AMD stages in the 2005–2008 NHANES (*n* = 4986)

Characteristic	*N* ^a^	Overall *N* = 107,633,905^b^	No AMD *N* = 100,550,354^b^	Early AMD *N* = 6,155,422^b^	Late AMD *N* = 928,129^b^	*p*-value
Age Groups	4986					< 0.001
< 45		664 (16%)	654 (17%)	10 (5.6%)	0 (0%)	
45–64		2594 (59%)	2501 (61%)	89 (34%)	4 (10%)	
>=65		1728 (25%)	1447 (22%)	238 (61%)	43 (90%)	
Sex	4986					0.056
Female		2476 (53%)	2290 (53%)	154 (52%)	32 (75%)	
Male		2510 (47%)	2312 (47%)	183 (48%)	15 (25%)	
Race	4986					0.002
Non-Hispanic White		2739 (78%)	2464 (78%)	233 (84%)	42 (93%)	
Non-Hispanic Black		1009 (9.4%)	974 (9.8%)	32 (3.5%)	3 (4.9%)	
Mexican American		748 (5.1%)	705 (5.2%)	42 (4.8%)	1 (0.5%)	
Other Race		490 (7.1%)	459 (7.1%)	30 (7.3%)	1 (1.1%)	
Education Level	4986					0.14
< High School		659 (6.1%)	599 (5.8%)	54 (9.7%)	6 (11%)	
High School/High School Grad/GED		2001 (38%)	1840 (38%)	143 (41%)	18 (36%)	
> High School		2326 (56%)	2163 (57%)	140 (49%)	23 (53%)	
Poverty	4986					0.7
Not Poor		4222 (91%)	3896 (91%)	287 (91%)	39 (88%)	
Poor		764 (9.2%)	706 (9.2%)	50 (8.9%)	8 (12%)	
Smoking Status	4986					0.037
Current Smoker		1012 (21%)	953 (21%)	52 (16%)	7 (17%)	
Former Smoker		1623 (31%)	1469 (31%)	136 (40%)	18 (41%)	
Never Smoker		2351 (48%)	2180 (49%)	149 (43%)	22 (42%)	
Alcohol Consumption	4986					0.2
Non-drinker		1562 (27%)	1430 (27%)	108 (31%)	24 (47%)	
1–5 drinks/month		2285 (46%)	2124 (47%)	147 (42%)	14 (28%)	
> 5 drinks/month		1139 (26.7%)	1048 (26.7%)	82 (27.3%)	9 (24%)	
Body Mass Index	4986	29 ± (7)	29 ± (7)	29 ± (6)	26 ± (4)	< 0.001
Diabetes History	4986	995 (14%)	915 (14%)	74 (19%)	6 (17%)	0.15
Hypertension History	4986	2753 (50%)	2484 (49%)	235 (64%)	34 (67%)	0.001
Cardiovascular Disease History	4986	744 (12%)	634 (11%)	93 (26%)	17 (35%)	< 0.001
Cataract Operation	4986	616 (9.3%)	487 (7.9%)	97 (23%)	32 (66%)	< 0.001
Glaucoma History	4986	286 (4.6%)	245 (4.3%)	36 (9.4%)	5 (12%)	< 0.001
Total Calorie Intake, kcal/d	4986	2,039 ± (804)	2,053 ± (809)	1,892 ± (711)	1,553 ± (485)	< 0.001
Dietary Caffeine Intake, 100 mg/d	4986	2.05 ± (2.16)	2.07 ± (2.18)	1.89 ± (1.78)	1.02 ± (1.10)	0.004

Notes: Data are presented as n (weighted %) for categorical variables and as weighted mean ± standard error (SE) for continuous variables

^a^ Total number of unweighted individuals with non-missing data

^b^ Total weighted counts for all individuals and for subgroups categorized as no AMD, early AMD, and late AMD

Statistical analyses: Survey-weighted Pearson’s χ² test (Rao-Scott adjustment) for categorical variables and survey-weighted Kruskal-Wallis test for continuous variables

NHANES, National Health and Nutrition Examination Survey; AMD, age-related macular degeneration; High School/High School Grad/GED, High School/High School Graduate/General Educational Development or Equivalent

#### Association between daily dietary caffeine intake and the incidence of early and late AMD

To further evaluate the association between daily caffeine intake and different stages of AMD, we performed multivariable logistic regression analyses, adjusting for potential confounders identified in previous studies and in the initial section of our analysis [44, 45]. The results are summarized in Table 2. Across all models, daily dietary caffeine intake showed no significant association with early AMD occurrence (fully adjusted odds ratios [ORs] = 1.010, 95% CI: [0.923 ~ 1.106], *P* = 0.815). However, higher daily caffeine intake was significantly linked to a lower incidence of late AMD (OR = 0.616, 95% CI: [0.432–0.878], *P* = 0.009), and this inverse association remained consistent after successive adjustments: Model 1 adjusted for demographic characteristics, Model 2 additionally included health-related lifestyle factors, Model 3 further accounted for systemic and ocular conditions (fully adjusted ORs = 0.647, 95% CI: [0.437–0.956], *P* = 0.032). These findings suggest that higher dietary caffeine intake may be protective against late-stage AMD, independent of major confounding factors.

**Table 2:** Logistic regression analysis of daily dietary caffeine intake (per 0.1 g) and the incidence of AMD

Model	Early AMD	Late AMD
Crude^a^ OR (95%CI)	0.959(0.896 ~ 0.1026)	0.616(0.432 ~ 0.878)
p-value	0.213	0.009
Model 1^b^ OR (95%CI)	1.007(0.940 ~ 1.079)	0.682(0.473 ~ 0.984)
p-value	0.829	0.042
Model 2^c^ OR (95%CI)	1.006(0.918 ~ 1.103)	0.658(0.437–0.990)
p-value	0.888	0.045
Model 3^d^ OR (95%CI)	1.010(0.923 ~ 1.106)	0.647(0.437–0.956)
p-value	0.815	0.032

^a^Crude: Unadjusted

^b^Model 1: Adjusted for demographic characteristics including age, sex, race, and poverty level

^c^Model 2: Further adjusted for health-related lifestyle factors, including smoking status, alcohol consumption, body mass index, and total daily calorie intake

^d^Model3: Further adjusted for systemic or ocular conditions, including history of cardiovascular disease, hypertension, cataract surgery and glaucoma

Abbreviations: AMD, age-related macular degeneration; OR, odds ratio; CI, confidence interval

#### Restricted cubic spline analysis

**Fig. 2:**
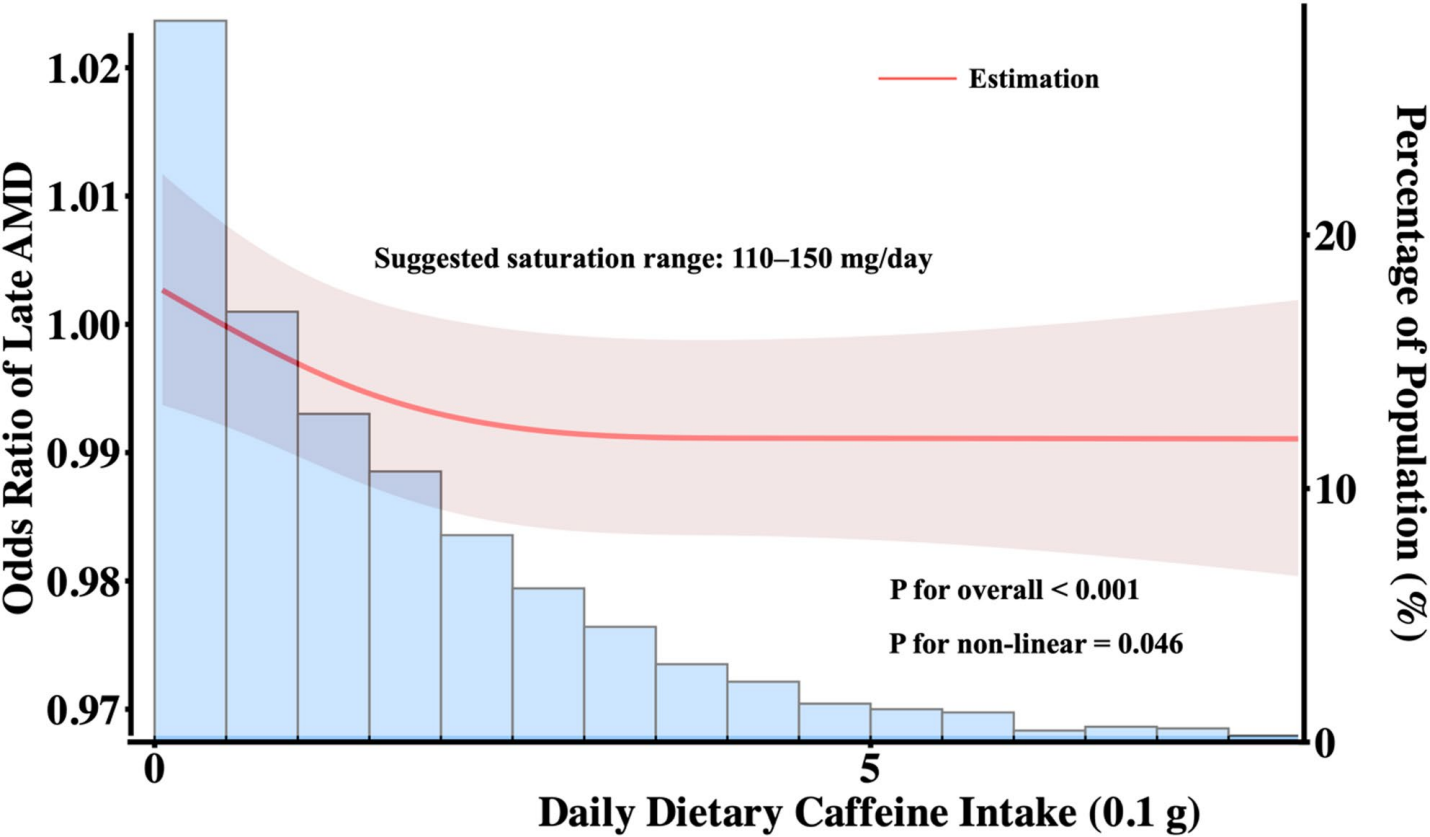
Restricted cubic spline analysis of daily dietary caffeine intake and ORs for late-stage AMD. Notes: The solid lines represent the adjusted odds ratios (ORs) for late AMD across different levels of daily dietary caffeine intake, with 0 mg/day as the reference. Light red shading indicates the 95% confidence interval, and light blue shading represents the distribution of participants across intake levels. The curve demonstrates an L-shaped association, with a precipitous decline in risk at lower intake levels. The inflection point, identified via the maximum curvature method, indicates a saturation threshold at approximately 110–150 mg/day, after which the protective association plateaus. The y-axis displays adjusted ORs on a linear scale; absolute effect sizes are modest due to the low prevalence of late AMD

To further investigate the potential nonlinear association between daily dietary caffeine intake and the risk of late-stage AMD, a restricted cubic spline (RCS) model was applied. As shown in Fig. 2, after application of survey weights and full adjustment for potential confounders—including age, sex, race, poverty-income ratio, smoking status, alcohol consumption, BMI, total daily calorie intake, and medical history of cardiovascular disease, hypertension, cataract surgery, and glaucoma—daily dietary caffeine intake exhibited a nonlinear, L-shaped association with the prevalence of late AMD (P for overall < 0.001, P for nonlinear = 0.046). The spline curve demonstrated a steep decline in the odds of late AMD at lower levels of caffeine intake, followed by a gradual flattening at higher intake levels. Using the maximum curvature method, an apparent plateau in the association was observed at approximately 110–150 mg/day. Beyond this range, the magnitude of the association remained relatively stable, with no further substantial reduction in late AMD risk.

### Mendelian randomization analysis

#### Causal effects of genetically predicted coffee and tea consumption on AMD subtypes

**Fig. 3:**
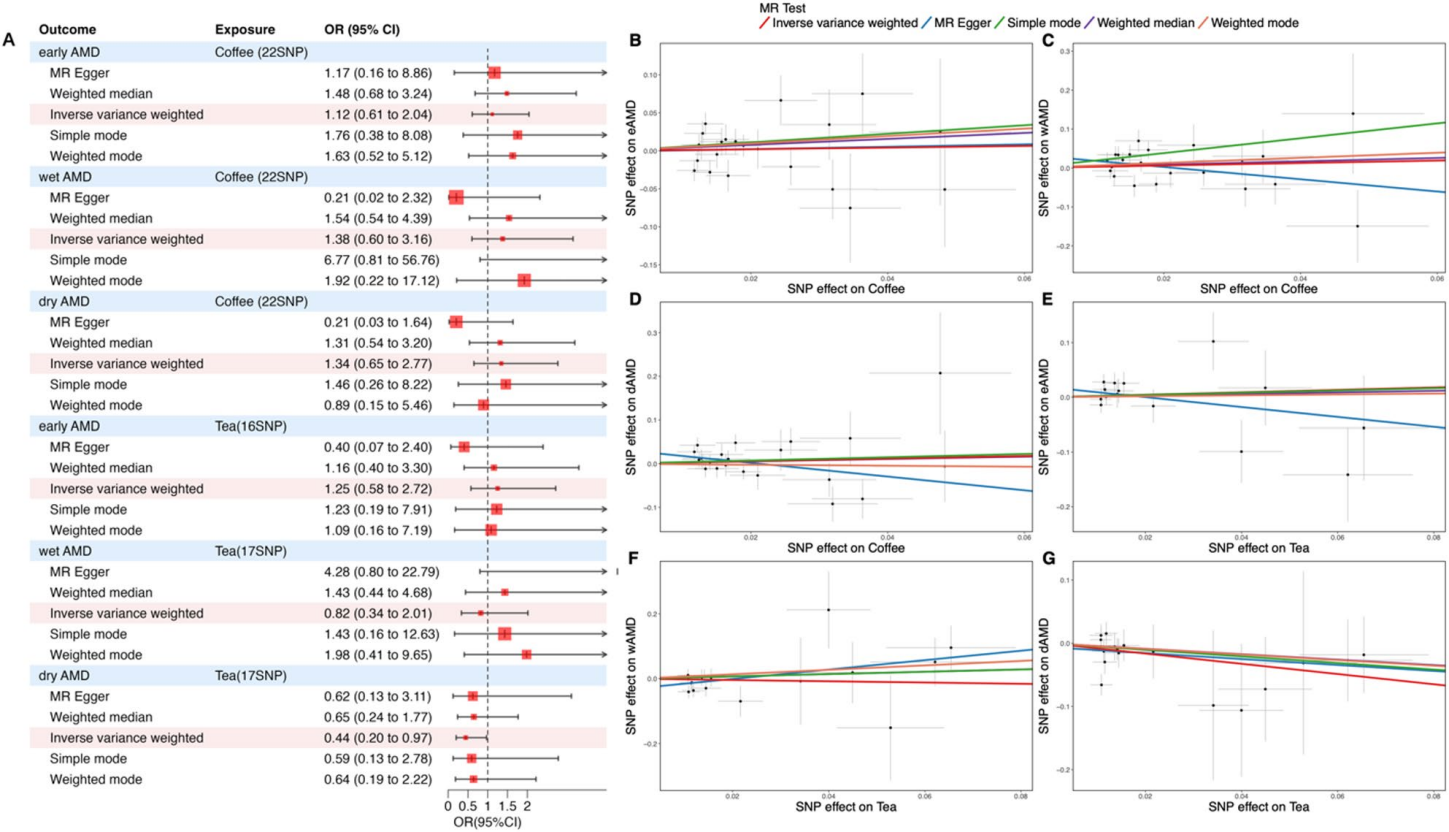
Forest and scatter plots depicting the causal association between coffee/tea consumption and AMD subtypes. Notes: **A**. Forest plot summarizing IVW estimates (light red shading) for the effects of coffee and tea consumption on early, wet, and dry (including geographic atrophy) AMD. **B**–**G**. Scatter plots depicting individual SNP associations for coffee (**B**–**D**) and tea consumption (**E**–**G**) with early AMD (**B**, **E**), wet AMD (**C**, **F**), and dry AMD (including geographic atrophy) (**D**, **G**)

Given the observational association between caffeine intake and late-stage AMD, we applied two-sample Mendelian randomization (MR) to assess potential causal effects of coffee and tea consumption on three AMD subtypes: early AMD, wet AMD, and dry AMD (including geographic atrophy). As shown in Fig. 3A, the IVW model indicated no significant causal association between coffee or tea consumption and early AMD (coffee consumption, OR = 1.12, 95% CI: [0.61–2.04], *P* = 0.7244; tea consumption, OR = 1.25, 95% CI: [0.58–2.72], *P* = 0.5724), and the evidence became even less suggestive of a causal link after adjusting for bias and type I error due to sample overlap (Table S3). These null findings are consistent with our cross-sectional analysis, which also showed lack of association between caffeine intake and early-stage AMD.

Regarding more advanced AMD subtypes, coffee consumption did not significantly associate with either wet AMD or dry AMD, including geographic atrophy (wet AMD, OR = 1.38, 95% CI: [0.60–3.16], *P* = 0.4489; dry AMD, including geographic atrophy, OR = 1.34, 95% CI: [0.65–2.77], *P* = 0.4237). Tea consumption, however, showed a statistically significant inverse association with the risk of dry AMD, including geographic atrophy (OR = 0.44, 95% CI: [0.20–0.97], *P* = 0.0418). Although alternative MR methods did not achieve statistical significance, scatter plots revealed consistent effect directions with the IVW results (Fig. 3B–G), supporting the IVW-based inference [41]. Reverse MR analyses revealed no evidence of reverse causality between AMD subtypes and coffee or tea consumption (Figure S1). Scatter plots detail these relationships (Figure S2), with Table S4 providing SNP data analyzed by MR. This aligns with our RCS findings, as tea typically delivers a moderate caffeine dose near the intake level associated with lowest AMD risk.

#### Mediation mendelian randomization

Given the causal association between tea consumption and dry AMD (including geographic atrophy) demonstrated above, we further conducted a two-step MR analysis to explore immune cell types potentially mediating this relationship [10, 46–48].

**Fig. 4:**
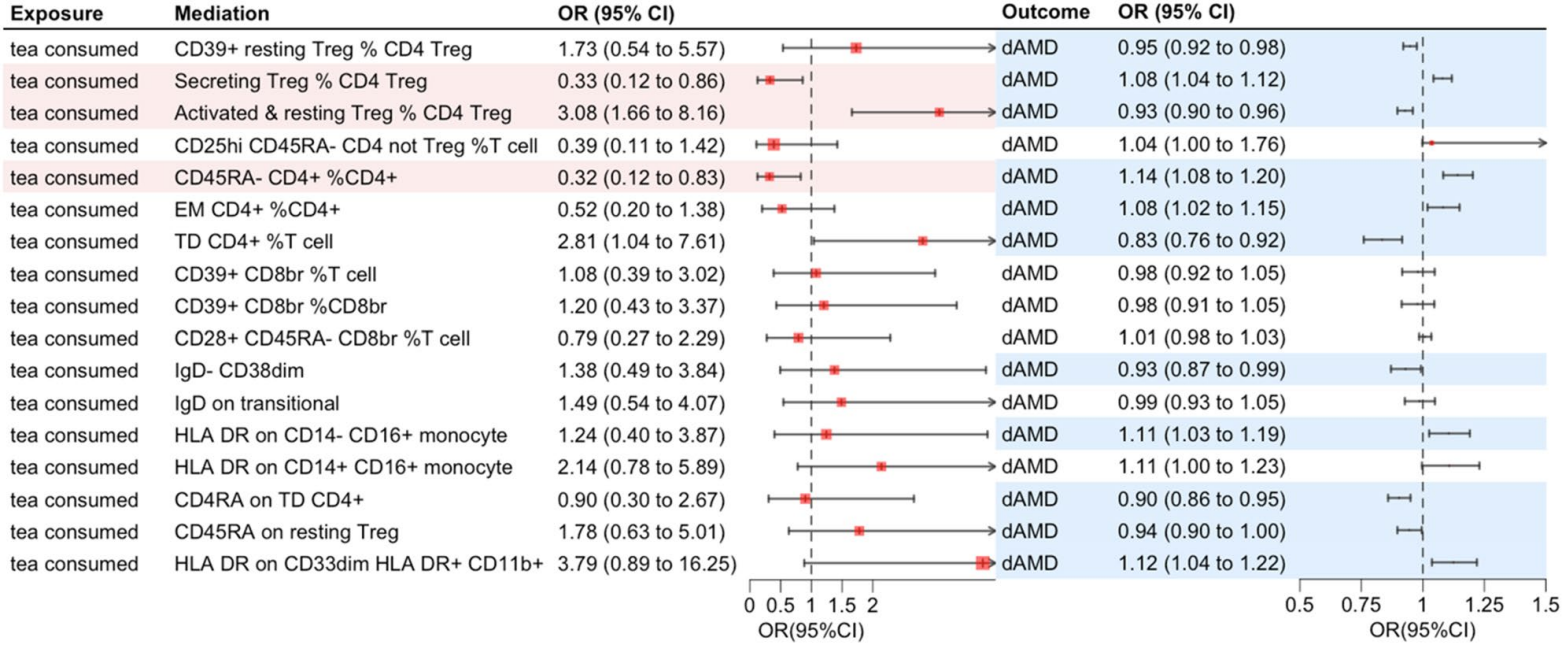
Forest plot of immune cell types linking tea consumption and dry AMD. Notes: Light red shading highlights immune cell types with statistically significant associations with tea consumption, as estimated by the inverse-variance weighted (IVW) method (*P* < 0.05), with directionally consistent results from other MR methods. Light blue shading indicates immune cell types with statistically significant associations with dry AMD (including geographic atrophy) based on IVW estimates, with concordant directions in supplementary MR methods

Building upon Wei et al.‘s identification of 17 immune cell types significantly associated with AMD, we first re-evaluated their causal relationships with dry AMD using two-sample MR. Significant causal associations were confirmed for 11 cell types (Fig. 4, scatter plots in Figure S3). Among these, four cell types showed increased risk associations with dry AMD: Secreting Treg % CD4 Treg (OR = 1.08, 95% CI: [1.04–1.12], *P* < 0.001), CD45RA− CD4+ %CD4+ (OR = 1.14, 95% CI: [1.08–1.20], *P* < 0.001), EM CD4+ %CD4+ (OR = 1.08, 95% CI: [1.02–1.15], *P* = 0.01), HLA DR on CD14 − CD16+ monocyte (OR = 1.11, 95% CI: [1.03–1.19], *P* = 0.009), and HLA DR on CD33dim HLA DR+ CD11b+ (OR = 1.12, 95% CI: [1.04–1.22], *P* = 0.004). Seven cell types demonstrated protective associations: CD39 + resting Treg % CD4 Treg (OR = 0.95, 95% CI: [0.92–0.98], *P* < 0.001), Activated & Resting Treg % CD4 Treg (OR = 0.93, 95% CI: [0.90–0.96], *P* < 0.001), TD CD4+ %T cell (OR = 0.83, 95% CI: [0.76–0.92], *P* < 0.001), IgD− CD38dim (OR = 0.93, 95% CI: [0.87–0.99], *P* = 0.028), CD4RA on TD CD4+ (OR = 0.90, 95% CI: [0.86–0.95], *P* < 0.001), CD45RA on Resting Treg (OR = 0.94, 95% CI: [0.90-1.00], *P* = 0.03). However, leave-one-out analysis identified a highly influential SNP for IgD− CD38dim (Supplemental Fig. 7K); exclusion of this SNP rendered the association nonsignificant (*P* = 0.845, from an original *P* = 0.028). Therefore, 10 immune cell types demonstrated robust causal associations with dry AMD (including geographic atrophy), largely consistent with Wei et al.‘s specific findings for dry AMD subtype.

We then evaluated the causal effects of tea consumption on these 17 immune cell types. Of these, three showed significant associations (Fig. 4): reduced Secreting Treg % CD4 + Treg, a subset involved in producing cytokines such as TGF-β [49] and IL-17 [50, 51] (OR = 0.33, 95% CI: [0.12–0.86], *P* = 0.023); increased Activated & Resting Treg % CD4 + Treg, which function to suppress immune cell activation and activity [52] (OR = 3.08, 95% CI: [1.66–8.16], *P* = 0.023); and reduced CD45RA− CD4+ % CD4+, representing memory T cells that serve as a dynamic reservoir of antigen-experienced T lymphocytes [53] (OR = 0.32, 95% CI: [0.12–0.83], *P* = 0.019). Scatter plots illustrating these associations are shown in Figure S4. All three immune cell types were also significantly associated with dry AMD (including geographic atrophy) in both our analysis and the study by Wei et al., supporting their role as immune mediators.

**Fig. 5:**
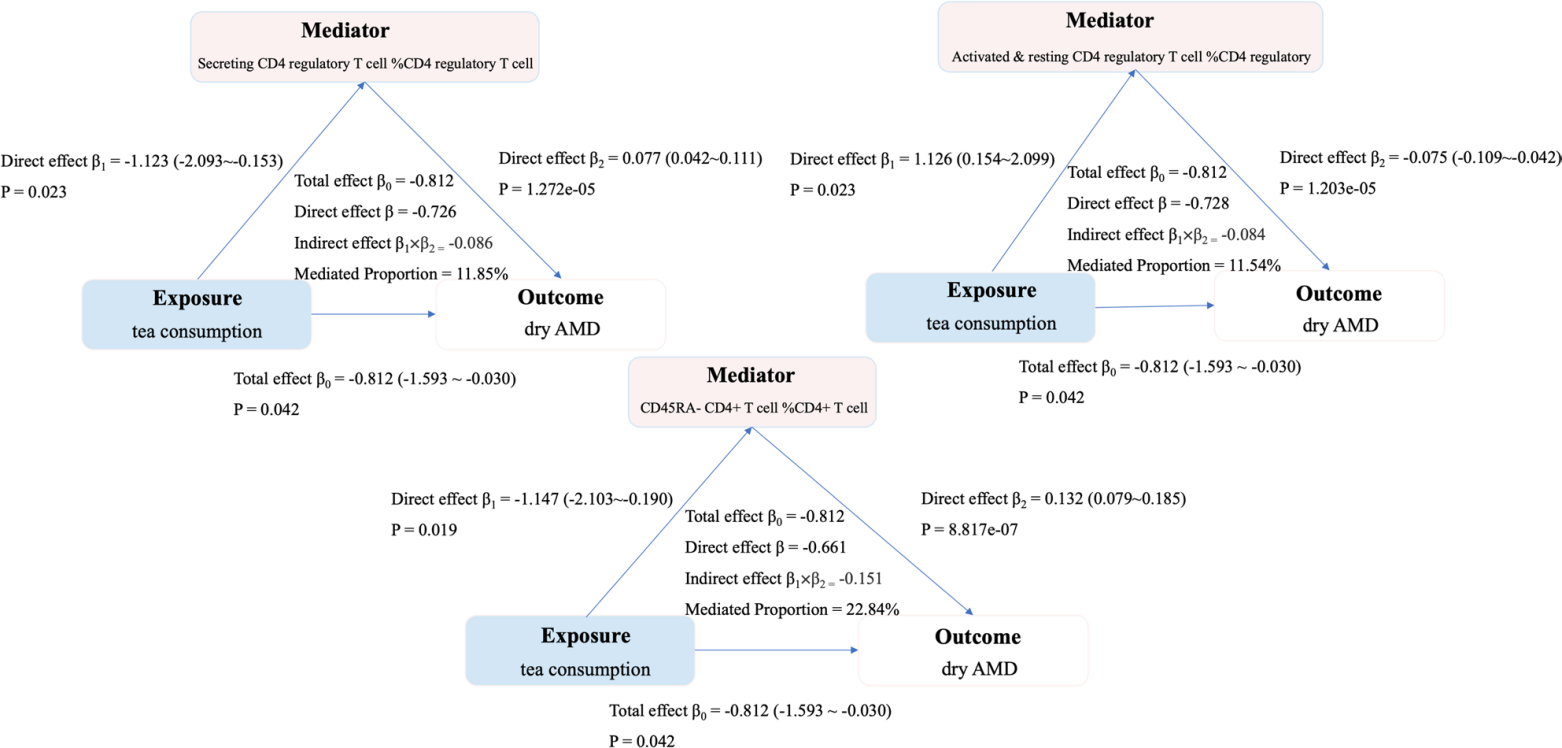
Mediation effect of tea consumption on dry AMD (including geographic atrophy) through immune cell types. Notes: “β_1_” represents the effect of tea consumption on intermediate immune cell types, “β_2_” represents the effect of immune cell types on dry AMD (including geographic atrophy), and “β_0_” represents the total effect of tea consumption on dry AMD (including geographic atrophy). Total, direct, and indirect effects were calculated using the IVW method

Mediation analysis quantified their contributions to tea’s protective effect that could be statistically attributed to specific immunomodulatory pathways: downregulation of Secreting Treg % CD4 + Treg (11.85%), upregulation of Activated & Resting Treg % CD4 + Treg (11.54%), and downregulation of CD45RA− CD4 + T cells (22.84%) (Fig. 5).

### Sensitivity analysis of Mendelian randomization

To ensure the robustness of the MR findings, we performed a comprehensive suite of sensitivity analyses. For the key MR conclusions reported in this study—including the causal effects of tea consumption on dry AMD (including geographic atrophy) and its mediation via immune cell types—no evidence of horizontal pleiotropy (*P* > 0.05 for MR-Egger intercept) or significant heterogeneity (*P* > 0.05 for Cochran’s Q) was observed, as detailed in Table S5–8. Although modest heterogeneity was detected for the association between Secreting Treg % CD4 + Treg and dry AMD (including geographic atrophy), the causal estimate remained reliable owing to the application of a random-effects model. In addition, the I²_GX_ statistic was calculated to evaluate the validity of the no measurement error (NOME) assumption in the MR-Egger analyses. The I²_GX_ values for tea (0.829), coffee (0.758), and the primary immune cell instrumental variables all exceeded the recommended threshold (> 0.60), indicating a low risk of bias due to weak instrument measurement error (Tables S5–S8). Finally, leave-one-out analyses (Figure S5–8) and funnel plot (Figure S9–12) inspections further supported the robustness of the MR results, with no single SNP exerting a disproportionate influence on the overall causal estimates.

## Discussion

### Summary of findings

In this study, we integrated cross-sectional evidence from NHANES with Mendelian randomization (MR) analyses to comprehensively evaluate the associations between dietary caffeine intake, its major dietary sources, and distinct subtypes of age-related macular degeneration (AMD). At the population level, total dietary caffeine intake exhibited a nonlinear, L-shaped inverse association with the prevalence of late-stage AMD, with the protective association reaching a plateau within a moderate intake range of approximately 110–150 mg/day. At the genetic level, MR analyses refined this observation by demonstrating that tea consumption—but not coffee consumption—was associated with a potential causal protective effect against dry AMD, including geographic atrophy. Subsequent two-step MR mediation analyses provided evidence that this association may be partially mediated through immune-related pathways including reductions in the proportions of secretory regulatory CD4 + T cells and CD45RA− CD4 + T cells.

Leveraging the nationally representative design and extensive covariate information available in NHANES, this study both validated and extended prior epidemiological findings. While Chiu et al. observed only a general association between beverage consumption patterns and AMD in the AREDS study [22], our study provides more precise evidence for this protective association through an independent quantitative assessment of caffeine. The Beaver Dam Eye Study found no association between caffeine and early AMD [20], a finding that is highly consistent with the null results observed in our study, jointly supporting the “late-specific” nature of the protective effect. Additionally, the Coimbra study reported a lower risk for individuals with a daily intake of ≥ 78.8 mg (though limited by insufficient adjustment for confounders such as physical activity) [21], which falls within the effective range for significant risk reduction in late AMD identified by our RCS curve.

Although the NHANES analysis provides important population-level epidemiological evidence, its cross-sectional design cannot fully exclude the possibility that changes in health status influence dietary behavior. In this context, the genetic analyses offer critical complementary validation. Because genetic variants are determined early in life, the observed association between tea consumption and dry AMD is unlikely to be explained by reverse causation. Therefore, the concordance in effect direction between observational and genetic analyses substantially reduces the likelihood that the findings are attributable to bias inherent to any single methodological approach.

It should be noted that the outcome definitions differ between the two analytical frameworks: the NHANES outcome (“late AMD”) represents a composite of late-stage wet and dry forms, whereas the MR outcome (“dry AMD/geographic atrophy”) reflects a more specific disease subtype. Nevertheless, these approaches are not contradictory but rather form a complementary and sequential line of evidence. First, both the observational and genetic analyses consistently showed no significant association between caffeine or tea intake and early AMD, supporting the interpretation that the protective association is confined to later disease stages. Furthermore, although “dry AMD” in the MR analysis may encompass a spectrum ranging from early dry AMD to geographic atrophy, the endpoint defined by the FinnGen data was mainly driven by clinical diagnoses involving visual impairment, rendering it phenotypically closer to late-stage geographic atrophy as assessed in NHANES [2]. Accordingly, the NHANES analysis identifies a protective association with late AMD at the population level, while the MR analysis further refines this signal, suggesting that the effect is more pronounced for late-stage dry AMD, including geographic atrophy.

### Focus on specific foods: the role of coffee and tea in AMD prevention

These findings are consistent with the broader shift in nutritional epidemiology from isolated nutrient–based analyses toward whole-food and source-specific exposure assessments [27, 28]. Our results underscore the importance of considering caffeine carriers rather than caffeine intake alone. Accumulating evidence suggests that the health effects of caffeine vary by dietary source. For instance, caffeine derived from coffee has been more strongly associated with reduced risks of coronary artery disease and type 2 diabetes [54], whereas caffeine from tea has been linked to delayed coronary artery calcification [55] and shows a linear inverse association with Parkinson’s disease risk [14]. In this context, the differential effects of coffee and tea observed in the MR analyses may reflect a combination of dose–response characteristics, processing-related byproducts, and source-specific bioactive components.

First, the nonlinear dose–response relationship observed in our analysis may partially explain the absence of a protective association for coffee. As demonstrated in the restricted cubic spline analysis (Fig. 2), dietary caffeine intake followed an L-shaped curve, with the protective association plateauing beyond approximately 150 mg/day. A standard cup of black tea (approximately 70 mg of caffeine) allows individuals to remain within this moderate intake range with relative ease. In contrast, a single cup of coffee typically contains substantially higher caffeine levels (approximately 113–247 mg/cup, based on NHANES FNDDS and U.S. FDA data, https://www.fda.gov/consumers/consumer-updates/spilling-beans-how-much-caffeine-too-much, accessed November 30, 2024), increasing the likelihood of exceeding the saturation threshold without additional protective benefit. Excessive caffeine exposure has been associated with metabolic stress and neurotoxicity in experimental and epidemiological studies [55], which may offset potential benefits. Similar saturation patterns have been reported in studies of cardiovascular, metabolic, and neurodegenerative diseases [56–58].

Second, coffee—particularly highly processed forms such as instant coffee—may introduce potentially harmful compounds generated during high-temperature roasting and industrial processing. Recent MR analyses have reported no causal association between overall coffee intake and AMD, while instant coffee consumption has been associated with an increased risk of AMD [26]. In parallel, observational studies have linked instant coffee intake to reduced retinal nerve fiber layer thickness [59]. Harmful byproducts such as acrylamide [60] and advanced glycation end products (AGEs) [61], along with additives like sugar and non-dairy creamer [26], may neutralize caffeine’s benefits by promoting neuroinflammation [60, 61].

In contrast, the observed protective association of tea may be attributable to its distinctive profile of bioactive compounds and their potential synergistic interactions with caffeine. Tea catechins—particularly epigallocatechin-3-gallate (EGCG)—have demonstrated potent antioxidant properties [62] and have been shown to attenuate lipid peroxidation–induced photoreceptor degeneration in animal models [63]. Mechanistic studies further indicate that EGCG may protect retinal pigment epithelial (RPE) cells by modulating autophagy pathways and restoring JNK1 and c-Jun phosphorylation, thereby inhibiting light-induced apoptosis [64, 65]. By preserving calcium homeostasis and suppressing the unfolded protein response, EGCG may also alleviate age-related endoplasmic reticulum stress, a process implicated in AMD progression [66]. In vitro evidence suggests that EGCG may prevent geographic atrophy by inhibiting the activation of cGMP-AMP synthase (cGAS) and mitochondrial type I interferon (IFN) expression [67]. Beyond catechins, other tea-specific constituents may contribute to retinal protection. Theaflavins, which are characteristic of black tea, exhibit antioxidant capacity comparable to that of catechins [68], while L-theanine has been shown to mitigate excitotoxic injury in retinal ganglion cells [69]. Collectively, these flavonoids have been reported to downregulate key inflammatory transcription factors, including NF-κB and AP-1, suppress pro-inflammatory cytokines such as IL-1β, TNF-α, and IL-6, and inhibit enzymes involved in inflammatory signaling, including inducible nitric oxide synthase, cyclooxygenase-2, and lipoxygenase [70]. Through these pathways, tea consumption may help maintain a relatively low-inflammatory retinal microenvironment that is favorable for cellular survival and function.

### Mechanistic insights into tea’s protective effects on dry AMD (Including geographic atrophy)

In the mediation MR analysis, tea consumption significantly influenced 3 of the 10 immune cell types that were causally associated with dry AMD. The observation that nearly one-third of the total causal effect could be statistically attributed to these immune mediators suggests a biologically plausible link between tea consumption and dry AMD. Although these mediation findings require confirmation through functional experiments and clinical studies, the identified immune cell changes are consistent with both the known immunomodulatory effects of tea-derived bioactive compounds and the established immunopathological features of AMD. Together, these results provide a preliminary and coherent mechanistic framework for interpreting the observed associations.

Among the immune cell subsets affected by tea consumption, secretory regulatory T cells (Tregs) and activated/resting Tregs represent functionally distinct and complementary regulatory T cell populations. Secreting Tregs typically exhibit pro-inflammatory characteristics, shifting toward a Th17-like function rather than the classical immunosuppressive phenotype. Previous studies have established that in the immunopathological context of AMD, Tregs play a critical role in maintaining retinal immune homeostasis and suppressing chronic inflammation. Activated/resting Tregs can attenuate damage to the RPE and choroid, maintaining photoreceptor integrity by suppressing local inflammation [71, 72]. Conversely, secreting Tregs release TGF-β and IL-17, which accelerate RPE senescence and damage [49–51, 73, 74].

The MR-mediated pattern observed in this study—characterized by a reduction in secretory Tregs and a relative increase in activated/resting Tregs—is highly concordant with existing evidence on how tea polyphenols, particularly EGCG, regulate T cell plasticity. Mechanistically, EGCG has been shown to inhibit STAT3 signaling, a key molecular switch driving the transdifferentiation of Tregs toward a Th17-like, pro-inflammatory phenotype. Inhibition of STAT3 phosphorylation suppresses downstream expression of IL-17 and related inflammatory mediators [75]. In addition, EGCG can interfere with the mTOR–HIF-1α metabolic axis, thereby modulating the balance between Th17 and Treg differentiation [76]. At the epigenetic level, EGCG exhibits DNA methyltransferase (DNMT) inhibitory activity, which helps maintain hypomethylation of the Foxp3 promoter and stabilizes the immunosuppressive phenotype of Tregs, limiting pro-inflammatory differentiation of CD4⁺ T cells [77]. In contrast to tea polyphenols, caffeine appears to exert immunomodulatory effects primarily by modulating immune cell function rather than directly adjusting Treg abundance. Caffeine antagonizes adenosine A2A receptor signaling, thereby influencing the intensity of Treg-mediated immunosuppression and indirectly shaping the functional balance among T cell subsets [78]. Taken together, these mechanisms provide a plausible immunological explanation for the observed association between tea consumption, suppression of pro-inflammatory Treg phenotypes, and reduced risk of dry AMD.

In addition to regulatory T cell subsets, tea consumption was associated with changes in CD45RA⁻CD4⁺ T cells, which largely represent antigen-experienced memory T cells and are widely regarded as markers of immunosenescence. Elevated levels of CD45RA⁻CD4⁺ memory T cells have been consistently reported in patients with AMD and have been linked to disease progression [79–81]. Experimental evidence suggests that caffeine can inhibit the differentiation of naïve T cells into memory T cells by interfering with adenosine receptor–mediated signaling pathways [82]. Moreover, tea polyphenols have been shown to suppress excessive T cell activation and limit the accumulation of memory T cells under chronic inflammatory conditions [83]. Such coordinated regulation may reduce the infiltration of senescent T cells into retinal tissue and contribute to improved immune microenvironment homeostasis [7, 84].

In summary, the immune cell alterations identified through mediation MR analysis are biologically consistent with both the immunopathology of AMD and the established immunomodulatory actions of tea polyphenols and caffeine. Nevertheless, the current evidence is derived primarily from genetic inference, observational epidemiology, and preclinical studies. Future investigations should directly evaluate the effects of tea-derived compounds on retinal T cell infiltration and function using AMD animal models and human retinal samples, as well as explore potential interaction effects between caffeine and catechins in modulating retinal immune responses.

### Limitations of the study

This study has several limitations that should be acknowledged. First, the cross-sectional design of the NHANES analysis precludes causal inference and is inherently susceptible to reverse causality (e.g., dietary modification following illness) as well as recall bias in dietary assessment. Although we adjusted for a wide range of potential confounders and incorporated Mendelian randomization (MR) as a complementary analytical approach, residual confounding cannot be fully excluded. Second, the relatively small number of late-stage AMD cases in NHANES (*n* = 47) raises the possibility of sparsity bias. To mitigate overfitting, we applied a simplified restricted cubic spline (RCS) model with the minimum number of knots and reported a suggested saturation range rather than a precise threshold. Nevertheless, these dose–response findings should be interpreted cautiously. Third, the outcome definitions differed between the two analytical frameworks: NHANES defined “late AMD” as a mixed outcome including both exudative AMD and geographic atrophy, whereas the MR analysis focused specifically on dry AMD (including geographic atrophy). Rather than viewing this as a contradiction, we interpret the results as complementary: the NHANES analysis identifies a population-level protective association with late-stage disease, while the MR analysis refines this signal toward a dry/atrophic phenotype. Fourth, due to the limited availability of genetic instruments for coffee and tea consumption, a relatively lenient SNP selection threshold (*P* < 5 × 10⁻⁶) was applied in the MR analysis. Although all selected instruments demonstrated adequate strength based on F-statistics, this approach may still affect the precision of causal estimates. Fifth, the mediation analysis is based on genetic inference and does not constitute direct mechanistic evidence. Functional studies are therefore required to validate whether the immune pathways identified—particularly those involving regulatory and memory T cell subsets—are directly modulated by tea-derived bioactive components in the context of AMD. Finally, NHANES dietary data do not allow isolation of the independent observational effects of non-caffeine components, such as tea polyphenols, and the genetic datasets used in the MR analyses were primarily derived from European-ancestry populations. Accordingly, the generalizability of these findings to other ethnic groups and dietary patterns warrants further investigation.

## Conclusions

By integrating nationally representative observational data from NHANES with genetic causal inference through Mendelian randomization, this study provides a comprehensive evaluation of the relationship between caffeine intake, its primary dietary sources, and late-stage age-related macular degeneration (AMD). In the observational analysis, higher dietary caffeine intake was associated with a lower prevalence of late-stage AMD in a nonlinear, L-shaped pattern, with the protective association plateauing at moderate intake levels. The MR analysis further localized this protective association to dry AMD (including geographic atrophy) and indicated that a statistically significant causal association was observed for tea consumption, but not for coffee consumption. These findings suggest that caffeine content alone may be insufficient to explain the observed protective association and that the combined effects of caffeine with non-caffeine bioactive compounds present in tea may be more relevant. Moreover, mediation MR analyses identified specific immune cell subsets—particularly secretory regulatory CD4^+^ T cells and CD45RA⁻ CD4^+^ T cells—as potential mediators, providing a biologically plausible immunological context for the association between tea intake and reduced dry AMD risk. Although the precise effector components and molecular mechanisms remain to be elucidated, this study offers novel epidemiological and genetic evidence supporting the importance of dietary source–specific effects in AMD and may inform future research on targeted dietary prevention strategies.

## Supplementary Information

Below is the link to the electronic supplementary material.

Supplementary Material 1

## Data Availability

The data used in this study, including publicly available genome-wide association study (GWAS) summary statistics and NHANES datasets, are accessible online. Specific dataset names and access links are provided in the main text or Supplementary Materials. NHANES data are available at https://www.cdc.gov/nchs/nhanes/. GWAS summary statistics for tea and coffee consumption were obtained from the UK Biobank and can be accessed by searching the relevant traits at https://gwas.mrcieu.ac.uk/. GWAS data for dry and wet AMD were obtained from the FinnGen consortium at https://r11.finngen.fi/. GWAS data for early AMD were derived from the study by Winkler et al., DOI: 10.1186/s12920-020-00760-7. Summary statistics for immune cell phenotypes are available through the GWAS Catalog at https://www.ebi.ac.uk/gwas/ using the corresponding IDs. All datasets were accessed on November 30, 2024.

## Abbreviations

AMD: Age-related macular degeneration
MR: Mendelian randomization
GWAS: Genome-wide association study
Treg: Regulatory T cell
NHANES: National health and nutrition examination survey
MEC: Mobile examination center
FNDDS: Food and nutrient database for dietary studies
GED: General educational development
PIR: Poverty income ratio
BMI: Body mass index
CVD: Cardiovascular disease
HbA1c: Glycated hemoglobin
IV: Instrumental variable
IAMDGC: International AMD genomics consortium
NOME: No measurement error (assumption in MR-Egger analysis)
GA: Geographic atrophy
LOO: Leave-one-out
SE: Standard error
IVW: Inverse-variance weighted
RCS: Restricted cubic spline
OR: Odds ratio
AGEs: Advanced glycation end products
EGCG: Epigallocatechin-3-gallate
RPE: Retinal pigment epithelium
cGAS: Cyclic gmp–amp synthase
IFN: Interferon
Treg: Regulatory t cell
DNMT: Dna methyltransferase
dAMD: Dry age-related macular degeneration
wAMD: Wet age-related macular degeneration
eAMD: Early age-related macular degeneration
