# Supplementary material for: Caffeine intake and late dry age-related macular degeneration: tea’s protective role—insights from NHANES and Mendelian randomization
Source: Int J Retina Vitreous. 2026 Feb 16;12:43. doi: 10.1186/s40942-026-00813-6 (PMC13014731; doi:10.1186/s40942-026-00813-6)
Supplement: Supplementary file 1 — Supplementary Material 1 [file 40942_2026_813_MOESM1_ESM.docx]

**Caffeine Intake and Late Dry Age-Related Macular Degeneration: Tea's Protective Role—Insights from NHANES and Mendelian Randomization**

Hongli Yang^†^, Boshi Liu^†^, Yunxi Zhang, Zhanhe Zhang, Huang Tan, Xiaorong Li^*^

**Supplementary Materials**

Graphical Abstract

Table S1. Detailed Information on GWAS Data for Traits Included in the Study.

Table S2. Demographic and Clinical Characteristics of Participants Without AMD and With Late-stage AMD in the 2005–2008 NHANES Survey (n=4649)

Table S3. Bias and Type I Error Rates in Mendelian Randomization Due to Sample Overlap Between Caffeinated Beverage Consumption and Early AMD.

Table S4. Genetic Variants Employed in Mendelian Randomization Analyses of Caffeinated Beverage Consumption, AMD Subtypes, and Subsequent Immune Cell-Type Mediation.

Table S5. Mendelian Randomization and Sensitivity Analysis Results for the Association Between Caffeinated Beverage Consumption and AMD Subtypes.

Table S6. Inverse Mendelian Randomization and Sensitivity Analysis Results for the Association Between AMD Subtypes and Caffeinated Beverage Consumption.

Table S7. Mendelian Randomization and Sensitivity Analysis Results for the Association Between Immune Cell Types and Dry AMD (Including Geographic Atrophy).

Table S8. Mendelian Randomization and Sensitivity Analysis Results for the Association Between Tea Consumption and Immune Cell Types.

Figure S1. Forest Plot of the Reverse Mendelian Randomization Analysis Assessing the Causal Effects of AMD Subtypes on Caffeinated Beverage Consumption.

Figure S2. Scatter Plots of the Reverse Mendelian Randomization Analysis Assessing the Causal Effects of AMD Subtypes on Caffeinated Beverage Consumption.

Figure S3. Scatter Plot of the Association Between 17 Immune Cell Types and Dry AMD (Including Geographic Atrophy).

Figure S4. Scatter Plots Showing the Associations Between Tea Consumption and 17 Immune Cell Types.

Figure S5. Leave-One-Out Analysis of Caffeinated Beverage Consumption on AMD Subtypes by SNP Exclusion.

Figure S6. Leave-One-Out Analysis of AMD Subtypes on Caffeinated Beverage Consumption by SNP Exclusion.

Figure S7. Leave-One-Out Analysis of Immune Cell Type SNPs on Dry AMD (Including Geographic Atrophy) by SNP Exclusion.

Figure S8. Leave-One-Out Analysis of Tea Consumption on Immune Cell Types by SNP Exclusion.

Figure S9. Funnel Plot for Mendelian Randomization of Caffeinated Beverage Consumption and AMD Subtypes.

Figure S10. Funnel Plot for Mendelian Randomization of AMD Subtypes and Caffeinated Beverage Consumption.

Figure S11. Funnel Plot for Mendelian Randomization of Immune Cell Types and Dry AMD (Including Geographic Atrophy).

Figure S12. Funnel Plot for Mendelian Randomization of Tea Consumption and Immune Cell Types.

STROBE-MR checklist of recommended items to address in reports of Mendelian randomization studies

**Graphical Abstract**

**
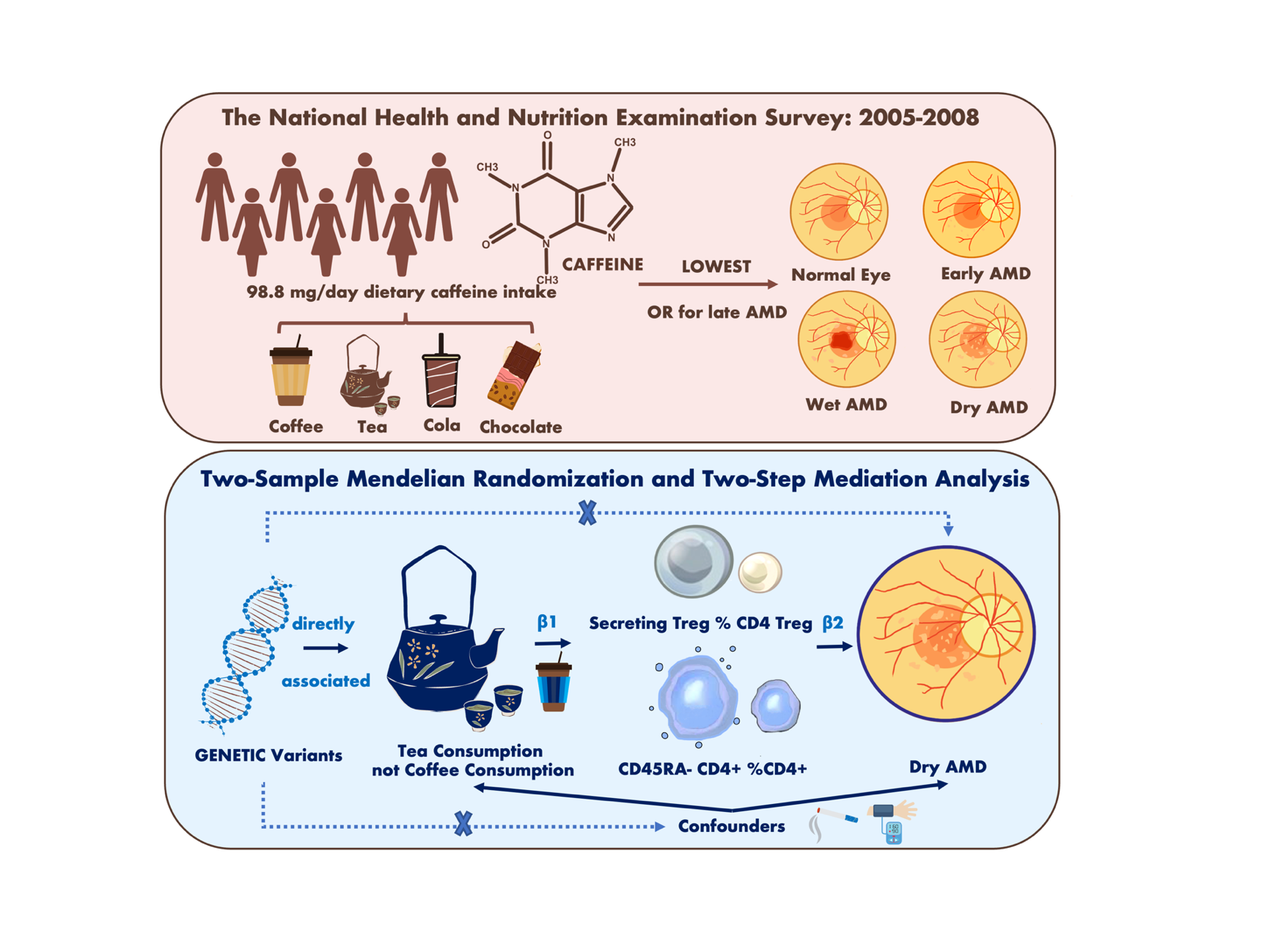
**

**Table S1. Detailed Information on GWAS Data for Traits Included in the Study.**

| Trait | Consortium | Population | Sample Size | Number of cases | GWAS ID | Release time |
| --- | --- | --- | --- | --- | --- | --- |
| coffee consumed | MRC-IEU | European | 64949 | 45788 | ukb-b-9508 | 2018 |
| tea consumed | MRC-IEU | European | 64949 | 51690 | ukb-b-17988 | 2018 |
| early AMD | IAMDGC, UKBB | European | 105258 | 14034 | ebi-a-GCST010723 | 2020 |
| wet AMD | FinnGen R11 | European | 306075 | 7589 | finngen_R11_WET_AMD | 2024 |
| dry AMD | FinnGen R11 | European | 306042 | 5890 | finngen_R11_DRY_AMD | 2024 |
| CD39+ resting Treg % CD4 Treg | NA | European | 3437 | NA | ebi-a-GCST90001485 | 2020 |
| Secreting Treg % CD4 Treg | NA | European | 3437 | NA | ebi-a-GCST90001493 | 2020 |
| Activated & resting Treg % CD4 Treg | NA | European | 3437 | NA | ebi-a-GCST90001499 | 2020 |
| CD25hi CD45RA- CD4 not Treg %T cell | NA | European | 3437 | NA | ebi-a-GCST90001512 | 2020 |
| CD45RA- CD4+ %CD4+ | NA | European | 3427 | NA | ebi-a-GCST90001535 | 2020 |
| EM CD4+ %CD4+ | NA | European | 3427 | NA | ebi-a-GCST90001543 | 2020 |
| TD CD4+ %T cell | NA | European | 3427 | NA | ebi-a-GCST90001547 | 2020 |
| CD39+ CD8br %T cell | NA | European | 3440 | NA | ebi-a-GCST90001670 | 2020 |
| CD39+ CD8br %CD8br | NA | European | 3440 | NA | ebi-a-GCST90001671 | 2020 |
| CD28+ CD45RA- CD8br %T cell | NA | European | 3440 | NA | ebi-a-GCST90001691 | 2020 |
| IgD- CD38dim | NA | European | 3657 | NA | ebi-a-GCST90001735 | 2020 |
| IgD on transitional | NA | European | 3657 | NA | ebi-a-GCST90001828 | 2020 |
| HLA DR on CD14- CD16+ monocyte | NA | European | 3621 | NA | ebi-a-GCST90001984 | 2020 |
| HLA DR on CD14+ CD16+ monocyte | NA | European | 3618 | NA | ebi-a-GCST90002007 | 2020 |
| CD4RA on TD CD4+ | NA | European | 2903 | NA | ebi-a-GCST90002099 | 2020 |
| CD45RA on resting Treg | NA | European | 2919 | NA | ebi-a-GCST90002102 | 2020 |
| HLA DR on CD33dim HLA DR+ CD11b+ | NA | European | 1634 | NA | ebi-a-GCST90002110 | 2020 |

**Table S2. Demographic and Clinical Characteristics of Participants Without AMD and With Late-stage AMD in the 2005–2008 NHANES Survey (n=4649)**

| **Characteristic** | **N^a^** | **Overall  N = 101,478,483^b^** | **No AMD  N = 100,550,354^b^** | **Late AMD N = 928,129^b^** | **p-value** |
| --- | --- | --- | --- | --- | --- |
| Age Groups | 4649 |  |  |  | <0.001 |
| <45 |  | 654 (17%) | 654 (17%) | 0 (0%) |  |
| 45-64 |  | 2505 (60%) | 2501 (61%) | 4 (10%) |  |
| >=65 |  | 1490 (23%) | 1447 (22%) | 43 (90%) |  |
| Sex | 4649 |  |  |  | 0.015 |
| Female |  | 2322 (54%) | 2290 (53%) | 32 (75%) |  |
| Male |  | 2327 (46%) | 2312 (47%) | 15 (25%) |  |
| Race | 4649 |  |  |  | 0.018 |
| Non-Hispanic White |  | 2506 (78%) | 2464 (78%) | 42 (93%) |  |
| Non-Hispanic Black |  | 977 (9.8%) | 974 (9.8%) | 3 (4.9%) |  |
| Mexican American |  | 706 (5.1%) | 705 (5.2%) | 1 (0.5%) |  |
| Other Race |  | 460 (7.1%) | 459 (7.1%) | 1 (1.1%) |  |
| Poverty | 4649 |  |  |  | 0.5 |
| Not Poor |  | 3935 (91%) | 3896 (91%) | 39 (88%) |  |
| Poor |  | 714 (9.2%) | 706 (9.2%) | 8 (12%) |  |
| Education level | 4649 |  |  |  | 0.5 |
| <High School |  | 605 (5.9%) | 599 (5.8%) | 6 (11%) |  |
| High School/High School Grad/GED |  | 1858 (38%) | 1840 (38%) | 18 (36%) |  |
| >High School |  | 2186 (57%) | 2163 (57%) | 23 (53%) |  |
| Smoking Status | 4649 |  |  |  | 0.4 |
| Current Smoker |  | 960 (21%) | 953 (21%) | 7 (17%) |  |
| Former Smoker |  | 1487 (31%) | 1469 (31%) | 18 (41%) |  |
| Never Smoker |  | 2202 (48%) | 2180 (49%) | 22 (42%) |  |
| Alcohol Consumption | 4649 |  |  |  | 0.048 |
| Non-drinker |  | 1454 (27%) | 1430 (27%) | 24 (47%) |  |
| 1-5 drinks/month |  | 2138 (46%) | 2124 (47%) | 14 (28%) |  |
| 5-10 drinks/month |  | 312 (7.7%) | 312 (7.7%) | 0 (0%) |  |
| 10+ drinks/month |  | 745 (19%) | 736 (19%) | 9 (24%) |  |
| Body Mass Index | 4649 | 29 ± (7) | 29 ± (7) | 26 ± (4) | <0.001 |
| Diabetes History | 4649 | 921 (14%) | 915 (14%) | 6 (17%) | 0.6 |
| Hypertension History | 4649 | 2518 (49%) | 2484 (49%) | 34 (67%) | 0.10 |
| Cardiovascular Disease History | 4649 | 651 (11%) | 634 (11%) | 17 (35%) | <0.001 |
| Cataract Operation | 4649 | 519 (8.4%) | 487 (7.9%) | 32 (66%) | <0.001 |
| Glaucoma History | 4649 | 250 (4.4%) | 245 (4.3%) | 5 (12%) | 0.014 |
| Total Calorie Intake, kcal/d | 4649 | 2,048 ± (808) | 2,053 ± (809) | 1,553 ± (485) | <0.001 |
| Dietary Caffeine Intake, 100mg/d | 4649 | 2.06 ± (2.18) | 2.07 ± (2.18) | 1.02 ± (1.10) | 0.001 |
| *Notes:*  Data are presented as n (weighted %) for categorical variables and as weighted mean ± standard error (SE) for continuous variables.  ^a^ Total number of unweighted individuals with non-missing data.  ^b^ Total weighted counts for all individuals and for subgroups categorized as no AMD and late AMD.  Statistical analyses: Survey-weighted Pearson’s χ² test (Rao-Scott adjustment) for categorical variables and survey-weighted Kruskal-Wallis test for continuous variables.  NHANES, National Health and Nutrition Examination Survey; AMD, age-related macular degeneration; High School/High School Grad/GED, High School/High School Graduate/General Educational Development or Equivalent. | | | | | |

**Table S3. Bias and Type I Error Rates in Mendelian Randomization Due to Sample Overlap Between Caffeinated Beverage Consumption and Early AMD.**

| Exposure | tea consumed | coffee consumed |
| --- | --- | --- |
| Outcome | early AMD (binary) | early AMD (binary) |
| Coefficient of determination (R^2^) of risk factor on genetic variants | 0.00870314 | 0.005653018 |
| Sample size for exposure | 64949 | 64949 |
| Sample size for outcome | 105258 | 105258 |
| Number of instruments | 22 | 16 |
| Bias of the observational estimate (OLS estimate) ^a^ | 0.815 | 0.815 |
| Proportion of cases | 0.133 | 0.133 |
| Maximum overlap rate | 0.617 | 0.617 |
| Concentration parameter (expected value of F statistic) | 25.91 | 23.07 |
| Conservative value of concentration parameter (lower limit of one-sided 95% confidence interval) | 15.28 | 12.28 |
| The average F statistic of the instrumental variables used in our study | 25.7 | 22.95 |
| Bias using specified value of concentration parameter | 0.019 | 0.021 |
| Type 1 error rate using specified value of concentration parameter | 0.05 | 0.05 |
| Bias using conservative value of concentration parameter | 0.032 | 0.04 |
| Type 1 error rate using conservative value of concentration parameter | 0.05 | 0.06 |

*Note:* ^a^Due to the absence of direct data on the association between coffee or tea consumption and early AMD, the value presented in this table is derived from a cross-sectional study examining the relationship between caffeine intake and early AMD.

**Table S4. Genetic Variants Employed in Mendelian Randomization Analyses of Caffeinated Beverage Consumption, AMD Subtypes, and Subsequent Immune Cell-Type Mediation.**

| Table S4A: Instrumental Variables for Coffee Consumption. | | | | | | | | |
| --- | --- | --- | --- | --- | --- | --- | --- | --- |
| SNP | Effect allele | Other allele | Eaf | Beta | SE | P value | R2 | F |
| rs12094804 | G | A | 0.060116 | -0.0243655 | 0.00532156 | 4.70E-06 | 0.000322671 | 20.96328583 |
| rs1773019 | T | C | 0.030224 | 0.0363082 | 0.00738142 | 8.70E-07 | 0.000372388 | 24.19447652 |
| rs1820987 | T | C | 0.56879 | 0.0117202 | 0.00256059 | 4.70E-06 | 0.000322461 | 20.94964084 |
| rs79750331 | A | G | 0.040739 | 0.0319388 | 0.00657937 | 1.20E-06 | 0.000362692 | 23.5643354 |
| rs62159340 | T | G | 0.192084 | -0.0166569 | 0.00324751 | 2.90E-07 | 0.000404892 | 26.30716139 |
| rs141934938 | A | G | 0.03514 | 0.0314535 | 0.00687517 | 4.80E-06 | 0.00032215 | 20.92944448 |
| rs11939505 | A | G | 0.161076 | -0.016303 | 0.0034478 | 2.30E-06 | 0.000344135 | 22.35823687 |
| rs6860893 | A | G | 0.477557 | -0.0123902 | 0.0025318 | 9.90E-07 | 0.000368608 | 23.94883924 |
| rs6968865 | T | A | 0.626977 | 0.0188985 | 0.00258921 | 2.90E-13 | 0.00081958 | 53.27294981 |
| rs2237526 | T | C | 0.327047 | -0.0129453 | 0.00272564 | 2.00E-06 | 0.000347187 | 22.55661241 |
| rs5022011 | T | A | 0.379825 | 0.0121731 | 0.00264595 | 4.20E-06 | 0.000325781 | 21.16536388 |
| rs117234665 | A | G | 0.015586 | 0.0476878 | 0.0103948 | 4.50E-06 | 0.000323943 | 21.04596897 |
| rs11781400 | G | T | 0.344006 | -0.0133427 | 0.00267022 | 5.80E-07 | 0.000384285 | 24.96778295 |
| rs2065113 | G | A | 0.098808 | -0.0258238 | 0.00426414 | 1.40E-09 | 0.000564365 | 36.67448428 |
| rs12785741 | C | G | 0.323954 | -0.0127401 | 0.0027024 | 2.40E-06 | 0.000342078 | 22.22455305 |
| rs141320788 | C | A | 0.015411 | -0.0483458 | 0.0103999 | 3.30E-06 | 0.000332615 | 21.60955391 |
| rs2472297 | T | C | 0.25771 | 0.017741 | 0.00288014 | 7.30E-10 | 0.000583852 | 37.9415982 |
| rs143924958 | G | T | 0.033553 | -0.0345129 | 0.00741088 | 3.20E-06 | 0.000333815 | 21.68752389 |
| rs17701213 | C | T | 0.1079 | -0.0209821 | 0.00408201 | 2.70E-07 | 0.000406631 | 26.42021837 |
| rs2117885 | C | T | 0.186513 | 0.0150152 | 0.00324606 | 3.70E-06 | 0.000329332 | 21.39615734 |
| rs7245162 | T | G | 0.325919 | 0.0139905 | 0.00270007 | 2.20E-07 | 0.000413204 | 26.84745054 |
| rs8135992 | G | A | 0.203339 | -0.0156977 | 0.00317395 | 7.60E-07 | 0.000376475 | 24.46011612 |
| Table S4B: Instrumental Variables for Tea Consumption. | | | | | | | | |
| SNP | Effect allele | Other allele | Eaf | Beta | SE | P value | R2 | F |
| rs139516017 | A | T | 0.013467 | -0.0528513 | 0.0111624 | 2.20E-06 | 0.000345043 | 22.41727464 |
| rs7420454 | C | T | 0.330221 | -0.0113217 | 0.00236928 | 1.80E-06 | 0.000351451 | 22.8337444 |
| rs3791436 | C | T | 0.758062 | 0.0135848 | 0.00263771 | 2.60E-07 | 0.000408228 | 26.52400082 |
| rs7634654 | C | T | 0.013295 | -0.0449409 | 0.00976092 | 4.10E-06 | 0.000326278 | 21.19769557 |
| rs57163864 | C | T | 0.648707 | -0.0107483 | 0.00234848 | 4.70E-06 | 0.000322399 | 20.94558303 |
| rs57657064 | A | C | 0.158624 | -0.0144438 | 0.00311122 | 3.40E-06 | 0.00033173 | 21.55202663 |
| rs116180006 | G | T | 0.024076 | -0.0341317 | 0.00735699 | 3.50E-06 | 0.000331283 | 21.52296673 |
| rs55695458 | T | C | 0.137942 | -0.0155207 | 0.00324954 | 1.80E-06 | 0.000351119 | 22.8121102 |
| rs2151175 | T | C | 0.400081 | 0.0108439 | 0.00228473 | 2.10E-06 | 0.000346719 | 22.52619637 |
| rs75619489 | T | A | 0.016752 | -0.0399661 | 0.00873667 | 4.80E-06 | 0.000322092 | 20.92561853 |
| rs4905945 | C | T | 0.637235 | 0.0118368 | 0.00234912 | 4.70E-07 | 0.000390765 | 25.38895588 |
| rs76893898 | A | C | 0.006982 | -0.062114 | 0.0134551 | 3.90E-06 | 0.000328012 | 21.31040788 |
| rs183619175 | A | G | 0.008225 | 0.0654371 | 0.01349 | 1.20E-06 | 0.000362155 | 23.52939799 |
| rs17747955 | C | T | 0.491714 | -0.0107157 | 0.00224572 | 1.80E-06 | 0.000350433 | 22.76756084 |
| rs6074498 | T | C | 0.062776 | -0.0216333 | 0.00467704 | 3.70E-06 | 0.000329297 | 21.39390209 |
| rs7276934 | A | G | 0.67683 | 0.0141242 | 0.00254446 | 2.80E-08 | 0.000474196 | 30.81222692 |
| rs13054829 | T | A | 0.282959 | -0.0115488 | 0.0025061 | 4.10E-06 | 0.000326861 | 21.23555198 |
| Table S4C: Instrumental Variables for Early AMD. | | | | | | | | |
| SNP | Effect allele | Other allele | Eaf | Beta | SE | P value | R2 | F |
| rs3750847 | T | C | 0.2245 | 0.3838 | 0.0166 | 2.88E-118 | 0.051290636 | 5690.517437 |
| rs247617 | A | C | 0.3231 | 0.0922 | 0.0147 | 3.56E-10 | 0.003718376 | 392.842125 |
| rs11569415 | A | G | 0.2141 | 0.1158 | 0.018 | 1.25E-10 | 0.004512644 | 477.1359984 |
| rs4658046 | T | C | 0.6098 | -0.3213 | 0.014 | 1.47E-116 | 0.049127666 | 5438.144984 |
| rs4844620 | A | G | 0.2135 | -0.0949 | 0.0173 | 4.12E-08 | 0.003024538 | 319.3165432 |
| rs550605 | T | C | 0.9119 | 0.2178 | 0.0246 | 8.47E-19 | 0.007621999 | 808.4228939 |
| rs943080 | T | C | 0.5068 | 0.0797 | 0.0145 | 3.87E-08 | 0.003175458 | 335.3006939 |
| rs13278062 | T | G | 0.5207 | 0.0801 | 0.0142 | 1.69E-08 | 0.003202507 | 338.1660144 |
| Table S4D. Instrument Variables for Wet AMD | | | | | | | | |
| SNP | Effect allele | Other allele | Eaf | Beta | SE | P value | R2 | F |
| rs6677435 | A | G | 0.0693929 | -0.283022 | 0.0418614 | 1.37E-11 | 0.010345507 | 3199.581711 |
| rs1376814 | T | C | 0.260215 | 0.130345 | 0.0216797 | 1.83E-09 | 0.006541187 | 2015.263092 |
| rs76907927 | A | G | 0.0249634 | 0.316749 | 0.0573164 | 3.27E-08 | 0.004884107 | 1502.230282 |
| rs1329424 | G | T | 0.562734 | -0.580514 | 0.0186006 | 1.00E-200 | 0.165845716 | 60853.12597 |
| rs148136314 | T | C | 0.0267952 | -0.539434 | 0.0693871 | 7.59E-15 | 0.015176369 | 4716.658492 |
| rs151064834 | G | A | 0.0441222 | -0.302139 | 0.0512984 | 3.87E-09 | 0.007700219 | 2375.118213 |
| rs6445425 | C | G | 0.35936 | 0.125137 | 0.0200019 | 3.94E-10 | 0.007210166 | 2222.86434 |
| rs115407994 | A | G | 0.0293536 | 0.358059 | 0.0527426 | 1.13E-11 | 0.007305696 | 2252.532651 |
| rs3775221 | G | T | 0.427339 | 0.122201 | 0.0194774 | 3.52E-10 | 0.00730886 | 2253.515382 |
| rs189631141 | T | C | 0.0570004 | -0.267736 | 0.0444082 | 1.65E-09 | 0.00770607 | 2376.9368 |
| rs429608 | A | G | 0.12082 | -0.483585 | 0.0325313 | 5.54E-50 | 0.049681222 | 16001.03146 |
| rs241425 | G | A | 0.521619 | 0.108256 | 0.0193495 | 2.21E-08 | 0.005848726 | 1800.668716 |
| rs7758685 | G | A | 0.582204 | 0.110524 | 0.0197407 | 2.16E-08 | 0.005942684 | 1829.768897 |
| rs142171156 | T | C | 0.122834 | 0.157977 | 0.0288006 | 4.13E-08 | 0.005377967 | 1654.950654 |
| rs10808141 | G | T | 0.318584 | 0.130846 | 0.0205006 | 1.74E-10 | 0.007433395 | 2292.200197 |
| rs113769372 | A | G | 0.0376067 | 0.455008 | 0.0452954 | 9.63E-24 | 0.014986005 | 4656.595312 |
| rs78653376 | T | C | 0.150043 | -0.301594 | 0.0289739 | 2.25E-25 | 0.023200005 | 7269.548794 |
| rs58649964 | A | T | 0.192168 | 0.684354 | 0.0213404 | 1.00E-200 | 0.145409821 | 52078.78709 |
| rs76647508 | C | T | 0.0884351 | 0.274212 | 0.031969 | 9.70E-18 | 0.012123141 | 3756.102042 |
| rs12262149 | T | C | 0.2859 | 0.144039 | 0.020969 | 6.46E-12 | 0.00847156 | 2615.06943 |
| rs12880842 | G | A | 0.606601 | 0.12807 | 0.0199381 | 1.33E-10 | 0.007828187 | 2414.900958 |
| rs2043085 | C | T | 0.580598 | 0.143841 | 0.0197572 | 3.33E-13 | 0.010076308 | 3115.478205 |
| rs3825991 | A | C | 0.485587 | 0.125068 | 0.0193643 | 1.06E-10 | 0.007814504 | 2410.646549 |
| rs12149545 | A | G | 0.275923 | 0.116943 | 0.0212471 | 3.71E-08 | 0.005464509 | 1681.728357 |
| rs2230199 | C | G | 0.18236 | 0.241147 | 0.0239491 | 7.56E-24 | 0.017341451 | 5401.418396 |
| rs2364542 | A | G | 0.32711 | 0.113127 | 0.0204826 | 3.33E-08 | 0.005633787 | 1734.119782 |
| rs429358 | C | T | 0.180086 | -0.220133 | 0.0274246 | 1.00E-15 | 0.014310294 | 4443.58358 |
| Table S4E. Instrument Variables for Dry AMD | | | | | | | | |
| SNP | Effect allele | Other allele | Eaf | Beta | SE | P value | R2 | F |
| rs12021875 | G | T | 0.105663 | -0.166751 | 0.028845 | 7.43E-09 | 0.005255221 | 1616.804653 |
| rs6677435 | A | G | 0.0693564 | -0.332712 | 0.0369421 | 2.13E-19 | 0.014290152 | 4436.76025 |
| rs114213310 | A | T | 0.0170061 | 0.364217 | 0.0592902 | 8.10E-10 | 0.004435126 | 1363.372723 |
| rs1329424 | G | T | 0.562669 | -0.546478 | 0.0165086 | 1.00E-200 | 0.146973357 | 52729.56766 |
| rs148136314 | T | C | 0.0267608 | -0.520315 | 0.0612262 | 1.92E-17 | 0.014102022 | 4377.514473 |
| rs12141142 | G | C | 0.044071 | -0.361764 | 0.0457853 | 2.76E-15 | 0.011027047 | 3412.345607 |
| rs76114727 | A | G | 0.170145 | 0.125418 | 0.0224055 | 2.17E-08 | 0.004441924 | 1365.471806 |
| rs10033900 | C | T | 0.45765 | -0.109781 | 0.0172006 | 1.74E-10 | 0.005982703 | 1841.966484 |
| rs429608 | A | G | 0.120811 | -0.41959 | 0.0284669 | 3.59E-49 | 0.037399774 | 11890.53 |
| rs9273319 | T | C | 0.518222 | 0.116234 | 0.0172252 | 1.50E-11 | 0.006746199 | 2078.629713 |
| rs7758685 | G | A | 0.582284 | 0.111354 | 0.0174698 | 1.84E-10 | 0.006031948 | 1857.2201 |
| rs1064583 | G | A | 0.363248 | -0.101347 | 0.0179618 | 1.68E-08 | 0.004751441 | 1461.073205 |
| rs17777642 | A | G | 0.364459 | 0.104622 | 0.0176656 | 3.17E-09 | 0.005070704 | 1559.747356 |
| rs139604450 | A | G | 0.0621171 | 0.234811 | 0.033752 | 3.48E-12 | 0.006424313 | 1978.8091 |
| rs78653376 | T | C | 0.149981 | -0.228431 | 0.0253499 | 2.04E-19 | 0.013304696 | 4126.673299 |
| rs61871744 | C | T | 0.241613 | 0.611681 | 0.017977 | 1.00E-200 | 0.137116953 | 48631.4716 |
| rs2043085 | C | T | 0.580554 | 0.110193 | 0.0174646 | 2.80E-10 | 0.005913665 | 1820.584234 |
| rs12438812 | T | C | 0.486535 | 0.113908 | 0.0171016 | 2.73E-11 | 0.006482811 | 1996.945398 |
| rs2230199 | C | G | 0.182383 | 0.253584 | 0.02112 | 3.27E-33 | 0.019178205 | 5984.061458 |
| rs6509172 | T | C | 0.343463 | 0.104058 | 0.0179117 | 6.27E-09 | 0.004883375 | 1501.842263 |
| rs429358 | C | T | 0.180058 | -0.195589 | 0.0241315 | 5.27E-16 | 0.011295733 | 3496.440866 |
| Table S4F. Instrument Variables for CD39+ Resting Treg % CD4 Treg | | | | | | | | |
| SNP | Effect allele | Other allele | Eaf | Beta | SE | P value | R2 | F |
| rs34645541 | A | C | 0.0095 | 0.7843 | 0.1289 | 1.29E-09 | 0.010656781 | 37.00034764 |
| rs116794175 | C | T | 0.0042 | 1.311 | 0.1905 | 6.98E-12 | 0.013592281 | 47.33284757 |
| rs113435341 | G | A | 0.0032 | 2.267 | 0.2171 | 3.79E-25 | 0.030749626 | 108.9759347 |
| rs11579717 | A | G | 0.0044 | 1.718 | 0.1835 | 1.35E-20 | 0.02486896 | 87.60348655 |
| rs12712610 | G | A | 0.2975 | 0.201 | 0.02719 | 1.83E-13 | 0.015651042 | 54.61612816 |
| rs11890666 | A | G | 0.2866 | -0.1612 | 0.02787 | 7.94E-09 | 0.009639839 | 33.43515545 |
| rs11818051 | T | C | 0.0317 | 0.6998 | 0.0719 | 4.24E-22 | 0.026822702 | 94.67543403 |
| rs4918969 | A | G | 0.4267 | 0.8426 | 0.02078 | 1.00E-200 | 0.323583558 | 1643.232563 |
| rs35909109 | T | C | 0.0464 | 0.3811 | 0.05993 | 2.30E-10 | 0.011628667 | 40.4144387 |
| rs182702572 | A | C | 0.0295 | 0.5464 | 0.07402 | 1.95E-13 | 0.015606744 | 54.45909614 |
| rs1410600 | T | C | 0.4069 | -0.2216 | 0.02536 | 3.59E-18 | 0.021732961 | 76.31118946 |
| rs12778837 | C | T | 0.3595 | 0.1965 | 0.02651 | 1.55E-13 | 0.015733981 | 54.91017948 |
| rs111534725 | T | G | 0.0409 | 0.8056 | 0.06291 | 1.06E-36 | 0.045538471 | 163.8878485 |
| rs17111341 | C | T | 0.0666 | 0.6853 | 0.05013 | 1.86E-41 | 0.0515694 | 186.7726426 |
| rs77520132 | A | G | 0.0464 | 0.6067 | 0.05819 | 4.44E-25 | 0.030658339 | 108.6421833 |
| Table S4G. Instrument Variables for Secreting Treg % CD4 Treg | | | | | | | | |
| SNP | Effect allele | Other allele | Eaf | Beta | SE | P value | R2 | F |
| rs11579717 | A | G | 0.0044 | -1.776 | 0.1774 | 2.93E-23 | 0.028334525 | 100.1672847 |
| rs116794175 | C | T | 0.0042 | -1.417 | 0.1841 | 1.78E-14 | 0.016944572 | 59.2078557 |
| rs113435341 | G | A | 0.0032 | -2.54 | 0.2093 | 3.14E-33 | 0.041089197 | 147.1892817 |
| rs413431 | G | A | 0.8154 | 0.2955 | 0.03106 | 3.31E-21 | 0.025659218 | 90.46056104 |
| rs6751481 | C | T | 0.4709 | -0.2879 | 0.02374 | 3.53E-33 | 0.041034156 | 146.9836777 |
| Table S4H. Instrument Variables for Activated & Resting Treg % CD4 Treg | | | | | | | | |
| SNP | Effect allele | Other allele | Eaf | Beta | SE | P value | R2 | F |
| rs113435341 | G | A | 0.0032 | 2.569 | 0.2103 | 1.28E-33 | 0.041611349 | 149.1409388 |
| rs11579717 | A | G | 0.0044 | 1.815 | 0.1783 | 5.33E-24 | 0.029266518 | 103.5613687 |
| rs116794175 | C | T | 0.0042 | 1.427 | 0.1851 | 1.62E-14 | 0.016998468 | 59.39943554 |
| rs6751481 | C | T | 0.4709 | 0.2886 | 0.02387 | 5.49E-33 | 0.040796152 | 146.0948897 |
| rs413431 | G | A | 0.8154 | -0.2936 | 0.03123 | 9.54E-21 | 0.025070433 | 88.33144668 |
| Table S4I. Instrument Variables for CD25hi CD45RA- CD4 not Treg %T cell | | | | | | | | |
| SNP | Effect allele | Other allele | Eaf | SE | Beta | P value | R2 | F |
| rs4497244 | A | G | 0.0033 | 0.2116 | -1.396 | 4.86E-11 | 0.012505318 | 43.4997458 |
| rs116794175 | C | T | 0.0042 | 0.197 | -0.9442 | 1.72E-06 | 0.00663931 | 22.95845978 |
| rs11579717 | A | G | 0.0044 | 0.1911 | -1.078 | 1.83E-08 | 0.009173482 | 31.80265345 |
| rs17666424 | A | G | 0.0912 | 0.04507 | 0.212 | 2.66E-06 | 0.006396321 | 22.11280392 |
| rs139048853 | C | T | 0.0646 | 0.05164 | -0.2433 | 2.56E-06 | 0.006417064 | 22.18497583 |
| rs419099 | G | A | 0.2912 | 0.02955 | -0.1371 | 3.61E-06 | 0.00622399 | 21.51330569 |
| rs6912413 | G | C | 0.1263 | 0.03881 | -0.1789 | 4.18E-06 | 0.006144367 | 21.23638572 |
| rs2004605 | T | C | 0.35 | 0.02686 | 0.1253 | 3.19E-06 | 0.006291722 | 21.74890191 |
| rs12006014 | A | G | 0.1158 | 0.03935 | 0.183 | 3.42E-06 | 0.006253294 | 21.6152327 |
| rs61839660 | T | C | 0.0543 | 0.05532 | 0.5758 | 5.32E-25 | 0.030557751 | 108.2745001 |
| rs143283244 | A | G | 6.00E-04 | 0.4951 | -2.27 | 4.70E-06 | 0.006079084 | 21.009372 |
| rs12458491 | T | A | 0.0188 | 0.09269 | 0.4275 | 4.13E-06 | 0.006151023 | 21.25953118 |
| rs2702579 | T | C | 0.049 | 0.05782 | -0.2771 | 1.72E-06 | 0.006638116 | 22.95430173 |
| rs148871284 | T | C | 0.0343 | 0.07002 | 0.3269 | 3.15E-06 | 0.006301742 | 21.7837597 |
| Table S4J. Instrument Variables for CD45RA- CD4+ %CD4+ | | | | | | | | |
| SNP | Effect allele | Other allele | Eaf | Beta | SE | P value | R2 | F |
| rs146620586 | T | G | 0.0032 | -2.021 | 0.2655 | 3.46E-14 | 0.016626769 | 57.90953099 |
| rs11579717 | A | G | 0.0044 | -1.081 | 0.1759 | 8.87E-10 | 0.010900479 | 37.74558671 |
| rs12712610 | G | A | 0.2969 | -0.1902 | 0.02599 | 3.13E-13 | 0.015387208 | 53.52478575 |
| Table S4K. Instrument Variables for EM CD4+ %CD4+ | | | | | | | | |
| SNP | Effect allele | Other allele | Eaf | Beta | SE | P value | R2 | F |
| rs146620586 | T | G | 0.0032 | -1.617 | 0.2699 | 2.30E-09 | 0.010365139 | 35.87242548 |
| rs11579717 | A | G | 0.0044 | -0.9188 | 0.1786 | 2.82E-07 | 0.007663443 | 26.44999141 |
| rs114825362 | C | G | 0.0201 | -0.4464 | 0.08643 | 2.55E-07 | 0.00772392 | 26.6603482 |
| rs114348846 | A | G | 0.2628 | -0.135 | 0.02714 | 6.93E-07 | 0.007168189 | 24.72830347 |
| rs114174358 | T | C | 0.0018 | -1.26 | 0.2626 | 1.66E-06 | 0.006673132 | 23.00901982 |
| rs9267484 | T | A | 0.1729 | 0.2057 | 0.04288 | 1.67E-06 | 0.006670193 | 22.9988172 |
| rs35900954 | A | G | 0.0848 | 0.2059 | 0.04362 | 2.43E-06 | 0.006459706 | 22.26833992 |
| rs4959028 | G | A | 0.2288 | 0.2111 | 0.03351 | 3.35E-10 | 0.011447568 | 39.66195179 |
| rs9269702 | A | G | 0.7954 | -0.2094 | 0.03833 | 5.03E-08 | 0.008633677 | 29.82786629 |
| rs7798224 | C | G | 0.0251 | -0.3626 | 0.07866 | 4.18E-06 | 0.006162388 | 21.23704986 |
| rs28844071 | A | C | 0.4021 | 0.1143 | 0.02439 | 2.88E-06 | 0.006367669 | 21.94902997 |
| rs439782 | A | G | 0.1698 | 0.155 | 0.03244 | 1.84E-06 | 0.006617654 | 22.81645541 |
| rs73060045 | G | A | 0.1023 | 0.1986 | 0.03908 | 3.93E-07 | 0.007479537 | 25.81046624 |
| rs72744142 | A | G | 0.15 | 0.159 | 0.03367 | 2.44E-06 | 0.006465127 | 22.2871475 |
| rs157386 | A | G | 0.1029 | 0.1903 | 0.03972 | 1.74E-06 | 0.006653433 | 22.94064204 |
| rs75010615 | T | C | 0.002 | 1.402 | 0.3037 | 4.06E-06 | 0.006180164 | 21.2986911 |
| rs6046769 | G | A | 0.6265 | -0.1192 | 0.02465 | 1.38E-06 | 0.006777215 | 23.37034561 |
| Table S4L. Instrument Variables for TD CD4+ %T cell | | | | | | | | |
| SNP | Effect allele | Other allele | Eaf | Beta | SE | P value | R2 | F |
| rs17583875 | A | G | 0.0029 | 1.299 | 0.2259 | 9.52E-09 | 0.009556556 | 33.04702064 |
| rs13024840 | A | T | 0.2578 | 0.2049 | 0.02827 | 5.23E-13 | 0.015097719 | 52.50235343 |
| rs9271536 | T | A | 0.7798 | -0.291 | 0.03325 | 3.30E-18 | 0.021861942 | 76.55069713 |
| rs113243185 | C | G | 0.1996 | 0.2854 | 0.03605 | 3.28E-15 | 0.017960249 | 62.63886371 |
| rs62400929 | G | A | 0.1087 | 0.3169 | 0.04734 | 2.55E-11 | 0.012907194 | 44.78519222 |
| Table S4M. Instrument Variables for CD39+ CD8br %T cell | | | | | | | | |
| SNP | Effect allele | Other allele | Eaf | Beta | SE | P value | R2 | F |
| rs11818051 | T | C | 0.0317 | 0.6769 | 0.07397 | 9.35E-20 | 0.023764781 | 83.69224555 |
| rs12778618 | C | T | 0.0462 | 0.8332 | 0.06081 | 1.23E-41 | 0.05175029 | 187.6272639 |
| rs72812685 | A | G | 0.0483 | 0.6459 | 0.05873 | 1.13E-27 | 0.03396601 | 120.8809869 |
| rs12778837 | C | T | 0.3594 | 0.2205 | 0.0273 | 9.08E-16 | 0.018611207 | 65.19875808 |
| rs111534725 | T | G | 0.041 | 0.7307 | 0.06471 | 4.66E-29 | 0.035741315 | 127.4332751 |
| rs17111341 | C | T | 0.0667 | 0.6237 | 0.0518 | 9.80E-33 | 0.04043951 | 144.8903289 |
| Table S4N. Instrument Variables for CD39+ CD8br %CD8br | | | | | | | | |
| SNP | Effect allele | Other allele | Eaf | Beta | SE | P value | R2 | F |
| rs72812685 | A | G | 0.0483 | 0.6238 | 0.05887 | 7.75E-26 | 0.031607887 | 112.2147895 |
| rs12778618 | C | T | 0.0462 | 0.8186 | 0.06103 | 5.07E-40 | 0.049700287 | 179.8059962 |
| rs2901833 | A | G | 0.383 | 0.2205 | 0.02662 | 1.73E-16 | 0.019555356 | 68.5722682 |
| rs111534725 | T | G | 0.041 | 0.7675 | 0.06467 | 7.27E-32 | 0.039333731 | 140.7662289 |
| rs17111341 | C | T | 0.0667 | 0.6402 | 0.05184 | 2.59E-34 | 0.042452497 | 152.4223958 |
| rs11818051 | T | C | 0.0317 | 0.6977 | 0.07403 | 7.75E-21 | 0.025170518 | 88.77064531 |
| Table S4O. Instrument Variables for CD28+ CD45RA- CD8br %T cell | | | | | | | | |
| SNP | Effect allele | Other allele | Eaf | Beta | SE | P value | R2 | F |
| rs7530076 | A | G | 0.9749 | 0.3879 | 0.08339 | 3.42E-06 | 0.006250719 | 21.62514565 |
| rs116794175 | C | T | 0.0042 | -1.073 | 0.1963 | 4.99E-08 | 0.008610821 | 29.8611325 |
| rs17583875 | A | G | 0.0029 | -2.154 | 0.2288 | 8.60E-21 | 0.025117318 | 88.5781871 |
| rs3117751 | G | A | 0.5685 | 0.1175 | 0.0257 | 4.98E-06 | 0.006039765 | 20.89088875 |
| rs60220946 | T | C | 0.0212 | -0.5154 | 0.0867 | 3.04E-09 | 0.01016842 | 35.31815948 |
| rs76557351 | C | G | 0.0808 | -0.2373 | 0.0462 | 2.95E-07 | 0.007610884 | 26.3668929 |
| rs71621309 | C | T | 0.0089 | 0.599 | 0.1284 | 3.22E-06 | 0.006286751 | 21.75058925 |
| rs1450147 | T | C | 0.7064 | -0.1281 | 0.02787 | 4.42E-06 | 0.006103892 | 21.11406002 |
| rs114547596 | G | A | 7.00E-04 | -3.353 | 0.7077 | 2.25E-06 | 0.006483142 | 22.43448712 |
| rs16996915 | A | C | 0.2762 | -0.1384 | 0.02867 | 1.45E-06 | 0.006728624 | 23.28971769 |
| rs34933238 | G | A | 0.0339 | 0.374 | 0.06857 | 5.24E-08 | 0.008573869 | 29.73187753 |
| rs4425613 | A | C | 0.4436 | -0.1299 | 0.02621 | 7.54E-07 | 0.007089829 | 24.5488804 |
| rs9378248 | A | G | 0.4339 | 0.1572 | 0.03075 | 3.38E-07 | 0.007539967 | 26.11934447 |
| rs59052071 | T | C | 0.1933 | 0.1945 | 0.03964 | 9.64E-07 | 0.006949997 | 24.06131449 |
| rs1722056 | T | C | 0.1831 | 0.158 | 0.03231 | 1.05E-06 | 0.006903562 | 23.89943795 |
| rs74331648 | A | C | 0.0347 | -0.3166 | 0.06827 | 3.65E-06 | 0.006212936 | 21.493614 |
| rs180683226 | A | G | 0.0385 | -0.3108 | 0.06673 | 3.31E-06 | 0.006266589 | 21.68039549 |
| rs9608041 | T | C | 0.0667 | 0.2542 | 0.05123 | 7.32E-07 | 0.007106351 | 24.60649807 |
| Table S4P. Instrument Variables for IgD- CD38dim | | | | | | | | |
| SNP | Effect allele | Other allele | Eaf | Beta | SE | P value | R2 | F |
| rs112417310 | A | G | 0.0201 | 0.5373 | 0.08896 | 1.70E-09 | 0.009876623 | 36.45915119 |
| rs11643297 | G | A | 0.611 | 0.167 | 0.02538 | 5.48E-11 | 0.011700737 | 43.27251406 |
| rs138920008 | C | G | 0.0041 | -1.514 | 0.2157 | 2.63E-12 | 0.013292749 | 49.23952798 |
| Table S4Q. Instrument Variables for IgD on Transitional | | | | | | | | |
| SNP | Effect allele | Other allele | Eaf | Beta | SE | P value | R2 | F |
| rs1037633 | G | T | 0.124 | 0.234 | 0.03837 | 1.19E-09 | 0.010067663 | 37.17153857 |
| rs55871547 | G | C | 0.2752 | 0.2309 | 0.0279 | 1.76E-16 | 0.01838467 | 68.4544807 |
| rs2018404 | T | C | 0.1325 | -0.2444 | 0.0376 | 9.14E-11 | 0.011421234 | 42.22689363 |
| rs709589 | T | C | 0.4557 | -0.5713 | 0.03022 | 4.00E-76 | 0.089026689 | 357.1921856 |
| Table S4R. Instrument Variables for HLA DR on CD14- CD16+ Monocyte | | | | | | | | |
| SNP | Effect allele | Other allele | Eaf | Beta | SE | P value | R2 | F |
| rs6808893 | T | C | 0.5719 | -0.2043 | 0.02491 | 3.27E-16 | 0.018237578 | 67.22786766 |
| rs6917212 | G | C | 0.777 | -0.476 | 0.03201 | 1.32E-48 | 0.057553375 | 221.0052627 |
| rs61742525 | A | G | 0.0888 | 0.313 | 0.04821 | 9.64E-11 | 0.011506931 | 42.12835199 |
| rs9269109 | C | T | 0.3592 | 0.3511 | 0.02921 | 1.19E-32 | 0.03836883 | 144.3971439 |
| rs116671810 | A | G | 0.0711 | 0.3239 | 0.05515 | 4.66E-09 | 0.009435934 | 34.47393995 |
| rs412492 | T | A | 0.7974 | -0.299 | 0.03392 | 1.83E-18 | 0.021007839 | 77.65881293 |
| rs73499473 | T | C | 0.776 | 0.3801 | 0.02898 | 1.91E-38 | 0.045353722 | 171.9329185 |
| Table S4S. Instrument Variables for HLA DR on CD14+ CD16+ Monocyte | | | | | | | | |
| SNP | Effect allele | Other allele | Eaf | Beta | SE | P value | R2 | F |
| rs144126567 | G | C | 0.1712 | -0.3054 | 0.03316 | 5.39E-20 | 0.02290742 | 84.77521131 |
| rs6934244 | A | C | 0.1551 | 0.4143 | 0.039 | 5.66E-26 | 0.030247737 | 112.7873809 |
| rs9275511 | A | G | 0.6053 | 0.3664 | 0.02907 | 1.07E-35 | 0.04206198 | 158.7744897 |
| rs6917212 | G | C | 0.7769 | -0.5221 | 0.03191 | 4.09E-58 | 0.068894434 | 267.5553487 |
| rs9275639 | T | A | 0.2553 | 0.2756 | 0.0316 | 4.08E-18 | 0.020591109 | 76.02284541 |
| rs62395272 | T | C | 0.2848 | -0.2623 | 0.03321 | 3.77E-15 | 0.016949853 | 62.34744909 |
| Table S4T. Instrument Variables for CD4RA on TD CD4+ | | | | | | | | |
| SNP | Effect allele | Other allele | Eaf | Beta | SE | P value | R2 | F |
| rs11579717 | A | G | 0.0045 | 1.19 | 0.202 | 4.31E-09 | 0.01181362 | 34.68102119 |
| rs541367304 | A | G | 0.0033 | 2.073 | 0.2799 | 1.70E-13 | 0.018544553 | 54.81425303 |
| rs7559619 | G | A | 0.3455 | -0.2144 | 0.02871 | 1.09E-13 | 0.018848305 | 55.72933618 |
| Table S4U. Instrument Variables for CD45RA on Resting Treg | | | | | | | | |
| SNP | Effect allele | Other allele | Eaf | Beta | SE | P value | R2 | F |
| rs138915779 | A | C | 0.0033 | 1.586 | 0.2396 | 4.28E-11 | 0.014788634 | 43.78597983 |
| rs11579717 | A | G | 0.0041 | 1.499 | 0.1979 | 4.74E-14 | 0.019276325 | 57.33423358 |
| rs541367304 | A | G | 0.0029 | 2.693 | 0.2744 | 2.12E-22 | 0.031942689 | 96.25134941 |
| rs13024840 | A | T | 0.2648 | 0.3454 | 0.02873 | 1.57E-32 | 0.047179198 | 144.4360984 |
| rs11124653 | A | C | 0.5925 | -0.2555 | 0.02636 | 6.84E-22 | 0.031181662 | 93.88437754 |
| rs2565661 | A | T | 0.9156 | -0.2921 | 0.0468 | 4.93E-10 | 0.013169817 | 38.92904429 |
| rs78689347 | C | A | 0.0149 | 0.698 | 0.1104 | 3.00E-10 | 0.013509262 | 39.94615958 |
| Table S4V. Instrument Variables for HLA DR on CD33dim HLA DR+ CD11b+ | | | | | | | | |
| SNP | Effect allele | Other allele | Eaf | Beta | SE | P value | R2 | F |
| rs61742525 | A | G | 0.0918 | 0.4117 | 0.06776 | 1.54E-09 | 0.022093286 | 36.87084082 |
| rs9269109 | C | T | 0.3574 | 0.3633 | 0.04076 | 1.28E-18 | 0.046365235 | 79.34700657 |
| rs6917212 | G | C | 0.7641 | -0.4813 | 0.04452 | 2.31E-26 | 0.066752308 | 116.7318892 |
| rs11074934 | T | C | 0.2194 | -0.3589 | 0.04157 | 1.36E-17 | 0.043627651 | 74.44833165 |

**Table S5. Mendelian Randomization and Sensitivity Analysis Results for the Association Between Caffeinated Beverage Consumption and AMD Subtypes.**

| Pval thershold of IV extraction | GWAS id of exposure | GWAS id of outcome | Outcome | Exposure trait | NSNP | MR Egger | | | Weighted median | | | Inverse variance weighted | | | Simple mode | | | Weighted mode | | | Cochran's Q test_pval | | egger intercept_pval | Samplesize of exposure | OR | I^2^_GX_ |
| --- | --- | --- | --- | --- | --- | --- | --- | --- | --- | --- | --- | --- | --- | --- | --- | --- | --- | --- | --- | --- | --- | --- | --- | --- | --- | --- |
|  |  |  |  |  |  | β | SE | Pval | β | SE | Pval | β | SE | Pval | β | SE | Pval | β | SE | Pval | MR Egger | Inverse variance weighted |  |  |  |  |
| 5.00E-06 | ukb-b-9508 | winkler_et_al_earlyamd_meta | early AMD | coffee consumed | 22 | 0.1599198 | 1.0311706 | 0.8783082 | 0.3953023 | 0.3973976 | 0.3198688 | 0.1088663 | 0.3087902 | 0.7244201 | 0.5629738 | 0.7785782 | 0.4776081 | 0.489454 | 0.5831304 | 0.4107234 | 0.06278613 | 0.08311446 | 0.9590293 | 64949 | 1.115013 | 0.758 |
| 5.00E-06 | ukb-b-9508 | finngen_R11_WET_AMD | dry AMD | coffee consumed | 22 | -0.157741 | 1.2337698 | 0.2157004 | 0.4308508 | 0.53476 | 0.4204216 | 0.3203189 | 0.4230337 | 0.4489332 | 1.911911 | 1.0851862 | 0.0926512 | 0.654268 | 1.1152933 | 0.5637041 | 0.07780446 | 0.04143395 | 0.4489332 | 64949 | 1.377567 | 0.758 |
| 5.00E-06 | ukb-b-9508 | finngen_R11_DRY_AMD | wet AMD | coffee consumed | 22 | -1.579723 | 1.0576341 | 0.1508797 | 0.2728301 | 0.4542115 | 0.5480614 | 0.2955225 | 0.3693893 | 0.4236935 | 0.3771648 | 0.8824831 | 0.6734454 | -0.11177 | 0.9227307 | 0.9047394 | 0.1175086 | 0.0514789 | 0.075005 | 64949 | 1.3438283 | 0.758 |
| 5.00E-06 | ukb-b-17988 | winkler_et_al_earlyamd_meta | early AMD | tea consumed | 17 | -0.904574 | 0.9078357 | 0.3359632 | 0.144964 | 0.5014638 | 0.7725189 | 0.223482 | 0.3959176 | 0.5724371 | 0.2052071 | 0.9809197 | 0.8371077 | 0.083601 | 0.9549064 | 0.9313933 | 0.2916588 | 0.2341854 | 0.1916832 | 64949 | 1.2504231 | 0.8289 |
| 5.00E-06 | ukb-b-17988 | finngen_R11_WET_AMD | dry AMD | tea consumed | 17 | 1.4531556 | 0.8535488 | 0.1092905 | 0.3597513 | 0.6129255 | 0.5572431 | -0.194852 | 0.4566684 | 0.6696113 | 0.3575215 | 1.0654966 | 0.7415739 | 0.684854 | 0.7540196 | 0.377213 | 0.5624754 | 0.2969237 | 0.0417361 | 64949 | 0.8229565 | 0.8289 |
| 5.00E-06 | ukb-b-17988 | finngen_R11_DRY_AMD | wet AMD | tea consumed | 17 | -0.47061 | 0.8196406 | 0.5743653 | -0.431945 | 0.5385117 | 0.4224896 | -0.811899 | 0.3987732 | 0.0417513 | -0.520177 | 0.723986 | 0.4828159 | -0.4442 | 0.6420753 | 0.4989664 | 0.2749098 | 0.321319 | 0.6378919 | 64949 | 0.4440143 | 0.8289 |

**Table S6.** **Inverse Mendelian Randomization and Sensitivity Analysis Results for the Association Between AMD Subtypes and** **Caffeinated Beverage Consumption.**

| Pval thershold of IV extraction | GWAS id of exposure | GWAS id of outcome | Outcome | Exposure trait | NSNP | MR Egger | | | Weighted median | | | Inverse variance weighted | | | Simple mode | | | Weighted mode | | | Cochran's Q test_pval | | egger intercept_pval | Samplesize of exposure | OR | I^2^_GX_ |
| --- | --- | --- | --- | --- | --- | --- | --- | --- | --- | --- | --- | --- | --- | --- | --- | --- | --- | --- | --- | --- | --- | --- | --- | --- | --- | --- |
|  |  |  |  |  |  | β | SE | Pval | β | SE | Pval | β | SE | Pval | β | SE | Pval | β | SE | Pval | MR Egger | Inverse variance weighted |  |  |  |  |
| 5.00E-08 | finngen_R11_DRY_AMD | ukb-b-17988 | tea consumed | dry AMD | 21 | 0.000557303 | 0.003892243 | 0.8876532 | 0.000281263 | 0.003025581 | 0.925934 | 0.000231782 | 0.002435822 | 0.9241913 | 0.003423159 | 0.005189247 | 0.5169946 | 0.000404702 | 0.002819102 | 0.8872868 | 0.9281168 | 0.9492248 | 0.9157337 | 306042 | 1.000232 | 0.985 |
| 5.00E-08 | finngen_R11_DRY_AMD | ukb-b-9508 | coffee consumed | dry AMD | 21 | -0.002063696 | 0.004918698 | 6.80E-01 | -0.001090182 | 0.003714408 | 7.69E-01 | -0.001302873 | 0.00300336 | 6.66E-01 | -0.001838248 | 0.006943885 | 7.94E-01 | -0.001228621 | 0.0032356 | 7.08E-01 | 0.2060359 | 0.251409 | 0.8449054 | 306042 | 0.998698 | 0.985 |
| 5.00E-08 | finngen_R11_WET_AMD | ukb-b-17988 | tea consumed | wet AMD | 27 | 0.000708123 | 0.003651675 | 8.48E-01 | 0.001526582 | 0.003022043 | 6.13E-01 | 0.002002781 | 0.002220153 | 3.67E-01 | 0.01233078 | 0.00566346 | 3.87E-02 | 0.001438592 | 0.002784567 | 6.10E-01 | 0.5291733 | 0.5742444 | 0.6590448 | 306075 | 1.002005 | 0.9799 |
| 5.00E-08 | finngen_R11_WET_AMD | ukb-b-9508 | coffee consumed | wet AMD | 27 | -0.003922448 | 0.00484698 | 0.4260031 | -6.50689E-05 | 0.003354441 | 0.9845237 | -0.000470937 | 0.002935786 | 0.8725561 | -0.002467716 | 0.007103473 | 0.7310905 | -0.001444464 | 0.003004139 | 0.6346628 | 0.0982179 | 0.09966433 | 0.3783266 | 306075 | 0.9995292 | 0.9799 |
| 5.00E-08 | winkler_et_al_earlyamd_meta | ukb-b-17988 | tea consumed | early AMD | 8 | 0.001003385 | 0.008674608 | 9.12E-01 | 0.000804951 | 0.005305492 | 8.79E-01 | 0.00054086 | 0.004565918 | 9.06E-01 | 0.010046627 | 0.008620728 | 2.82E-01 | 0.00122999 | 0.005008825 | 8.13E-01 | 0.2971321 | 0.4014234 | 0.9504366 | 105258 | 1.000541 | 0.9837 |
| 5.00E-08 | winkler_et_al_earlyamd_meta | ukb-b-9508 | coffee consumed | early AMD | 8 | 0.004238591 | 0.009241133 | 0.6626191 | -0.00221712 | 0.005891779 | 0.7066883 | -0.005565027 | 0.005494845 | 0.3111689 | -0.001710981 | 0.010347858 | 0.8733452 | -0.001479211 | 0.005849644 | 0.8076323 | 0.3745385 | 0.3119762 | 0.2447527 | 105258 | 0.9944504 | 0.9837 |

**Table S7. Mendelian Randomization and Sensitivity Analysis Results for the Association Between Immune Cell Types and Dry AMD (Including Geographic Atrophy).**

| Pval thershold of IV extraction | GWAS id of exposure | GWAS id of outcome | Outcome | Exposure trait | NSNP | MR Egger | | | Weighted median | | | Inverse variance weighted | | | Simple mode | | | Weighted mode | | | Cochran's Q test_pval | | egger intercept_pval | Samplesize of exposure | OR | I^2^_GX_ |
| --- | --- | --- | --- | --- | --- | --- | --- | --- | --- | --- | --- | --- | --- | --- | --- | --- | --- | --- | --- | --- | --- | --- | --- | --- | --- | --- |
|  |  |  |  |  |  | β | SE | Pval | β | SE | Pval | β | SE | Pval | β | SE | Pval | β | SE | Pval | MR Egger | Inverse variance weighted |  |  |  |  |
| 1.00E-08 | ebi-a-GCST90001485 | finngen_R11_DRY_AMD | dAMD | CD39+ resting Treg % CD4 Treg | 15 | -0.07018198 | 0.02227603 | 0.007663 | -0.04192484 | 0.02103582 | 0.046259 | -0.05383992 | 0.014833 | 0.000284 | -0.05240801 | 0.03755802 | 0.18464 | -0.04435933 | 0.01472871 | 0.009331 | 0.1409416 | 0.1355368 | 0.3429876 | 3757 | 0.9 | 0.9853 |
| 1.00E-08 | ebi-a-GCST90001493 | finngen_R11_DRY_AMD | dAMD | Secreting Treg % CD4 Treg | 5 | 0.08444049 | 0.02738421 | 5.40E-02 | 0.07357501 | 0.01897772 | 1.06E-04 | 0.0767405 | 0.01758 | 1.27E-05 | 0.04788608 | 0.03043862 | 1.91E-01 | 0.07343647 | 0.0195258 | 1.98E-02 | 0.011210092 | 0.006633886 | 0.2113696 | 3757 | 1.1 | 0.9819 |
| 1.00E-08 | ebi-a-GCST90001499 | finngen_R11_DRY_AMD | dAMD | Activated & resting Treg % CD4 Treg | 5 | -0.08246502 | 0.02675488 | 5.40E-02 | -0.07231176 | 0.019082846 | 1.45E-04 | -0.07537138 | 0.01722 | 1.20E-05 | -0.0475339 | 0.03024667 | 1.91E-01 | -0.07136052 | 0.01947943 | 2.15E-02 | 0.2430545 | 0.356674 | 0.7257682 | 3757 | 0.9 | 0.9823 |
| 5.00E-06 | ebi-a-GCST90001512 | finngen_R11_DRY_AMD | dAMD | CD25hi CD45RA- CD4 not Treg %T cell | 14 | 0.0557005 | 0.02338973 | 0.034674 | 0.02124136 | 0.02014842 | 0.291771 | 0.0356522 | 0.019238 | 0.063903 | -0.02532401 | 0.04675683 | 0.597247 | 0.02106824 | 0.01993391 | 0.309801 | 0.05323795 | 0.02893954 | 0.1845867 | 3757 | 1 | 0.9221 |
| 1.00E-08 | ebi-a-GCST90001535 | finngen_R11_DRY_AMD | dAMD | CD45RA- CD4+ %CD4+ | 3 | 0.1373725 | 0.04101819 | 1.85E-01 | 0.1323447 | 0.03400644 | 9.95E-05 | 0.1322046 | 0.026891 | 8.82E-07 | 0.1309133 | 0.0418452 | 8.88E-02 | 0.1330963 | 0.03585143 | 6.55E-02 | 0.8752373 | 0.9740966 | 0.894752 | 3757 | 1.1 | 0.9727 |
| 5.00E-06 | ebi-a-GCST90001543 | finngen_R11_DRY_AMD | dAMD | EM CD4+ %CD4+ | 16 | 0.07205391 | 0.04109509 | 0.1014 | 0.04140545 | 0.0363889 | 0.255179 | 0.07884589 | 0.030629 | 0.010046 | 0.15017423 | 0.06663035 | 0.039595 | 0.04900697 | 0.0292915 | 0.115033 | 0.001063746 | 0.001700863 | 0.7995146 | 3757 | 1.1 | 0.8753 |
| 1.00E-08 | ebi-a-GCST90001547 | finngen_R11_DRY_AMD | dAMD | TD CD4+ %T cell | 5 | -0.1401995 | 0.09298568 | 0.228731 | -0.1514848 | 0.04733664 | 0.001374 | -0.1812931 | 0.047645 | 0.000142 | -0.1424101 | 0.06353382 | 0.088476 | -0.1459455 | 0.0440801 | 0.029629 | 0.0831324 | 0.1203885 | 0.6293216 | 3757 | 0.8 | 0.8734 |
| 1.00E-08 | ebi-a-GCST90001670 | finngen_R11_DRY_AMD | dAMD | CD39+ CD8br %T cell | 6 | 0.045615169 | 0.06936326 | 0.546697 | 0.004341413 | 0.04438566 | 0.922082 | -0.02034857 | 0.034298 | 0.552989 | -0.072851806 | 0.0731027 | 0.364728 | 0.027712202 | 0.05033134 | 0.605606 | 0.8386702 | 0.756993 | 0.3353747 | 3757 | 1 | 0.9698 |
| 1.00E-08 | ebi-a-GCST90001671 | finngen_R11_DRY_AMD | dAMD | CD39+ CD8br %CD8br | 6 | 0.042418222 | 0.07014989 | 0.578016 | -0.008228215 | 0.04424134 | 0.852457 | -0.02223832 | 0.034542 | 0.519698 | -0.069318077 | 0.06818546 | 0.35598 | -0.20296157 | 0.02784365 | 0.048946 | 0.5940477 | 0.8226246 | 0.7546629 | 3757 | 1 | 0.9711 |
| 5.00E-06 | ebi-a-GCST90001691 | finngen_R11_DRY_AMD | dAMD | CD28+ CD45RA- CD8br %T cell | 17 | 0.014620771 | 0.01435044 | 0.324432 | 0.001548938 | 0.01241021 | 0.900673 | 0.00934938 | 0.012559 | 0.456603 | 0.012744063 | 0.04275864 | 0.769503 | 0.004109599 | 0.01153392 | 0.726267 | 0.05491645 | 0.05881114 | 0.4413757 | 3757 | 1 | 0.9149 |
| 1.00E-08 | ebi-a-GCST90001735 | finngen_R11_DRY_AMD | dAMD | IgD- CD38dim | 3 | -0.08885282 | 0.05141952 | 0.33398 | -0.07718808 | 0.0365059 | 0.034481 | -0.07322813 | 0.033376 | 0.028234 | -0.0540674 | 0.06721466 | 0.505587 | -0.08051657 | 0.03456424 | 0.145194 | 0.2202248 | 0.3903846 | 0.7039493 | 3757 | 0.9 | 0.9636 |
| 1.00E-08 | ebi-a-GCST90001828 | finngen_R11_DRY_AMD | dAMD | IgD on transitional | 4 | 0.047769397 | 0.0809273 | 0.614818 | 0.000139633 | 0.03175741 | 0.996492 | -0.01414628 | 0.031568 | 0.65407 | 0.007377286 | 0.06148953 | 0.912086 | 0.000362015 | 0.0331275 | 0.991967 | 0.2671434 | 0.3118604 | 0.4896904 | 3757 | 1 | 0.967 |
| 1.00E-08 | ebi-a-GCST90001984 | finngen_R11_DRY_AMD | dAMD | HLA DR on CD14- CD16+ monocyte | 7 | 0.29022391 | 0.1325564 | 8.02E-02 | 0.13947445 | 0.03090338 | 6.38E-06 | 0.10002038 | 0.038078 | 8.62E-03 | 0.08615211 | 0.04020241 | 7.58E-02 | 0.12394159 | 0.02962961 | 5.79E-03 | 0.065867545 | 0.0518528 | 0.01470018 | 3757 | 1.1 | 0.8942 |
| 1.00E-08 | ebi-a-GCST90002007 | finngen_R11_DRY_AMD | dAMD | HLA DR on CD14+ CD16+ monocyte | 6 | 0.3282707 | 0.20832822 | 1.90E-01 | 0.1270576 | 0.02884977 | 1.06E-05 | 0.1016301 | 0.053606 | 5.80E-02 | 0.1310086 | 0.03875685 | 1.97E-02 | 0.1257623 | 0.030321 | 8.93E-03 | 0.000996617 | 0.000189475 | 0.3239718 | 3757 | 1.1 | 0.9024 |
| 1.00E-08 | ebi-a-GCST90002099 | finngen_R11_DRY_AMD | dAMD | CD4RA on TD CD4+ | 3 | -0.11567775 | 0.0476157 | 2.49E-01 | -0.09023039 | 0.0318646 | 4.63E-03 | -0.10184323 | 0.025662 | 7.23E-05 | -0.04066717 | 0.055333136 | 5.39E-01 | -0.12263548 | 0.03362405 | 6.76E-02 | 0.1711006 | 0.3348906 | 0.7523882 | 3757 | 0.9 | 0.9704 |
| 1.00E-08 | ebi-a-GCST90002102 | finngen_R11_DRY_AMD | dAMD | CD45RA on resting Treg | 7 | -0.04366754 | 0.04127861 | 0.338521 | -0.07352293 | 0.02595824 | 0.004621 | -0.05685441 | 0.026679 | 0.033088 | -0.04755736 | 0.06033834 | 0.460589 | -0.08849872 | 0.02459884 | 0.011398 | 0.01207535 | 0.01872217 | 0.6756113 | 3757 | 0.9 | 0.9631 |
| 1.00E-08 | ebi-a-GCST90002110 | finngen_R11_DRY_AMD | dAMD | HLA DR on CD33dim HLA DR+ CD11b+ | 4 | 0.2989658 | 0.33861275 | 4.70E-01 | 0.1396708 | 0.03418944 | 4.40E-05 | 0.117374 | 0.041401 | 4.58E-03 | 0.1329919 | 0.04043186 | 4.61E-02 | 0.1349441 | 0.03292838 | 2.63E-02 | 0.0417929 | 0.06344936 | 0.6423587 | 3757 | 1.1 | 0.6319 |

**Table S8. Mendelian Randomization and Sensitivity Analysis Results for the Association Between Tea Consumption and Immune Cell Types.**

| Pval thershold of IV extraction | GWAS id of exposure | GWAS id of outcome | Outcome trait | Exposure trait | NSNP | MR Egger | | | Weighted median | | | Inverse variance weighted | | | Simple mode | | | Weighted mode | | | Cochran's Q test_pval | | egger intercept_pval | Samplesize of exposure | OR | I^2^_GX_ |
| --- | --- | --- | --- | --- | --- | --- | --- | --- | --- | --- | --- | --- | --- | --- | --- | --- | --- | --- | --- | --- | --- | --- | --- | --- | --- | --- |
|  |  |  |  |  |  | β | SE | Pval | β | SE | Pval | β | SE | Pval | β | SE | Pval | β | SE | Pval | MR Egger | Inverse variance weighted |  |  |  |  |
| 5.00E-06 | ukb-b-17988 | GCST90001485 | CD39+ resting Treg % CD4 Treg | tea consumed | 17 | 1.7020165 | 1.3249841 | 0.2184318 | 1.0826675 | 0.7443534 | 0.14580914 | 0.5481434 | 0.5964511 | 0.3580914 | 1.2303651 | 1.2731578 | 0.348231 | 1.3225415 | 1.0013357 | 0.2051561 | 0.1529379 | 0.1490872 | 0.3447184 | 64949 | 1.730038 | 0.8289 |
| 5.00E-06 | ukb-b-17988 | GCST90001493 | Secreting Treg % CD4 Treg | tea consumed | 17 | -1.5513835 | 1.1270143 | 0.18885483 | -1.0000242 | 0.7331104 | 0.17254111 | -1.122735 | 0.4948602 | 0.02328095 | -0.9458181 | 1.1004051 | 0.40274959 | -0.8880454 | 0.8612662 | 0.31783258 | 0.3899282 | 0.4477834 | 0.6760975 | 64949 | 0.3253886 | 0.8289 |
| 5.00E-06 | ukb-b-17988 | GCST90001499 | Activated & resting Treg % CD4 Treg | tea consumed | 17 | 1.5585293 | 1.1010096 | 0.17733573 | 0.974002 | 0.7470138 | 0.19228107 | 1.1264108 | 0.4962948 | 0.02322941 | 0.9453533 | 1.0263254 | 0.37067627 | 0.8884284 | 0.8596242 | 0.31673592 | 0.470393 | 0.5296185 | 0.6664398 | 64949 | 3.084565 | 0.8289 |
| 5.00E-06 | ukb-b-17988 | GCST90001512 | CD25hi CD45RA- CD4 not Treg %T cell | tea consumed | 17 | -1.6494406 | 1.5053227 | 0.29047472 | -1.296035 | 0.7736149 | 0.09387591 | -0.9467351 | 0.6628503 | 0.15321162 | -1.2822357 | 1.1323092 | 0.27414893 | -1.4035171 | 0.8999192 | 0.13841254 | 0.0481724 | 0.06004284 | 0.6086581 | 64949 | 0.3880058 | 0.8289 |
| 5.00E-06 | ukb-b-17988 | GCST90001535 | CD45RA- CD4+ %CD4+ | tea consumed | 17 | -2.171628 | 1.0822759 | 0.06317503 | -1.830962 | 0.7114114 | 0.01006163 | -1.146708 | 0.4879097 | 0.01876097 | -2.04209 | 1.2671891 | 0.12661652 | -2.06876 | 0.9928327 | 0.05358139 | 0.5605169 | 0.5491145 | 0.3055055 | 64949 | 0.3176808 | 0.8289 |
| 5.00E-06 | ukb-b-17988 | GCST90001543 | EM CD4+ %CD4+ | tea consumed | 17 | -1.8454202 | 1.0971647 | 0.1132658 | -0.8732714 | 0.7093124 | 0.218266 | -0.6507623 | 0.4946011 | 0.1882641 | -0.464627 | 1.1866947 | 0.7005705 | -0.7928161 | 1.0204962 | 0.4485563 | 0.5821761 | 0.5431213 | 0.2413589 | 64949 | 0.521648 | 0.8289 |
| 5.00E-06 | ukb-b-17988 | GCST90001547 | TD CD4+ %T cell | tea consumed | 17 | 2.5163448 | 1.1262489 | 0.04111038 | 0.7297032 | 0.7188309 | 0.31004623 | 1.0346496 | 0.5075006 | 0.0414784 | -0.173174 | 1.3119161 | 0.89662937 | -0.2075283 | 1.2807033 | 0.87330116 | 0.6001199 | 0.5100426 | 0.1612375 | 64949 | 2.8141199 | 0.8289 |
| 5.00E-06 | ukb-b-17988 | GCST90001670 | CD39+ CD8br %T cell | tea consumed | 17 | 1.15095544 | 1.1630362 | 0.3380592 | -0.40292837 | 0.729092 | 0.5805071 | 0.07617957 | 0.5242895 | 0.8844735 | -0.785134423 | 1.2538818 | 0.540039 | -1.05054075 | 1.2688699 | 0.4198863 | 0.6428762 | 0.6325323 | 0.3169452 | 64949 | 1.0791563 | 0.8289 |
| 5.00E-06 | ukb-b-17988 | GCST90001671 | CD39+ CD8br %CD8br | tea consumed | 17 | 1.22956734 | 1.1648846 | 0.3078894 | 0.01173508 | 0.7192449 | 0.9869824 | 0.18466569 | 0.5251014 | 0.7250811 | -1.0256114 | 1.2150776 | 0.4110729 | -0.83504273 | 1.1577234 | 0.481145 | 0.8368038 | 0.8256811 | 0.3308871 | 64949 | 1.2028163 | 0.8289 |
| 5.00E-06 | ukb-b-17988 | GCST90001691 | CD28+ CD45RA- CD8br %T cell | tea consumed | 17 | -0.05916762 | 1.2410454 | 0.9626037 | -0.41772701 | 0.7425012 | 0.5737111 | -0.23576886 | 0.5420269 | 0.6635796 | -0.67835649 | 1.0767515 | 0.5375867 | -0.50317914 | 0.94126 | 0.6002906 | 0.3146969 | 0.3792242 | 0.8754739 | 64949 | 0.7899632 | 0.8289 |
| 5.00E-06 | ukb-b-17988 | GCST90001735 | IgD- CD38dim | tea consumed | 17 | 0.6562587 | 1.2018275 | 0.5930604 | 0.4228117 | 0.7208889 | 0.5575299 | 0.3197764 | 0.7208889 | 0.5415843 | 0.5155197 | 1.0585358 | 0.6328544 | 0.4848698 | 0.8631135 | 0.5820584 | 0.3178066 | 0.3772933 | 0.7583771 | 64949 | 1.37682 | 0.8289 |
| 5.00E-06 | ukb-b-17988 | GCST90001828 | IgD on transitional | tea consumed | 17 | 0.52073221 | 1.1479631 | 0.6566002 | -0.05361041 | 0.7154157 | 0.9402656 | 0.39854065 | 0.5130804 | 0.4373 | -0.3766723 | 1.1671435 | 0.7510787 | -0.13750271 | 0.9242797 | 0.883596 | 0.4428661 | 0.5148839 | 0.9067716 | 64949 | 1.4896492 | 0.8289 |
| 5.00E-06 | ukb-b-17988 | GCST90001984 | HLA DR on CD14- CD16+ monocyte | tea consumed | 17 | 0.9904754 | 1.3134792 | 0.4624725 | 0.1958749 | 0.7551328 | 0.7953333 | 0.2184309 | 0.5788411 | 0.7059068 | -0.3381606 | 1.257323 | 0.7914033 | 0.1370261 | 0.9936838 | 0.8920422 | 0.1736051 | 0.1972045 | 0.5206845 | 64949 | 1.2441231 | 0.8289 |
| 5.00E-06 | ukb-b-17988 | GCST90002007 | HLA DR on CD14+ CD16+ monocyte | tea consumed | 17 | 0.567978 | 1.1504267 | 0.6286612 | 0.6501822 | 0.7051768 | 0.3565218 | 0.7613564 | 0.516312 | 0.1403187 | 0.3952282 | 1.1847499 | 0.7430162 | 0.6409396 | 1.032143 | 0.543359 | 0.5231806 | 0.593769 | 0.8533188 | 64949 | 2.141179 | 0.8289 |
| 5.00E-06 | ukb-b-17988 | GCST90002099 | CD4RA on TD CD4+ | tea consumed | 17 | -0.04436539 | 1.2274772 | 0.9716445 | -0.21959922 | 0.763452 | 0.7738798 | -0.10473601 | 0.5544823 | 0.8501792 | 0.49102578 | 1.2092354 | 0.6900753 | -0.0424173 | 1.0848559 | 0.9692948 | 0.95039 | 0.9681071 | 0.9567642 | 64949 | 0.9005622 | 0.8289 |
| 5.00E-06 | ukb-b-17988 | GCST90002102 | CD45RA on resting Treg | tea consumed | 17 | 1.6412163 | 1.168027 | 0.1803575 | 0.8740241 | 0.736514 | 0.2353444 | 0.5776369 | 0.527701 | 0.2736792 | 0.8138204 | 1.281738 | 0.5344459 | 0.8410271 | 1.079791 | 0.4474179 | 0.7722285 | 0.7602568 | 0.3235851 | 64949 | 1.781823 | 0.8289 |
| 5.00E-06 | ukb-b-17989 | GCST90002110 | HLA DR on CD33dim HLA DR+ CD11b+ | tea consumed | 17 | 0.4146672 | 1.650475 | 0.80503699 | 1.3358251 | 0.9912683 | 0.17778966 | 1.3330591 | 0.7423263 | 0.07252858 | 0.7607944 | 1.4516668 | 0.60740497 | 1.3085967 | 1.3629694 | 0.35128674 | 0.97971 | 0.9833033 | 0.5426338 | 64949 | 3.792628 | 0.8289 |

**Figure S1. Forest Plot of the Reverse Mendelian Randomization Analysis Assessing the Causal Effects of AMD Subtypes on Caffeinated Beverage Consumption.
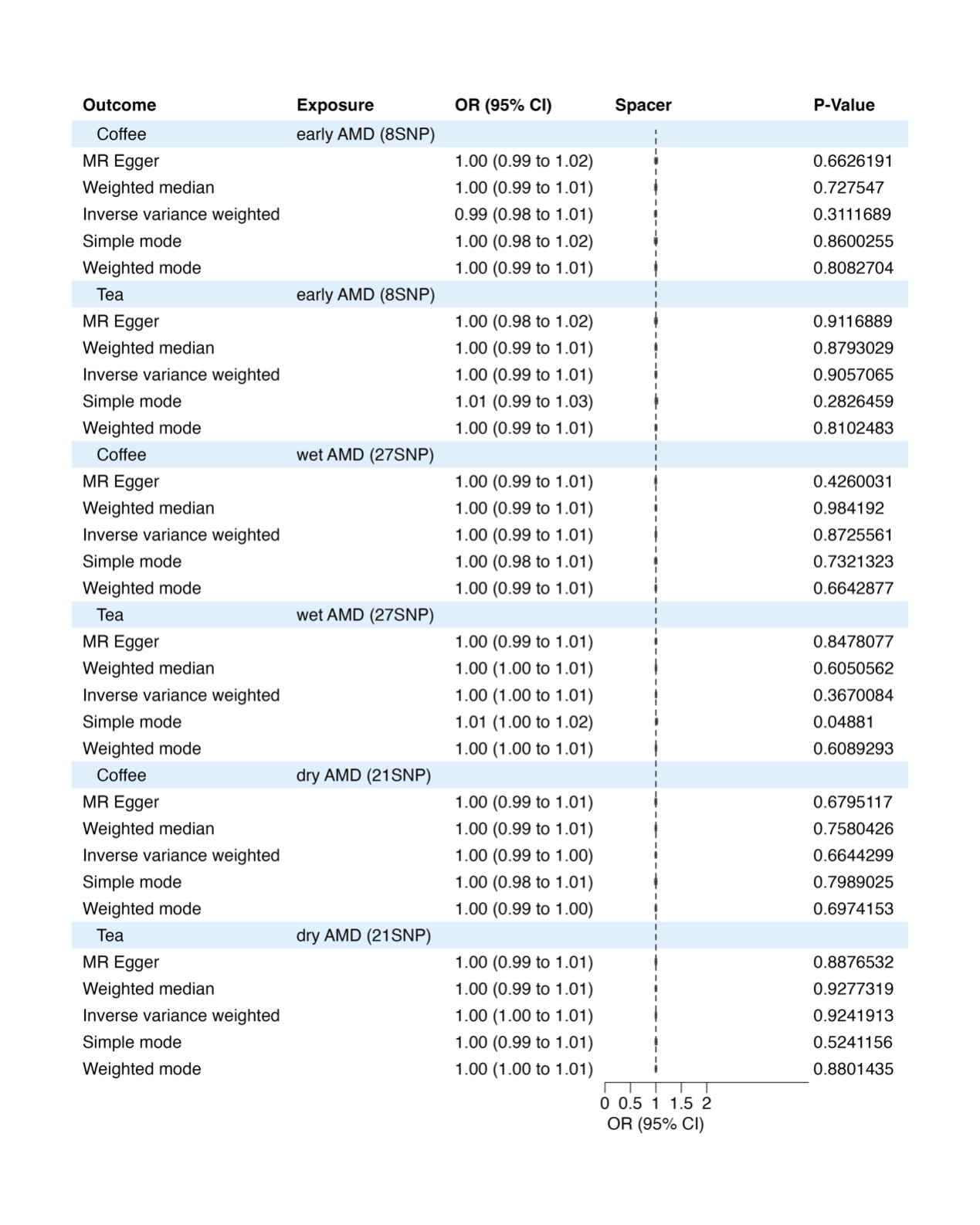
**

**Figure S2. Scatter Plots of the Reverse Mendelian Randomization Analysis Assessing the Causal Effects of AMD Subtypes on Caffeinated Beverage Consumption.**

**
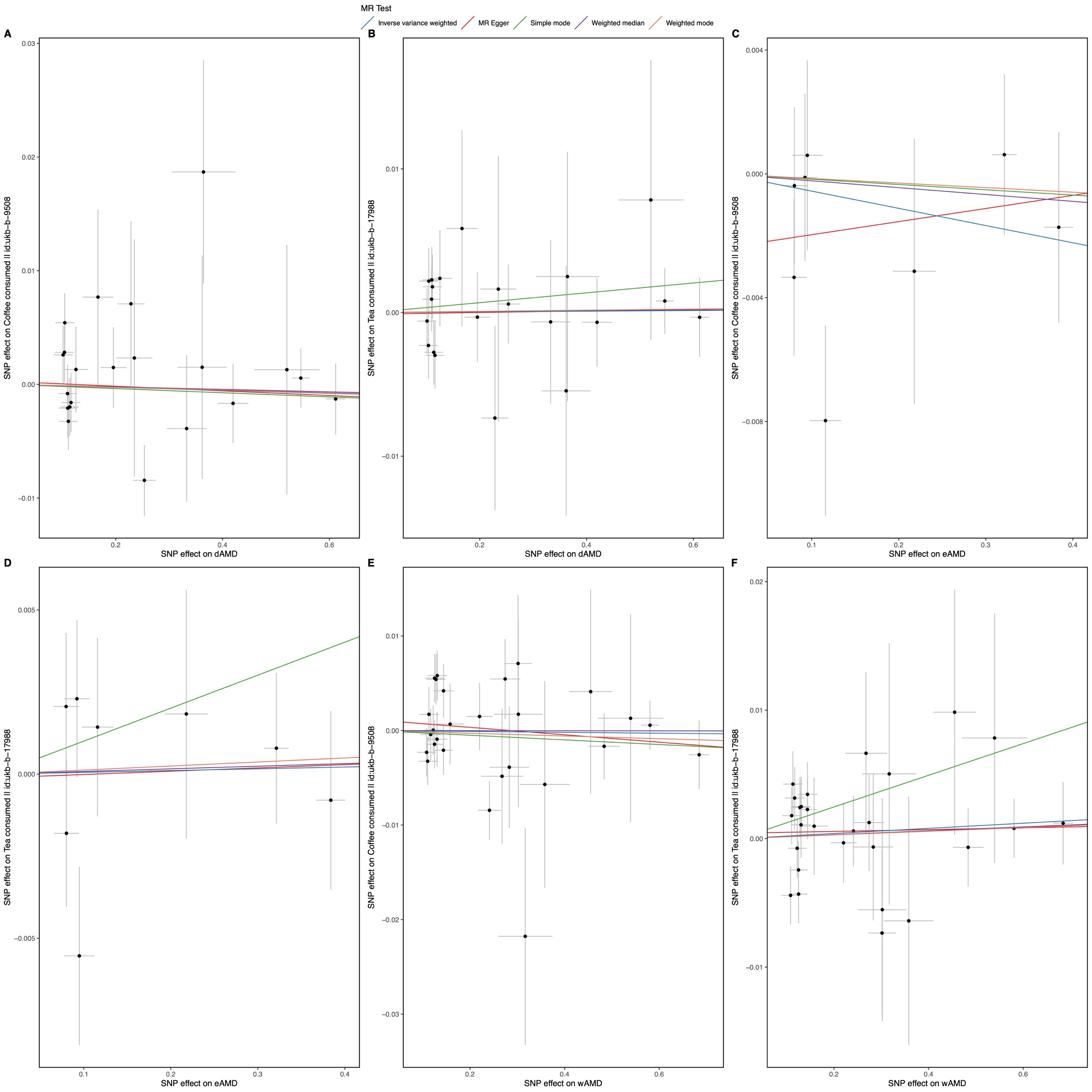
**

*Notes:* A–B. Associations of dry AMD (including geographic atrophy) with coffee consumption (A) and tea consumption (B). C–D. Associations of early AMD with coffee consumption (C) and tea consumption (D). E–F. Associations of wet AMD with coffee consumption (E) and tea consumption (F).

**Figure S3. Scatter Plot of the Association Between 17 Immune Cell Types and Dry AMD (Including Geographic Atrophy).
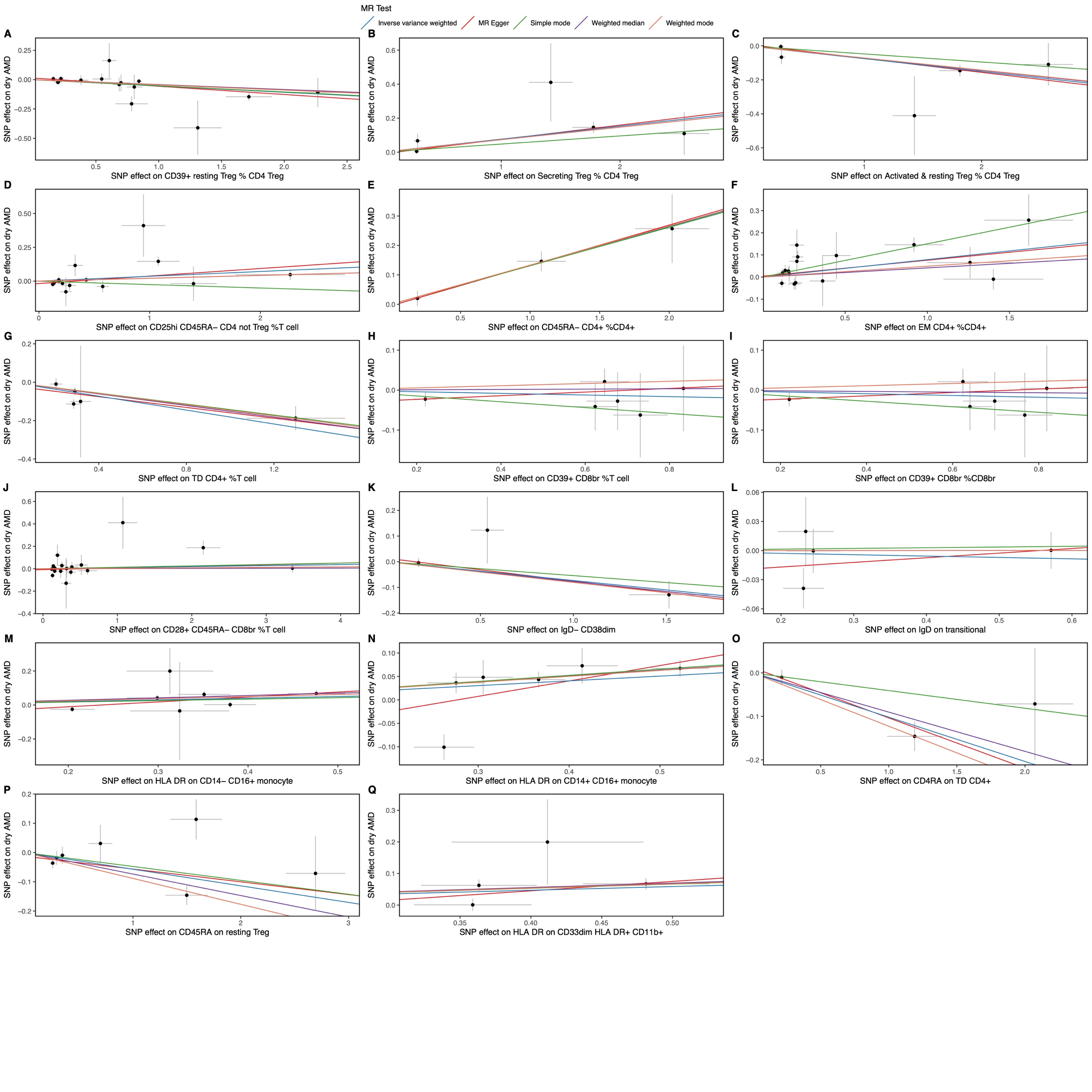
**

*Notes:* Panels A–Q correspond to the 17 immune cell types assessed in the second step of the two-step mediation MR framework. A.CD39+ resting Treg % CD4 Treg; B. Secreting Treg % CD4 Treg; C. Activated & resting Treg % CD4 Treg; D. CD25 hi CD45RA- CD4 not Treg % T cell; E. CD 45 RA- CD4+ % CD4+; F. EM CD4+ % CD4+; G. TD CD4+ % T cell; H. CD39+ CD8br % T cell; I. CD39+ CD8br % CD8 br; J. CD28+ CD45RA-CD8br % T cell; K. IgD- CD38 dim; L. IgD on transitional; M. HLA DR on CD14- CD16+ monocyte; N. HLA DR on CD14+ CD16+ monocyte; O. CD4RA on TD CD4+; P. CD45RA on resting Treg; Q. HLA DR on CD33 dim HLA DR+ CD11b+.

**Figure S4. Scatter Plots Showing the Associations Between Tea Consumption and 17 Immune Cell Types.**

**
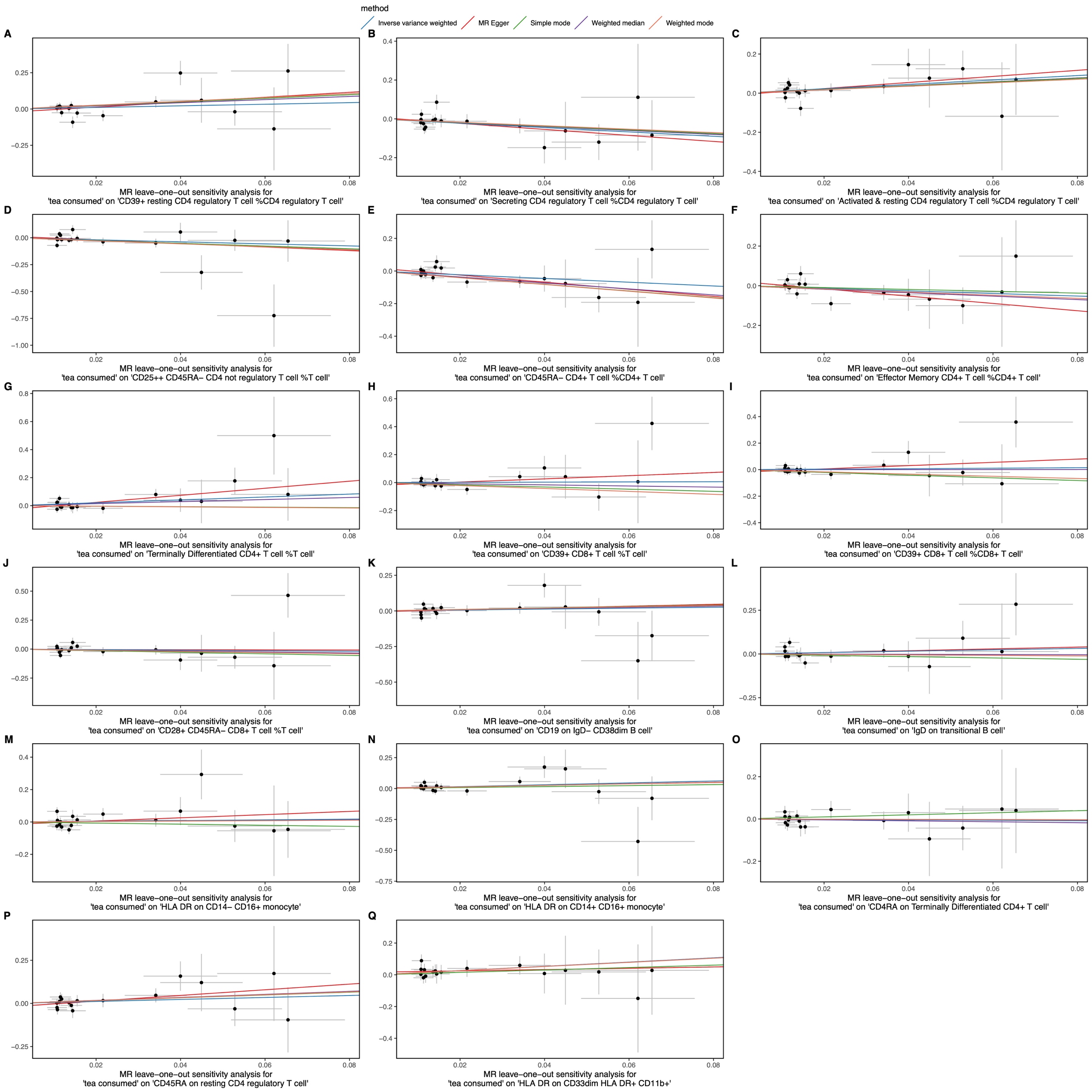
**

*Notes:* Panels A–Q correspond to the same 17 immune cell types shown in Figure S3, assessed here as outcomes in the first step of the two-step MR framework. Each plot presents the estimated association between tea consumption and a given immune cell type. A.CD39+ resting Treg % CD4 Treg; B. Secreting Treg % CD4 Treg; C. Activated & resting Treg % CD4 Treg; D. CD25 hi CD45RA- CD4 not Treg % T cell; E. CD 45 RA- CD4+ % CD4+; F. EM CD4+ % CD4+; G. TD CD4+ % T cell; H. CD39+ CD8br % T cell; I. CD39+ CD8br % CD8 br; J. CD28+ CD45RA-CD8br % T cell; K. IgD- CD38 dim; L. IgD on transitional; M. HLA DR on CD14- CD16+ monocyte; N. HLA DR on CD14+ CD16+ monocyte; O. CD4RA on TD CD4+; P. CD45RA on resting Treg; Q. HLA DR on CD33 dim HLA DR+ CD11b+.

**Figure S5. Leave-One-Out Analysis of Caffeinated Beverage Consumption on AMD Subtypes by SNP Exclusion.**


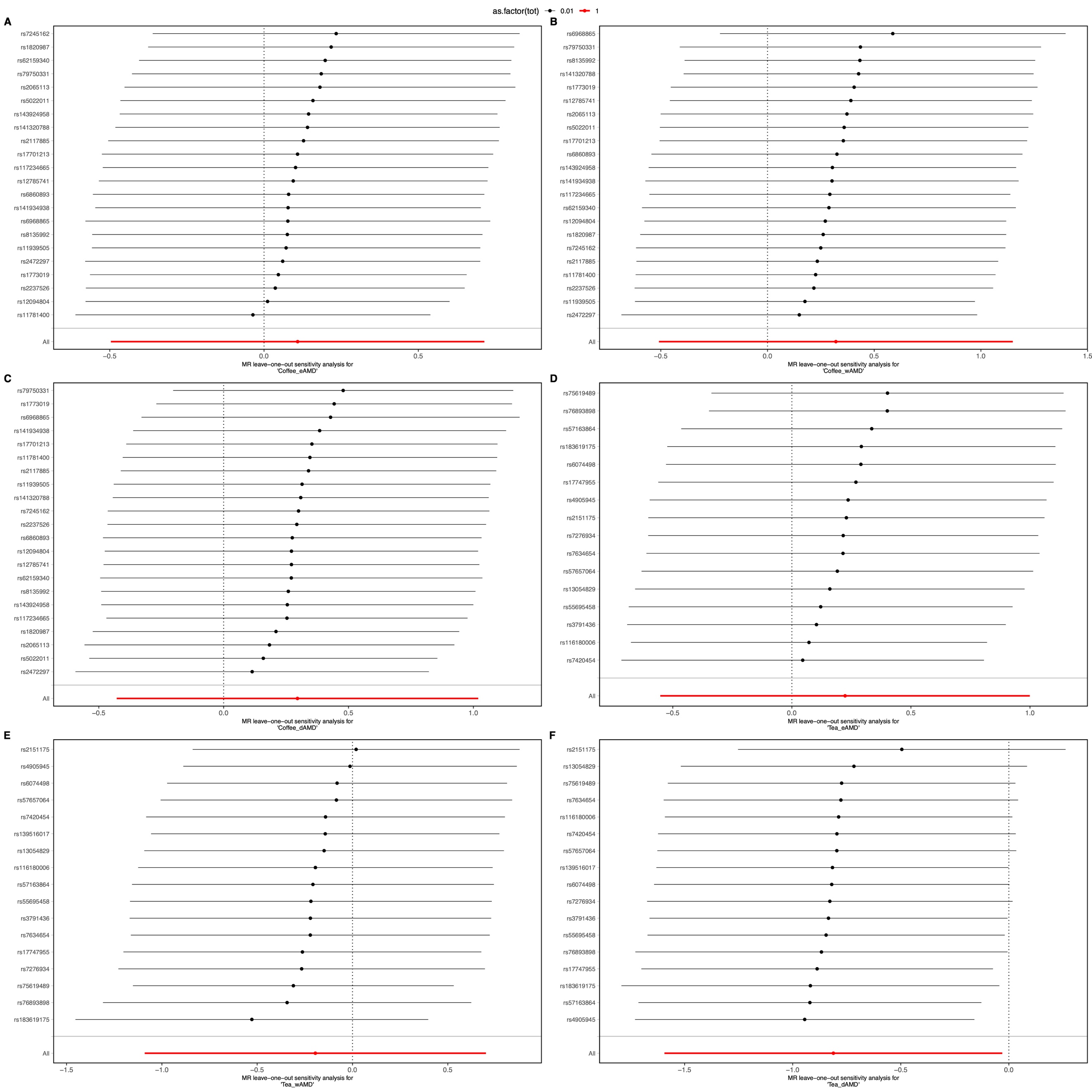


*Notes:* Each plot represents the leave-one-out (LOO) analysis for one exposure–outcome pair. The effect estimate was recalculated by iteratively excluding a single SNP at a time to assess the influence of individual variants on the overall causal estimate. No single SNP disproportionately altered the results, indicating stability of the causal associations. Panels A–C: Coffee consumption on early AMD (A), wet AMD (B), and dry AMD (including geographic atrophy) (C). Panels D–F: Tea consumption on early AMD (D), wet AMD (E), and dry AMD (including geographic atrophy) (F).

**Figure S6. Leave-One-Out Analysis of AMD Subtypes on Caffeinated Beverage Consumption by SNP Exclusion.**


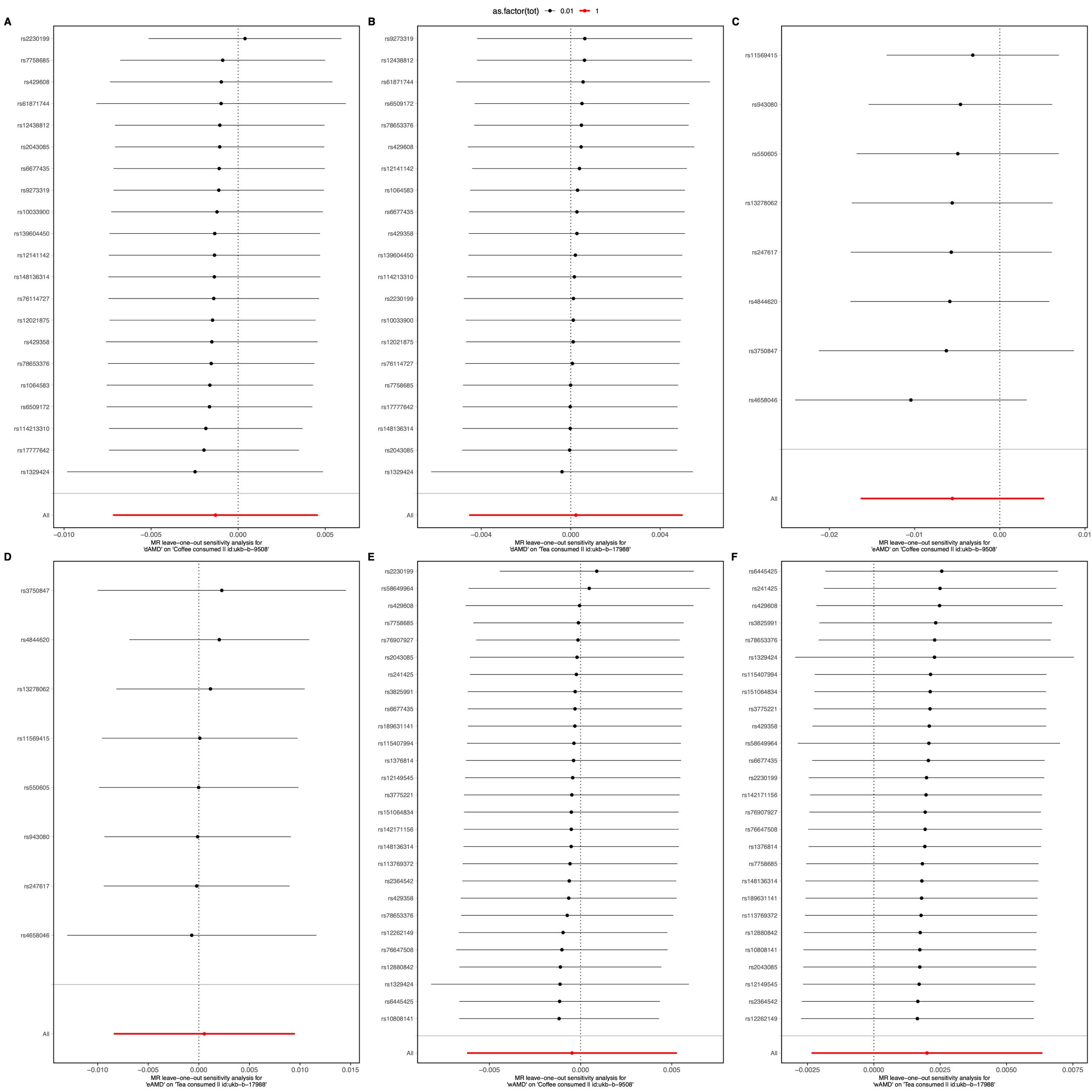


*Notes:* Panels A–B: Dry AMD (including geographic atrophy) on coffee consumption (A) and tea consumption (B). Panels C–D: Early AMD on coffee consumption (C) and tea consumption (D). Panels E–F: Wet AMD on coffee consumption (E) and tea consumption (F).

**Figure S7. Leave-One-Out Analysis of Immune Cell Type SNPs on Dry AMD (Including Geographic Atrophy) by SNP Exclusion.**


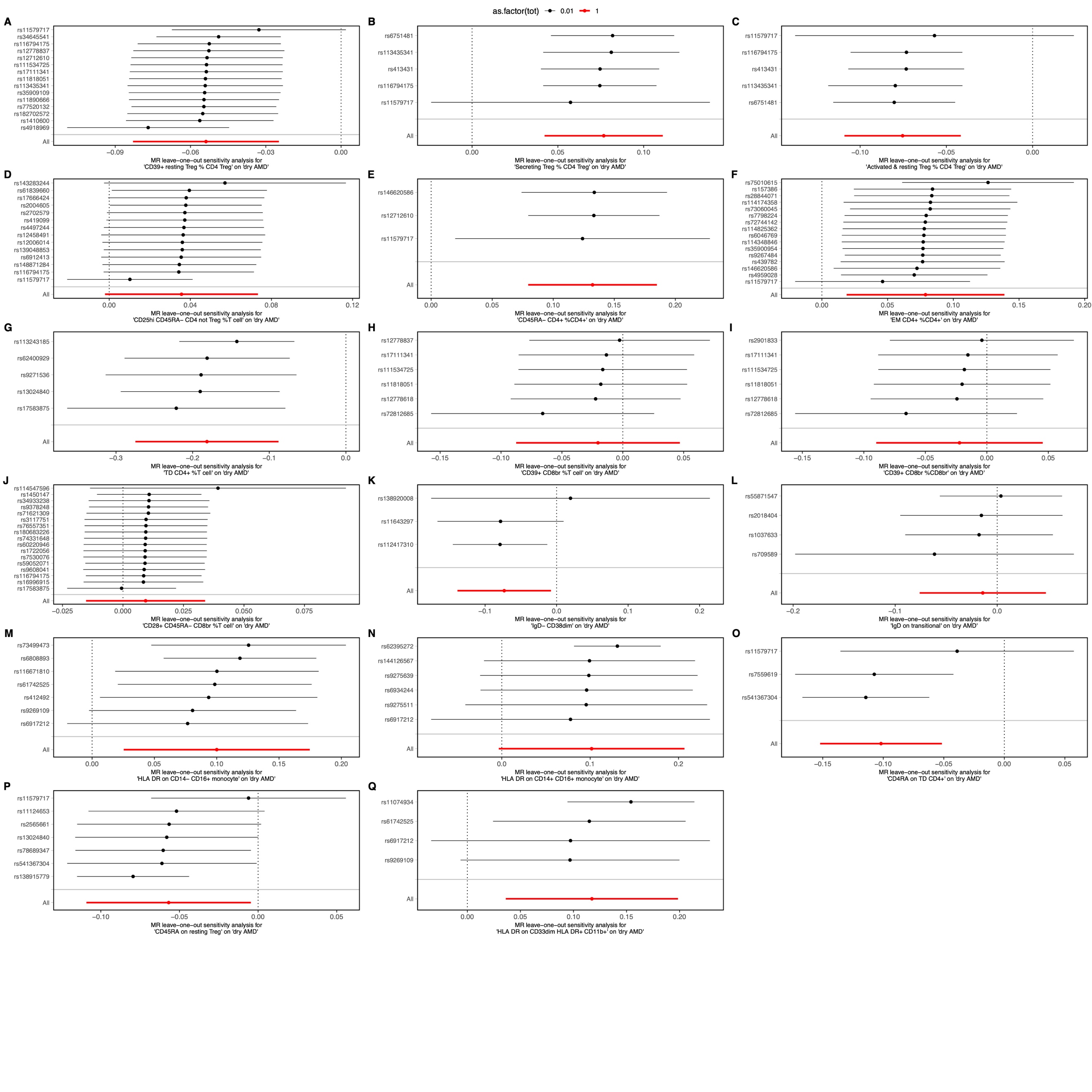


*Notes:* Panels A–Q: Leave-one-out analysis of 17 immune cell types on dry AMD (including geographic atrophy), corresponding to the same order as in Figures S3 and S4. A.CD39+ resting Treg % CD4 Treg; B. Secreting Treg % CD4 Treg; C. Activated & resting Treg % CD4 Treg; D. CD25 hi CD45RA- CD4 not Treg % T cell; E. CD 45 RA- CD4+ % CD4+; F. EM CD4+ % CD4+; G. TD CD4+ % T cell; H. CD39+ CD8br % T cell; I. CD39+ CD8br % CD8 br; J. CD28+ CD45RA-CD8br % T cell; K. IgD- CD38 dim; L. IgD on transitional; M. HLA DR on CD14- CD16+ monocyte; N. HLA DR on CD14+ CD16+ monocyte; O. CD4RA on TD CD4+; P. CD45RA on resting Treg; Q. HLA DR on CD33 dim HLA DR+ CD11b+.

**Figure S8. Leave-One-Out Analysis of Tea Consumption on Immune Cell Types by SNP Exclusion.**


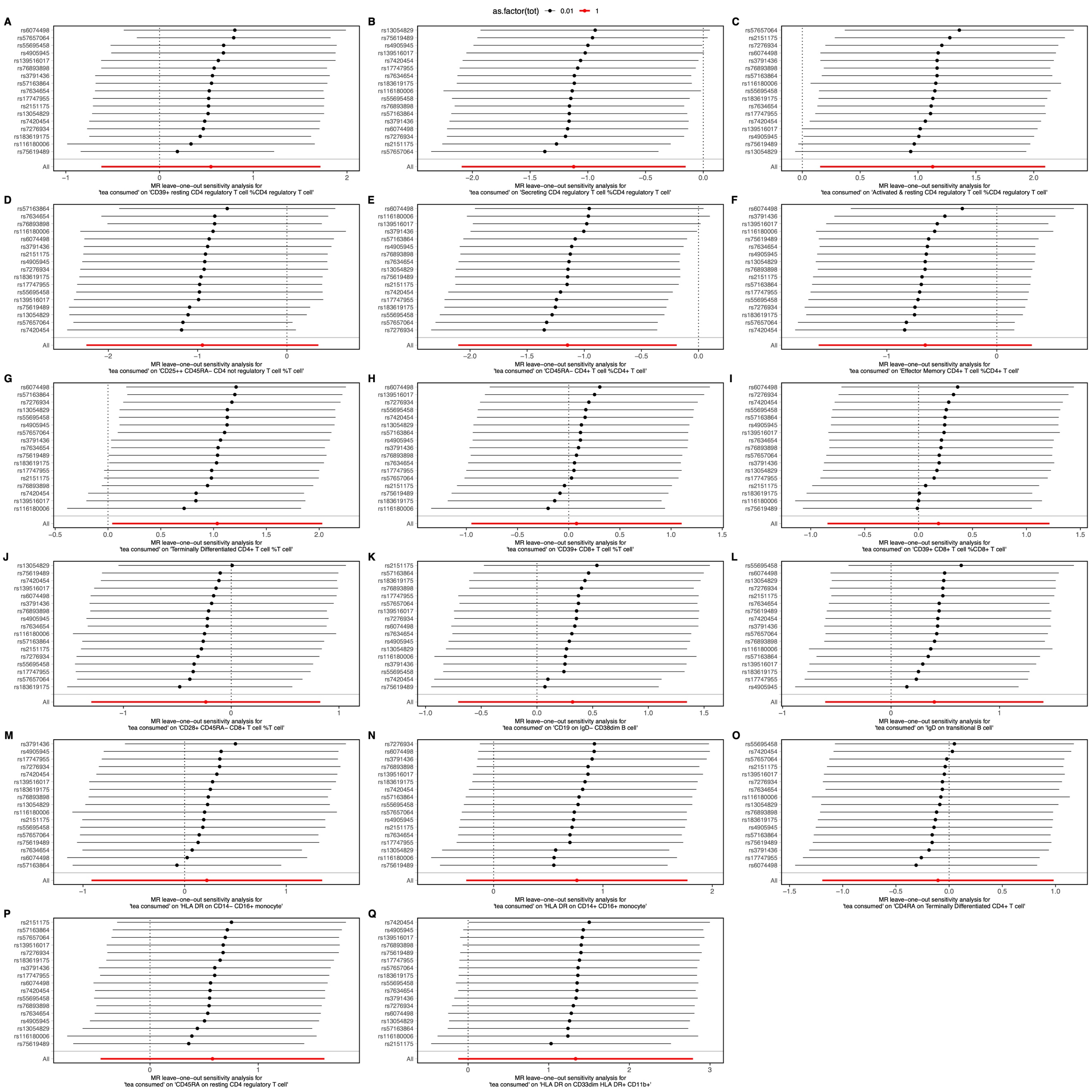


*Notes:* Panels A–Q: Leave-one-out analysis of tea consumption on 17 immune cell types. A.CD39+ resting Treg % CD4 Treg; B. Secreting Treg % CD4 Treg; C. Activated & resting Treg % CD4 Treg; D. CD25 hi CD45RA- CD4 not Treg % T cell; E. CD 45 RA- CD4+ % CD4+; F. EM CD4+ % CD4+; G. TD CD4+ % T cell; H. CD39+ CD8br % T cell; I. CD39+ CD8br % CD8 br; J. CD28+ CD45RA-CD8br % T cell; K. IgD- CD38 dim; L. IgD on transitional; M. HLA DR on CD14- CD16+ monocyte; N. HLA DR on CD14+ CD16+ monocyte; O. CD4RA on TD CD4+; P. CD45RA on resting Treg; Q. HLA DR on CD33 dim HLA DR+ CD11b+.

**Figure S9. Funnel Plot for Mendelian Randomization of Caffeinated Beverage Consumption and AMD Subtypes.**


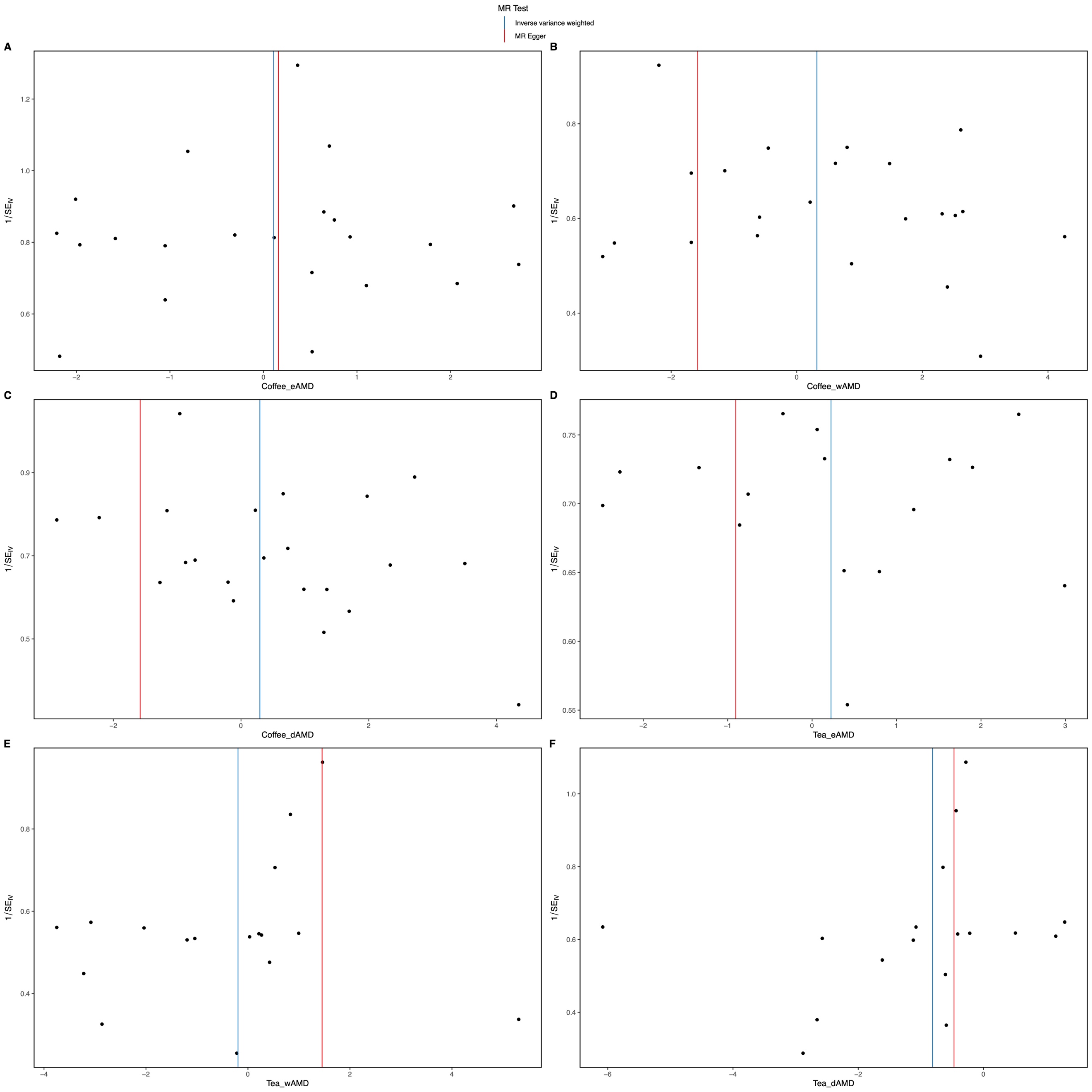


*Notes:* Panels A–C: Coffee consumption on early AMD (A), wet AMD (B), and dry AMD (including geographic atrophy) (C). Panels D–F: Tea consumption on early AMD (D), wet AMD (E), and dry AMD (including geographic atrophy) (F).

**Figure S10. Funnel Plot for Mendelian Randomization of AMD Subtypes and Caffeinated Beverage Consumption.**


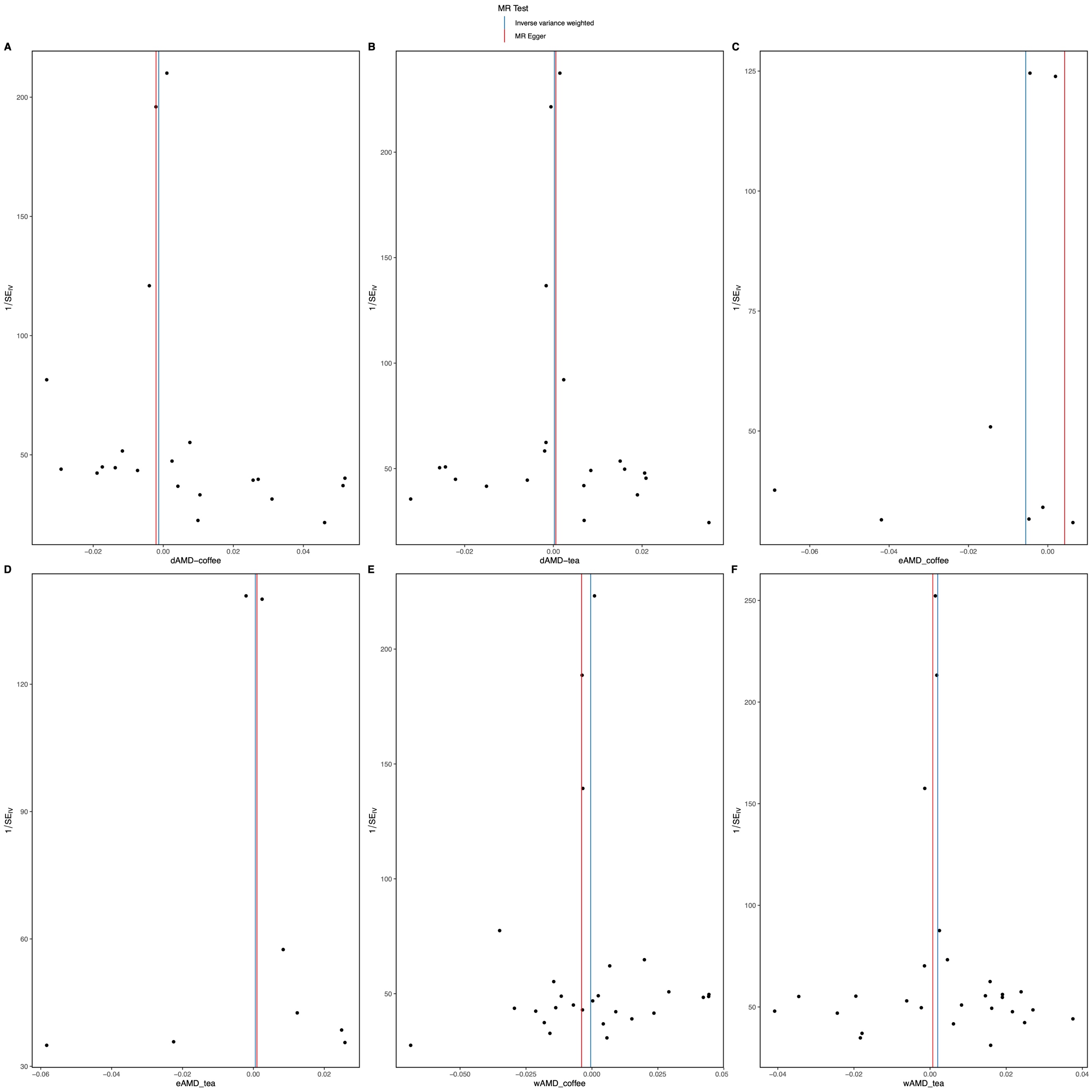


*Notes:* Panels A–B: Dry AMD (including geographic atrophy) on coffee consumption (A) and tea consumption (B). Panels C–D: Early AMD on coffee consumption (C) and tea consumption (D). Panels E–F: Wet AMD on coffee consumption (E) and tea consumption (F).

**Figure S11. Funnel Plot for Mendelian Randomization of Immune Cell Types and Dry AMD (Including Geographic Atrophy).**


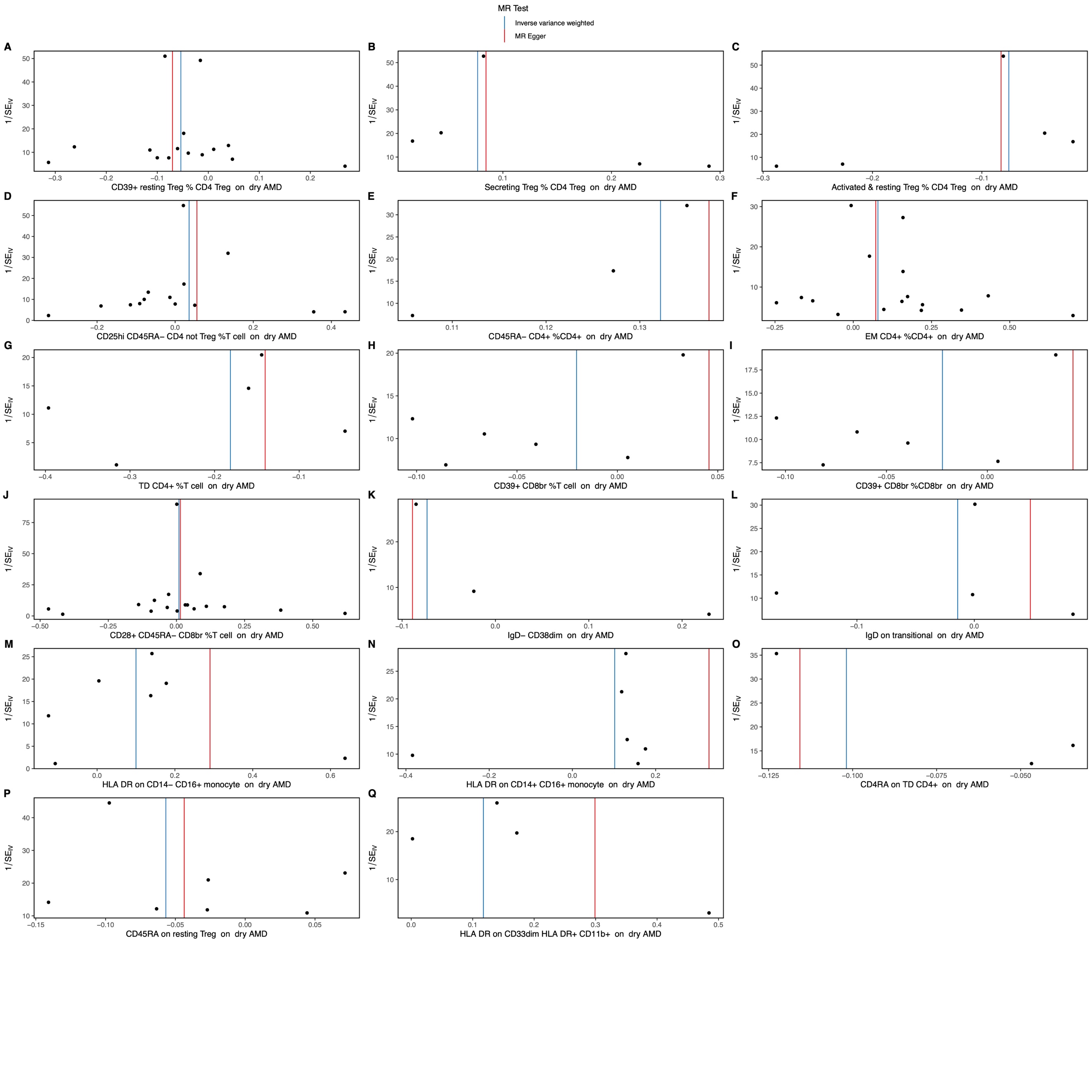


*Notes:* Panels A–Q: Funnel plots for 17 immune cell types on dry AMD (including geographic atrophy). A.CD39+ resting Treg % CD4 Treg; B. Secreting Treg % CD4 Treg; C. Activated & resting Treg % CD4 Treg; D. CD25 hi CD45RA- CD4 not Treg % T cell; E. CD 45 RA- CD4+ % CD4+; F. EM CD4+ % CD4+; G. TD CD4+ % T cell; H. CD39+ CD8br % T cell; I. CD39+ CD8br % CD8 br; J. CD28+ CD45RA-CD8br % T cell; K. IgD- CD38 dim; L. IgD on transitional; M. HLA DR on CD14- CD16+ monocyte; N. HLA DR on CD14+ CD16+ monocyte; O. CD4RA on TD CD4+; P. CD45RA on resting Treg; Q. HLA DR on CD33 dim HLA DR+ CD11b+.

**Figure S12. Funnel Plot for Mendelian Randomization of Tea Consumption and Immune Cell Types.**

**
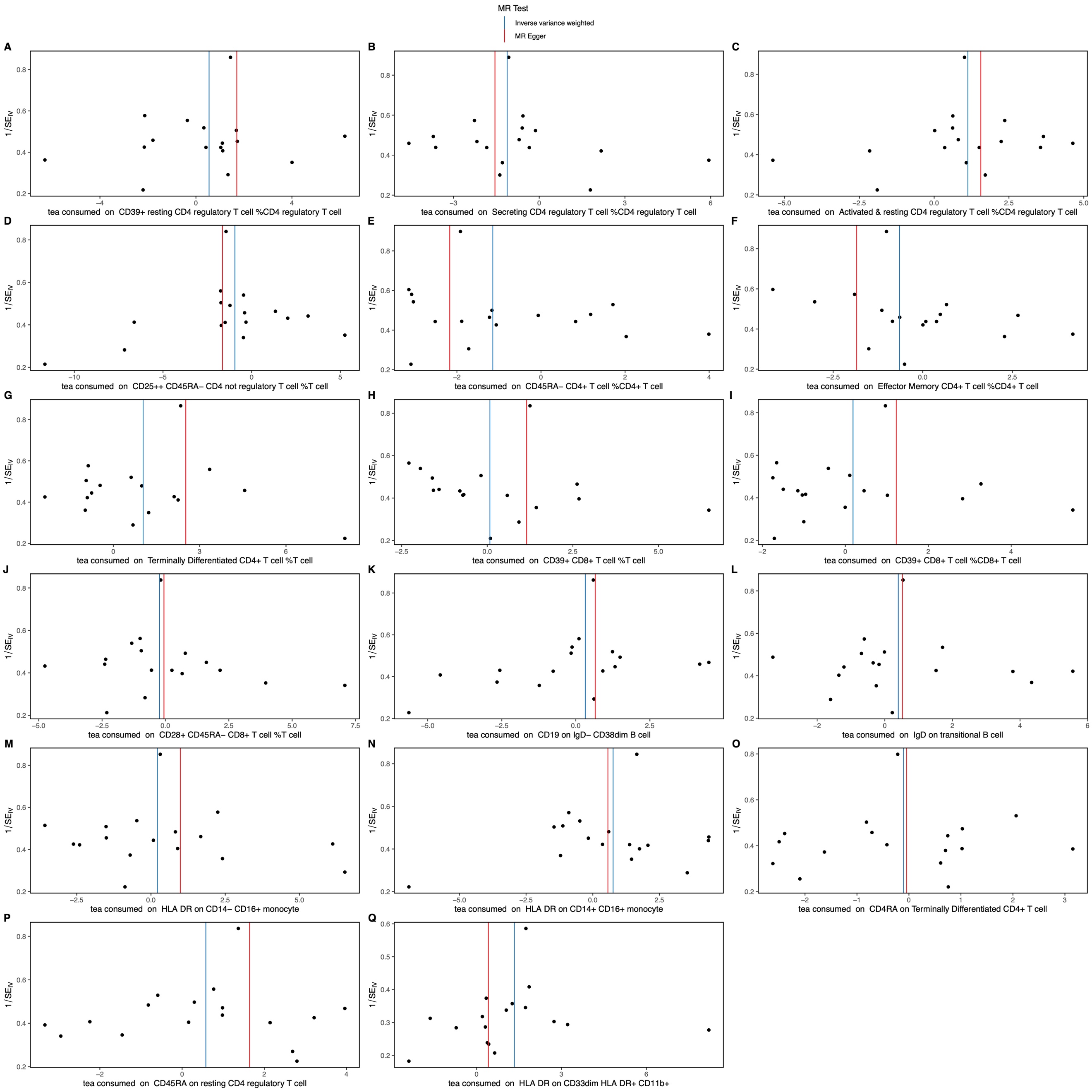
**

*Notes:* Panels A–Q: Funnel plots for tea consumption on 17 immune cell types. A.CD39+ resting Treg % CD4 Treg; B. Secreting Treg % CD4 Treg; C. Activated & resting Treg % CD4 Treg; D. CD25 hi CD45RA- CD4 not Treg % T cell; E. CD 45 RA- CD4+ % CD4+; F. EM CD4+ % CD4+; G. TD CD4+ % T cell; H. CD39+ CD8br % T cell; I. CD39+ CD8br % CD8 br; J. CD28+ CD45RA-CD8br % T cell; K. IgD- CD38 dim; L. IgD on transitional; M. HLA DR on CD14- CD16+ monocyte; N. HLA DR on CD14+ CD16+ monocyte; O. CD4RA on TD CD4+; P. CD45RA on resting Treg; Q. HLA DR on CD33 dim HLA DR+ CD11b+.

**STROBE-MR checklist of recommended items to address in reports of Mendelian randomization studies**

| **Item No.** | **Section** | **Checklist item** | **Page No.** | **Relevant text from manuscript** |
| --- | --- | --- | --- | --- |
| 1 | **TITLE and ABSTRACT** | Indicate Mendelian randomization (MR) as the study’s design in the title and/or the abstract if that is a main purpose of the study | 1, 2 | Caffeine Intake and Late Dry Age-Related Macular Degeneration: Tea's Protective Role—Insights from NHANES and Mendelian Randomization  Methods: Two-sample Mendelian randomization (MR) using GWAS summary statistics was employed to evaluate causal effects of tea and coffee consumption on AMD subtypes. Furthermore, a two-step MR approach was utilized to identify potential immune-mediated pathways. |
|  | **INTRODUCTION** |  |  |  |
| 2 | **Background** | Explain the scientific background and rationale for the reported study. What is the exposure? Is a potential causal relationship between exposure and outcome plausible? Justify why MR is a helpful method to address the study question | 3, 4, 5 | Age-related macular degeneration (AMD) is a leading cause of irreversible vision loss in older adults worldwide [1]. With the rapid aging of the population, its late-stage manifestations—neovascular (wet) AMD and geographic atrophy (GA, late-stage dry AMD) [2]—pose a substantial and growing economic and societal burden [3-5]. Although anti–vascular endothelial growth factor (anti-VEGF) therapy has revolutionized the prognosis of wet AMD in many patients, treatment responses vary considerably across individuals. More critically, effective therapeutic options for late-stage dry AMD remain largely unavailable in many regions [6], highlighting the urgent need for preventive strategies.  Chronic inflammation and immune dysregulation are widely recognized as central mechanisms in AMD pathogenesis [7,8]. Accumulating evidence indicates that pro-inflammatory T-cell subsets contribute to retinal degeneration and disease progression [9–11]. Caffeine, a widely consumed bioactive compound primarily derived from tea and coffee, has been reported to exert neuroprotective effects through anti-inflammatory and neuroimmunomodulatory pathways [12–19].  In recent years, Mendelian randomization (MR) has emerged as a powerful approach for strengthening causal inference in nutritional epidemiology and age-related eye diseases. Emerging MR studies have not only supported a protective role of water intake against age-related cataracts and diabetic retinopathy [23], but have also highlighted the contribution of gut microbiota—such as Lactobacillus—to visual and neurological health through the eye–brain–gut axis [24]. Importantly, MR investigations into caffeine and AMD have revealed a potential “source paradox” that was not apparent in traditional epidemiological studies. Specifically, a two-sample MR analysis of genetically predicted plasma caffeine levels demonstrated protective effects against cataracts and glaucoma but found no significant association with AMD [25]. In contrast, another MR study identified a causal association between instant coffee consumption and an increased risk of dry AMD [26]. This discrepancy—characterized by an overall lack of effect from plasma caffeine but potential harm from specific sources—suggests that caffeine cannot be evaluated as an isolated nutrient. Instead, its impact must be contextualized within its specific dietary carriers, such as tea and coffee [27,28]. |
| 3 | **Objectives** | State specific objectives clearly, including pre-specified causal hypotheses (if any). State that MR is a method that, under specific assumptions, intends to estimate causal effects | 5 | Second, to overcome the inherent limitations of observational analyses and to disentangle the proposed source-specific effects, we conducted two-sample MR analyses to evaluate the potential causal roles of tea and coffee—the two principal caffeine sources—on AMD subtypes. |
|  | **METHODS** |  |  |  |
| 4 | **Study design and data sources** | Present key elements of the study design early in the article. Consider including a table listing sources of data for all phases of the study. For each data source contributing to the analysis, describe the following: | 12 | Detailed data sources are listed in Table S1. |
|  | a) | Setting: Describe the study design and the underlying population, if possible. Describe the setting, locations, and relevant dates, including periods of recruitment, exposure, follow-up, and data collection, when available. | 11,12 | Summary-level GWAS data used in this study were obtained from publicly available datasets based on European cohorts, with all participants of European descent. Data on coffee and tea consumption—treated as binary variables based on the question "Did you drink coffee/tea yesterday?"—were sourced from the UK Biobank via the MRC IEU GWAS database (https://gwas.mrcieu.ac.uk/, accessed November 30, 2024) [34]. Early AMD outcomes were derived from summary statistics provided by the International AMD Genomics Consortium (IAMDGC), which included data from 11 cohorts (n = 105,248; 14,034 cases and 91,214 controls) [35]. Data for dry and wet AMD were obtained from the FinnGen research project (https://r11.finngen.fi/, accessed November 30, 2024), which categorizes age-related central vision loss due to retinal degeneration into wet AMD and dry AMD, including geographic atrophy (GA).  The mediator—immune cell type—was derived from a high-density genotyping array consisting of approximately 22 million single-nucleotide polymorphisms (SNPs), based on Sardinian sequences, as reported in the GWAS Catalog [36]. In MR analysis, sample overlap between exposure and outcome datasets can introduce bias and increase the risk of type I error. In our study, some early AMD outcome data from the UK Biobank partially overlapped with the exposure data on caffeinated beverage consumption. To account for this, we estimated the degree of bias and the probability of type I error using a publicly available tool (https://sb452.shinyapps.io/overlap/, accessed November 30, 2024) [37]. The FinnGen study, which aggregates data from nine Finnish biobank GWASs, and the immune cell type GWAS, conducted in participants from Sardinia (Mediterranean Italy), are genetically and geographically distinct from the UK Biobank cohort. Therefore, sample overlap across other exposures, mediators, and outcomes is likely minimal, reducing the risk of bias in our MR analyses. Detailed data sources are listed in Table S1. All GWAS summary-level data were derived from publicly available resources with prior ethical approval and written informed consent from all participants. |
|  | b) | Participants: Give the eligibility criteria, and the sources and methods of selection of participants. Report the sample size, and whether any power or sample size calculations were carried out prior to the main analysis | 11, 12 | Summary-level GWAS data used in this study were obtained from publicly available datasets based on European cohorts, with all participants of European descent. Data on coffee and tea consumption—treated as binary variables based on the question "Did you drink coffee/tea yesterday?"—were sourced from the UK Biobank via the MRC IEU GWAS database (https://gwas.mrcieu.ac.uk/, accessed November 30, 2024) [34]. Early AMD outcomes were derived from summary statistics provided by the International AMD Genomics Consortium (IAMDGC), which included data from 11 cohorts (n = 105,248; 14,034 cases and 91,214 controls) [35]. Data for dry and wet AMD were obtained from the FinnGen research project (https://r11.finngen.fi/, accessed November 30, 2024), which categorizes age-related central vision loss due to retinal degeneration into wet AMD and dry AMD, including geographic atrophy (GA).  The mediator—immune cell type—was derived from a high-density genotyping array consisting of approximately 22 million single-nucleotide polymorphisms (SNPs), based on Sardinian sequences, as reported in the GWAS Catalog [36]. In MR analysis, sample overlap between exposure and outcome datasets can introduce bias and increase the risk of type I error. In our study, some early AMD outcome data from the UK Biobank partially overlapped with the exposure data on caffeinated beverage consumption. To account for this, we estimated the degree of bias and the probability of type I error using a publicly available tool (https://sb452.shinyapps.io/overlap/, accessed November 30, 2024) [37]. The FinnGen study, which aggregates data from nine Finnish biobank GWASs, and the immune cell type GWAS, conducted in participants from Sardinia (Mediterranean Italy), are genetically and geographically distinct from the UK Biobank cohort. Therefore, sample overlap across other exposures, mediators, and outcomes is likely minimal, reducing the risk of bias in our MR analyses. Detailed data sources are listed in Table S1. All GWAS summary-level data were derived from publicly available resources with prior ethical approval and written informed consent from all participants. |
|  | c) | Describe measurement, quality control and selection of genetic variants | 12, 13 | To meet the three core assumptions of MR, we applied the following criteria for selecting SNPs as instrumental variables (IVs): (1) Relevance: SNPs were initially selected based on genome-wide significance (P < 5 × 10⁻⁸). Due to the limited number of SNPs identified for coffee and tea consumption, this threshold was relaxed to P < 5 × 10⁻⁶ to ensure an adequate number of instruments for MR analyses [38,39]. For the 17 immune cell types previously reported by Wei et al., we used a stricter threshold of P < 1 × 10⁻⁸, consistent with their methodology; if no SNPs met this criterion, we applied a relaxed threshold of P < 5 × 10⁻⁶. (2) Independence: Linkage disequilibrium (LD) between SNPs was addressed using a 10,000 kb window and an r² threshold of <0.001 to ensure independence. (3) Instrument strength: The strength of each IV was assessed using the F-statistic, calculated as F = R²(n − k − 1) / [k(1 − R²)], where R² = 2 × MAF × (1 − MAF) × β²; MAF is the minor allele frequency, n is the sample size, and k is the number of IVs. All selected SNPs had F-statistics >10, indicating sufficient instrument strength and minimizing the risk of weak instrument bias. (4) Harmonization and confounder exclusion: SNP effect alleles for the outcome were harmonized with those for the exposure based on allele letters and frequencies, and palindromic SNPs were excluded to avoid ambiguity. To ensure selected SNPs were not associated with potential confounders, we used the LDtrait Tool (https://ldlink.nih.gov/?tab=ldtrait, accessed November 30, 2024), with an r² threshold of 0.8. |
|  | d) | For each exposure, outcome, and other relevant variables, describe methods of assessment and diagnostic criteria for diseases | 11 | Summary-level GWAS data used in this study were obtained from publicly available datasets based on European cohorts, with all participants of European descent. Data on coffee and tea consumption—treated as binary variables based on the question “Did you drink coffee/tea yesterday?”—were sourced from the UK Biobank via the MRC IEU GWAS database (<https://gwas>.mrcieu.ac.uk/, accessed November 30, 2024) [34]. Early AMD outcomes were derived from summary statistics provided by the International AMD Genomics Consortium (IAMDGC), which included data from 11 cohorts (n = 105,248; 14,034 cases and 91,214 controls) [35]. Data for dry and wet AMD were obtained from the FinnGen research project (<https://r>11.finngen.fi/, accessed November 30, 2024), which categorizes age-related central vision loss due to retinal degeneration into wet AMD and dry AMD, including geographic atrophy (GA).  The mediator—immune cell type—was derived from a high-density genotyping array consisting of approximately 22 million single-nucleotide polymorphisms (SNPs), based on Sardinian sequences, as reported in the GWAS Catalog [36]. |
|  | e) | Provide details of ethics committee approval and participant informed consent, if relevant | 12 | All GWAS summary-level data were derived from publicly available resources with prior ethical approval and written informed consent from all participants. |
| 5 | **Assumptions** | Explicitly state the three core IV assumptions for the main analysis (relevance, independence and exclusion restriction) as well assumptions for any additional or sensitivity analysis | 12, 13, 14 | To meet the three core assumptions of MR, we applied the following criteria for selecting SNPs as instrumental variables (IVs): (1) Relevance: SNPs were initially selected based on genome-wide significance (P < 5 × 10⁻⁸). Due to the limited number of SNPs identified for coffee and tea consumption, this threshold was relaxed to P < 5 × 10⁻⁶ to ensure an adequate number of instruments for MR analyses [38,39]. For the 17 immune cell types previously reported by Wei et al., we used a stricter threshold of P < 1 × 10⁻⁸, consistent with their methodology; if no SNPs met this criterion, we applied a relaxed threshold of P < 5 × 10⁻⁶. (2) Independence: Linkage disequilibrium (LD) between SNPs was addressed using a 10,000 kb window and an r² threshold of <0.001 to ensure independence. (3) Instrument strength: The strength of each IV was assessed using the F-statistic, calculated as F = R²(n – k – 1) / [k(1 – R²)], where R² = 2 × MAF × (1 – MAF) × β²; MAF is the minor allele frequency, n is the sample size, and k is the number of Ivs. All selected SNPs had F-statistics >10, indicating sufficient instrument strength and minimizing the risk of weak instrument bias. (4) Harmonization and confounder exclusion: SNP effect alleles for the outcome were harmonized with those for the exposure based on allele letters and frequencies, and palindromic SNPs were excluded to avoid ambiguity. To ensure selected SNPs were not associated with potential confounders, we used the Ldtrait Tool (<https://ldlink>.nih.gov/?tab=ldtrait, accessed November 30, 2024), with an r² threshold of 0.8.  Sensitivity analysis is crucial to ensure the reliability of MR results. Heterogeneity among instrumental variables was assessed using Cochran’s Q statistic; with P > 0.05 indicating low heterogeneity. In the presence of heterogeneity, a random-effects inverse variance-weighted (IVW) model was applied. Horizontal pleiotropy was evaluated using the intercept from MR-Egger regression. We assessed the validity of the no measurement error (NOME) assumption in MR-Egger analyses using the I²GX statistic, which quantifies the impact of uncertainty in SNP–exposure associations on causal estimates. Additionally, leave-one-out (LOO) analysis was performed to identify and account for influential outliers. Funnel plots were generated to visually inspect the presence of directional pleiotropy. Detailed sensitivity analysis results—including the number of SNPs, F-statistic ranges, Cochran’s Q statistics, MR-Egger intercepts, I²GX values, LOO plots, and funnel plots—are provided in the Supplementary Materials. |
| 6 | **Statistical methods: main analysis** | Describe statistical methods and statistics used |  |  |
|  | a) | Describe how quantitative variables were handled in the analyses (i.e., scale, units, model) | 11 | Summary-level GWAS data used in this study were obtained from publicly available datasets based on European cohorts, with all participants of European descent. Data on coffee and tea consumption—treated as binary variables based on the question "Did you drink coffee/tea yesterday?"—were sourced from the UK Biobank via the MRC IEU GWAS database (https://gwas.mrcieu.ac.uk/, accessed November 30, 2024) [34]. Early AMD outcomes were derived from summary statistics provided by the International AMD Genomics Consortium (IAMDGC), which included data from 11 cohorts (n = 105,248; 14,034 cases and 91,214 controls) [35]. Data for dry and wet AMD were obtained from the FinnGen research project (https://r11.finngen.fi/, accessed November 30, 2024), which categorizes age-related central vision loss due to retinal degeneration into wet AMD and dry AMD, including geographic atrophy (GA).  The mediator—immune cell type—was derived from a high-density genotyping array consisting of approximately 22 million single-nucleotide polymorphisms (SNPs), based on Sardinian sequences, as reported in the GWAS Catalog [36]. |
|  | b) | Describe how genetic variants were handled in the analyses and, if applicable, how their weights were selected | 12, 13 | To meet the three core assumptions of MR, we applied the following criteria for selecting SNPs as instrumental variables (IVs): (1) Relevance: SNPs were initially selected based on genome-wide significance (P < 5 × 10⁻⁸). Due to the limited number of SNPs identified for coffee and tea consumption, this threshold was relaxed to P < 5 × 10⁻⁶ to ensure an adequate number of instruments for MR analyses [38,39]. For the 17 immune cell types previously reported by Wei et al., we used a stricter threshold of P < 1 × 10⁻⁸, consistent with their methodology; if no SNPs met this criterion, we applied a relaxed threshold of P < 5 × 10⁻⁶. (2) Independence: Linkage disequilibrium (LD) between SNPs was addressed using a 10,000 kb window and an r² threshold of <0.001 to ensure independence. (3) Instrument strength: The strength of each IV was assessed using the F-statistic, calculated as F = R²(n − k − 1) / [k(1 − R²)], where R² = 2 × MAF × (1 − MAF) × β²; MAF is the minor allele frequency, n is the sample size, and k is the number of IVs. All selected SNPs had F-statistics >10, indicating sufficient instrument strength and minimizing the risk of weak instrument bias. (4) Harmonization and confounder exclusion: SNP effect alleles for the outcome were harmonized with those for the exposure based on allele letters and frequencies, and palindromic SNPs were excluded to avoid ambiguity. To ensure selected SNPs were not associated with potential confounders, we used the LDtrait Tool (https://ldlink.nih.gov/?tab=ldtrait, accessed November 30, 2024), with an r² threshold of 0.8. |
|  | c) | Describe the MR estimator (e.g. two-stage least squares, Wald ratio) and related statistics. Detail the included covariates and, in case of two-sample MR, whether the same covariate set was used for adjustment in the two samples | 13, 15 | To ensure selected SNPs were not associated with potential confounders, we used the LDtrait Tool (https://ldlink.nih.gov/?tab=ldtrait, accessed November 30, 2024), with an r² threshold of 0.8.  The Inverse-Variance Weighted (IVW) method with random effects served as the primary analysis due to its high statistical power, supplemented by four default methods in the TwoSampleMR package: MR-Egger, weighted median, weighted mode, and simple mode. |
|  | d) | Explain how missing data were addressed | 13, 14 | The strength of each IV was assessed by calculating F-statistics, where R²=2×MAF×(1−MAF)×β², and F=R²(n−k−1)/k(1−R²), with MAF as the minor allele frequency, n as sample size, and k as the number of IVs, ensuring all F-statistics exceeded 10 to exclude weak IV bias.  Effect alleles of outcome-associated SNPs were aligned with exposure-associated SNPs based on allele letters and frequencies, removing palindromic SNPs.  The LDtrait Tool database (https://ldlink.nih.gov/?tab=ldtrait) with an R² threshold of 0.8 was used to confirm that SNP loci were not associated with other confounders. |
|  | e) | If applicable, indicate how multiple testing was addressed |  | NA |
| 7 | **Assessment of assumptions** | Describe any methods or prior knowledge used to assess the assumptions or justify their validity | 15 | The Inverse-Variance Weighted (IVW) method with random effects served as the primary analysis due to its high statistical power, supplemented by four default methods in the TwoSampleMR package: MR-Egger, weighted median, weighted mode, and simple mode.  Statistical significance was determined when the IVW P-value was <0.05 and all five methods showed consistent effect directions. A causal effect between exposure and outcome was inferred when forward MR was significant and reverse MR showed no effect. |
| 8 | **Sensitivity analyses and additional analyses** | Describe any sensitivity analyses or additional analyses performed (e.g. comparison of effect estimates from different approaches, independent replication, bias analytic techniques, validation of instruments, simulations) | 13, 14 | Sensitivity analysis is crucial to ensure the reliability of MR results. Heterogeneity among instrumental variables was assessed using Cochran’s Q statistic; with P > 0.05 indicating low heterogeneity. In the presence of heterogeneity, a random-effects inverse variance-weighted (IVW) model was applied. Horizontal pleiotropy was evaluated using the intercept from MR-Egger regression. We assessed the validity of the no measurement error (NOME) assumption in MR-Egger analyses using the I²GX statistic, which quantifies the impact of uncertainty in SNP–exposure associations on causal estimates. Additionally, leave-one-out (LOO) analysis was performed to identify and account for influential outliers. Funnel plots were generated to visually inspect the presence of directional pleiotropy. Detailed sensitivity analysis results—including the number of SNPs, F-statistic ranges, Cochran’s Q statistics, MR-Egger intercepts, I²GX values, LOO plots, and funnel plots—are provided in the Supplementary Materials. |
| 9 | **Software and pre-registration** |  |  |  |
|  | a) | Name statistical software and package(s), including version and settings used | 14, 15 | All statistical analyses were performed using R software (version 4.4.1).  All statistical analyses were conducted using the R package "TwoSampleMR" (version 0.6.8). Sensitivity analyses included MR-Egger, weighted median, weighted mode, and simple mode. |
|  | b) | State whether the study protocol and details were pre-registered (as well as when and where) |  | NA |
|  | **RESULTS** |  |  |  |
| 10 | **Descriptive data** |  |  |  |
|  | a) | Report the numbers of individuals at each stage of included studies and reasons for exclusion. Consider use of a flow diagram | 26 | Shown in Figure 3A.  Supplemental Table 4 providing SNP data analyzed by MR. |
|  | b) | Report summary statistics for phenotypic exposure(s), outcome(s), and other relevant variables (e.g. means, SDs, proportions) | 24, 25 | As shown in Figure 3A, the IVW model indicated no significant causal association between coffee or tea consumption and early AMD (coffee consumption, OR = 1.12, 95% CI: [0.61-2.04], P = 0.7244; tea consumption, OR = 1.25, 95% CI: [0.58-2.72], P = 0.5724). Regarding more advanced AMD subtypes, coffee consumption did not significantly associate with either wet AMD or dry AMD, including geographic atrophy (wet AMD, OR = 1.38, 95% CI: [0.60-3.16], P = 0.4489; dry AMD, including geographic atrophy, OR = 1.34, 95% CI: [0.65-2.77], P = 0.4237). Tea consumption, however, showed a statistically significant inverse association with the risk of dry AMD, including geographic atrophy (OR=0.44, 95% CI: [0.20-0.97], P=0.0418). Although alternative MR methods did not achieve statistical significance, scatter plots revealed consistent effect directions with the IVW results (Figure 3B–G), supporting the IVW-based inference [41]. Reverse MR analyses revealed no evidence of reverse causality between AMD subtypes and coffee or tea consumption (Figure S1). |
|  | c) | If the data sources include meta-analyses of previous studies, provide the assessments of heterogeneity across these studies |  | NA |
|  | d) | For two-sample MR:  i.  Provide justification of the similarity of the genetic variant-exposure associations between the exposure and outcome samples  ii.  Provide information on the number of individuals who overlap between the exposure and outcome studies | 11, 12, 24 | In our study, some early AMD outcome data from the UK Biobank partially overlapped with the exposure data on caffeinated beverage consumption. To account for this, we estimated the degree of bias and the probability of type I error using a publicly available tool (https://sb452.shinyapps.io/overlap/, accessed November 30, 2024) [37]. The FinnGen study, which aggregates data from nine Finnish biobank GWASs, and the immune cell type GWAS, conducted in participants from Sardinia (Mediterranean Italy), are genetically and geographically distinct from the UK Biobank cohort. Therefore, sample overlap across other exposures, mediators, and outcomes is likely minimal, reducing the risk of bias in our MR analyses.  The evidence became even less suggestive of a causal link after adjusting for bias and type I error due to sample overlap (Table S3). |
| 11 | **Main results** |  |  |  |
|  | a) | Report the associations between genetic variant and exposure, and between genetic variant and outcome, preferably on an interpretable scale | 24, 25 | As shown in Figure 3A, the IVW model indicated no significant causal association between coffee or tea consumption and early AMD (coffee consumption, OR = 1.12, 95% CI: [0.61-2.04], P = 0.7244; tea consumption, OR = 1.25, 95% CI: [0.58-2.72], P = 0.5724), and the evidence became even less suggestive of a causal link after adjusting for bias and type I error due to sample overlap (Table S3). These null findings are consistent with our cross-sectional analysis, which also showed lack of association between caffeine intake and early-stage AMD.  Regarding more advanced AMD subtypes, coffee consumption did not significantly associate with either wet AMD or dry AMD, including geographic atrophy (wet AMD, OR = 1.38, 95% CI: [0.60-3.16], P = 0.4489; dry AMD, including geographic atrophy, OR = 1.34, 95% CI: [0.65-2.77], P = 0.4237). Tea consumption, however, showed a statistically significant inverse association with the risk of dry AMD, including geographic atrophy (OR=0.44, 95% CI: [0.20-0.97], P=0.0418). Although alternative MR methods did not achieve statistical significance, scatter plots revealed consistent effect directions with the IVW results (Figure 3B–G), supporting the IVW-based inference [41]. Reverse MR analyses revealed no evidence of reverse causality between AMD subtypes and coffee or tea consumption (Figure S1). |
|  | b) | Report MR estimates of the relationship between exposure and outcome, and the measures of uncertainty from the MR analysis, on an interpretable scale, such as odds ratio or relative risk per SD difference | 24, 25 | As shown in Figure 3A, the IVW model indicated no significant causal association between coffee or tea consumption and early AMD (coffee consumption, OR = 1.12, 95% CI: [0.61-2.04], P = 0.7244; tea consumption, OR = 1.25, 95% CI: [0.58-2.72], P = 0.5724), and the evidence became even less suggestive of a causal link after adjusting for bias and type I error due to sample overlap (Table S3). These null findings are consistent with our cross-sectional analysis, which also showed lack of association between caffeine intake and early-stage AMD.  Regarding more advanced AMD subtypes, coffee consumption did not significantly associate with either wet AMD or dry AMD, including geographic atrophy (wet AMD, OR = 1.38, 95% CI: [0.60-3.16], P = 0.4489; dry AMD, including geographic atrophy, OR = 1.34, 95% CI: [0.65-2.77], P = 0.4237). Tea consumption, however, showed a statistically significant inverse association with the risk of dry AMD, including geographic atrophy (OR=0.44, 95% CI: [0.20-0.97], P=0.0418). Although alternative MR methods did not achieve statistical significance, scatter plots revealed consistent effect directions with the IVW results (Figure 3B–G), supporting the IVW-based inference [41]. Reverse MR analyses revealed no evidence of reverse causality between AMD subtypes and coffee or tea consumption (Figure S1). |
|  | c) | If relevant, consider translating estimates of relative risk into absolute risk for a meaningful time period |  | NA |
|  | d) | Consider plots to visualize results (e.g. forest plot, scatterplot of associations between genetic variants and outcome versus between genetic variants and exposure) | 24, 26, 28 | As shown in Figure 3A  Figure 3. Forest and scatter plots depicting the causal association between coffee/tea consumption and AMD subtypes.  Figure 4. Forest plot of immune cell types linking tea consumption and dry AMD.  Scatter plots illustrating these associations are shown in Figure S4. |
| 12 | **Assessment of assumptions** |  |  |  |
|  | a) | Report the assessment of the validity of the assumptions | 12, 15, 24, 25, 30 | Instrument strength: The strength of each IV was assessed using the F-statistic, calculated as F = R²(n – k – 1) / [k(1 – R²)], where R² = 2 × MAF × (1 – MAF) × β²; MAF is the minor allele frequency, n is the sample size, and k is the number of Ivs. All selected SNPs had F-statistics >10, indicating sufficient instrument strength and minimizing the risk of weak instrument bias.  Statistical significance was defined as IVW P < 0.05 with concordant effect directions across all methods. Causal inference required significant forward MR and null reverse MR results.  As shown in Figure 3A, the IVW model indicated no significant causal association between coffee or tea consumption and early AMD (coffee consumption, OR = 1.12, 95% CI: [0.61-2.04], P = 0.7244; tea consumption, OR = 1.25, 95% CI: [0.58-2.72], P = 0.5724), and the evidence became even less suggestive of a causal link after adjusting for bias and type I error due to sample overlap (Table S3). These null findings are consistent with our cross-sectional analysis, which also showed lack of association between caffeine intake and early-stage AMD.  Regarding more advanced AMD subtypes, coffee consumption did not significantly associate with either wet AMD or dry AMD, including geographic atrophy (wet AMD, OR = 1.38, 95% CI: [0.60-3.16], P = 0.4489; dry AMD, including geographic atrophy, OR = 1.34, 95% CI: [0.65-2.77], P = 0.4237). Tea consumption, however, showed a statistically significant inverse association with the risk of dry AMD, including geographic atrophy (OR=0.44, 95% CI: [0.20-0.97], P=0.0418). Although alternative MR methods did not achieve statistical significance, scatter plots revealed consistent effect directions with the IVW results (Figure 3B–G), supporting the IVW-based inference [41]. Reverse MR analyses revealed no evidence of reverse causality between AMD subtypes and coffee or tea consumption (Figure S1).  To ensure the robustness of the MR findings, we performed a comprehensive suite of sensitivity analyses. For the key MR conclusions reported in this study—including the causal effects of tea consumption on dry AMD (including geographic atrophy) and its mediation via immune cell types—no evidence of horizontal pleiotropy (P > 0.05 for MR-Egger intercept) or significant heterogeneity (P > 0.05 for Cochran’s Q) was observed, as detailed in Table S5–8. Although modest heterogeneity was detected for the association between Secreting Treg % CD4+ Treg and dry AMD (including geographic atrophy), the causal estimate remained reliable owing to the application of a random-effects model. In addition, the I²GX statistic was calculated to evaluate the validity of the no measurement error (NOME) assumption in the MR-Egger analyses. The I²GX values for tea (0.829), coffee (0.758), and the primary immune cell instrumental variables all exceeded the recommended threshold (>0.60), indicating a low risk of bias due to weak instrument measurement error (Tables S5–S8). Finally, leave-one-out analyses (Figure S5–8) and funnel plot (Figure S9–12) inspections further supported the robustness of the MR results, with no single SNP exerting a disproportionate influence on the overall causal estimates. |
|  | b) | Report any additional statistics (e.g., assessments of heterogeneity across genetic variants, such as *I^2^*, Q statistic or E-value) | 30 | In addition, the I²GX statistic was calculated to evaluate the validity of the no measurement error (NOME) assumption in the MR-Egger analyses. The I²GX values for tea (0.829), coffee (0.758), and the primary immune cell instrumental variables all exceeded the recommended threshold (>0.60), indicating a low risk of bias due to weak instrument measurement error (Tables S5–S8). |
| 13 | **Sensitivity analyses and additional analyses** |  |  |  |
|  | a) | Report any sensitivity analyses to assess the robustness of the main results to violations of the assumptions | 30 | To ensure the robustness of the MR findings, we performed a comprehensive suite of sensitivity analyses. For the key MR conclusions reported in this study—including the causal effects of tea consumption on dry AMD (including geographic atrophy) and its mediation via immune cell types—no evidence of horizontal pleiotropy (P > 0.05 for MR-Egger intercept) or significant heterogeneity (P > 0.05 for Cochran’s Q) was observed, as detailed in Table S5–8. Although modest heterogeneity was detected for the association between Secreting Treg % CD4+ Treg and dry AMD (including geographic atrophy), the causal estimate remained reliable owing to the application of a random-effects model. In addition, the I²GX statistic was calculated to evaluate the validity of the no measurement error (NOME) assumption in the MR-Egger analyses. The I²GX values for tea (0.829), coffee (0.758), and the primary immune cell instrumental variables all exceeded the recommended threshold (>0.60), indicating a low risk of bias due to weak instrument measurement error (Tables S5–S8). Finally, leave-one-out analyses (Figure S5–8) and funnel plot (Figure S9–12) inspections further supported the robustness of the MR results, with no single SNP exerting a disproportionate influence on the overall causal estimates. |
|  | b) | Report results from other sensitivity analyses or additional analyses | 30 | To ensure the robustness of the MR findings, we performed a comprehensive suite of sensitivity analyses. For the key MR conclusions reported in this study—including the causal effects of tea consumption on dry AMD (including geographic atrophy) and its mediation via immune cell types—no evidence of horizontal pleiotropy (P > 0.05 for MR-Egger intercept) or significant heterogeneity (P > 0.05 for Cochran’s Q) was observed, as detailed in Table S5–8. Although modest heterogeneity was detected for the association between Secreting Treg % CD4+ Treg and dry AMD (including geographic atrophy), the causal estimate remained reliable owing to the application of a random-effects model. In addition, the I²GX statistic was calculated to evaluate the validity of the no measurement error (NOME) assumption in the MR-Egger analyses. The I²GX values for tea (0.829), coffee (0.758), and the primary immune cell instrumental variables all exceeded the recommended threshold (>0.60), indicating a low risk of bias due to weak instrument measurement error (Tables S5–S8). Finally, leave-one-out analyses (Figure S5–8) and funnel plot (Figure S9–12) inspections further supported the robustness of the MR results, with no single SNP exerting a disproportionate influence on the overall causal estimates. |
|  | c) | Report any assessment of direction of causal relationship (e.g., bidirectional MR) | 25 | Reverse MR analyses revealed no evidence of reverse causality between AMD subtypes and coffee or tea consumption (Figure S1). |
|  | d) | When relevant, report and compare with estimates from non-MR analyses | 25 | This aligns with our RCS findings, as tea typically delivers a moderate caffeine dose near the intake level associated with lowest AMD risk. |
|  | e) | Consider additional plots to visualize results (e.g., leave-one-out analyses) | 27, 30 | However, leave-one-out analysis identified a highly influential SNP for IgD− CD38dim (Supplemental Figure 7K); exclusion of this SNP rendered the association nonsignificant (P = 0.845, from an original P = 0.028). Therefore, 10 immune cell types demonstrated robust causal associations with dry AMD (including geographic atrophy), largely consistent with Wei et al.'s specific findings for dry AMD subtype.Finally, leave-one-out analyses (Figure S5–8) and funnel plot (Figure S9–12) inspections further supported the robustness of the MR results, with no single SNP exerting a disproportionate influence on the overall causal estimates. |
|  | **DISCUSSION** |  |  |  |
| 14 | **Key results** | Summarize key results with reference to study objectives | 31 | At the genetic level, MR analyses refined this observation by demonstrating that tea consumption—but not coffee consumption—was associated with a potential causal protective effect against dry AMD, including geographic atrophy. Subsequent two-step MR mediation analyses provided evidence that this association may be partially mediated through immune-related pathways including reductions in the proportions of secretory regulatory CD4+ T cells and CD45RA− CD4+ T cells. |
| 15 | **Limitations** | Discuss limitations of the study, taking into account the validity of the IV assumptions, other sources of potential bias, and imprecision. Discuss both direction and magnitude of any potential bias and any efforts to address them | 38, 39 | This study has several limitations that should be acknowledged. First, the cross-sectional design of the NHANES analysis precludes causal inference and is inherently susceptible to reverse causality (e.g., dietary modification following illness) as well as recall bias in dietary assessment. Although we adjusted for a wide range of potential confounders and incorporated Mendelian randomization (MR) as a complementary analytical approach, residual confounding cannot be fully excluded. Second, the relatively small number of late-stage AMD cases in NHANES (n = 47) raises the possibility of sparsity bias. To mitigate overfitting, we applied a simplified restricted cubic spline (RCS) model with the minimum number of knots and reported a suggested saturation range rather than a precise threshold. Nevertheless, these dose–response findings should be interpreted cautiously. Third, the outcome definitions differed between the two analytical frameworks: NHANES defined “late AMD” as a mixed outcome including both exudative AMD and geographic atrophy, whereas the MR analysis focused specifically on dry AMD (including geographic atrophy). Rather than viewing this as a contradiction, we interpret the results as complementary: the NHANES analysis identifies a population-level protective association with late-stage disease, while the MR analysis refines this signal toward a dry/atrophic phenotype. Fourth, due to the limited availability of genetic instruments for coffee and tea consumption, a relatively lenient SNP selection threshold (P < 5 × 10⁻⁶) was applied in the MR analysis. Although all selected instruments demonstrated adequate strength based on F-statistics, this approach may still affect the precision of causal estimates. Fifth, the mediation analysis is based on genetic inference and does not constitute direct mechanistic evidence. Functional studies are therefore required to validate whether the immune pathways identified—particularly those involving regulatory and memory T cell subsets—are directly modulated by tea-derived bioactive components in the context of AMD. Finally, NHANES dietary data do not allow isolation of the independent observational effects of non-caffeine components, such as tea polyphenols, and the genetic datasets used in the MR analyses were primarily derived from European-ancestry populations. Accordingly, the generalizability of these findings to other ethnic groups and dietary patterns warrants further investigation. |
| 16 | **Interpretation** |  |  |  |
|  | a) | Meaning: Give a cautious overall interpretation of results in the context of their limitations and in comparison with other studies | 33 | These findings are consistent with the broader shift in nutritional epidemiology from isolated nutrient–based analyses toward whole-food and source-specific exposure assessments [27,28]. Our results underscore the importance of considering caffeine carriers rather than caffeine intake alone. Accumulating evidence suggests that the health effects of caffeine vary by dietary source. For instance, caffeine derived from coffee has been more strongly associated with reduced risks of coronary artery disease and type 2 diabetes [54], whereas caffeine from tea has been linked to delayed coronary artery calcification [55] and shows a linear inverse association with Parkinson’s disease risk [14]. In this context, the differential effects of coffee and tea observed in the MR analyses may reflect a combination of dose–response characteristics, processing-related byproducts, and source-specific bioactive components. |
|  | b) | Mechanism: Discuss underlying biological mechanisms that could drive a potential causal relationship between the investigated exposure and the outcome, and whether the gene-environment equivalence assumption is reasonable. Use causal language carefully, clarifying that IV estimates may provide causal effects only under certain assumptions | 35, 36, 37, 38 | Mechanistic Insights into Tea’s Protective Effects on Dry AMD (Including Geographic Atrophy)  In the mediation MR analysis, tea consumption significantly influenced 3 of the 10 immune cell types that were causally associated with dry AMD. The observation that nearly one-third of the total causal effect could be statistically attributed to these immune mediators suggests a biologically plausible link between tea consumption and dry AMD. Although these mediation findings require confirmation through functional experiments and clinical studies, the identified immune cell changes are consistent with both the known immunomodulatory effects of tea-derived bioactive compounds and the established immunopathological features of AMD. Together, these results provide a preliminary and coherent mechanistic framework for interpreting the observed associations.  Among the immune cell subsets affected by tea consumption, secretory regulatory T cells (Tregs) and activated/resting Tregs represent functionally distinct and complementary regulatory T cell populations. Secreting Tregs typically exhibit pro-inflammatory characteristics, shifting toward a Th17-like function rather than the classical immunosuppressive phenotype. Previous studies have established that in the immunopathological context of AMD, Tregs play a critical role in maintaining retinal immune homeostasis and suppressing chronic inflammation. Activated/resting Tregs can attenuate damage to the RPE and choroid, maintaining photoreceptor integrity by suppressing local inflammation [71,72]. Conversely, secreting Tregs release TGF-β and IL-17, which accelerate RPE senescence and damage [49–51,73,74].  The MR-mediated pattern observed in this study—characterized by a reduction in secretory Tregs and a relative increase in activated/resting Tregs—is highly concordant with existing evidence on how tea polyphenols, particularly EGCG, regulate T cell plasticity. Mechanistically, EGCG has been shown to inhibit STAT3 signaling, a key molecular switch driving the transdifferentiation of Tregs toward a Th17-like, pro-inflammatory phenotype. Inhibition of STAT3 phosphorylation suppresses downstream expression of IL-17 and related inflammatory mediators [75]. In addition, EGCG can interfere with the mTOR–HIF-1α metabolic axis, thereby modulating the balance between Th17 and Treg differentiation [76]. At the epigenetic level, EGCG exhibits DNA methyltransferase (DNMT) inhibitory activity, which helps maintain hypomethylation of the Foxp3 promoter and stabilizes the immunosuppressive phenotype of Tregs, limiting pro-inflammatory differentiation of CD4⁺ T cells [77]. In contrast to tea polyphenols, caffeine appears to exert immunomodulatory effects primarily by modulating immune cell function rather than directly adjusting Treg abundance. Caffeine antagonizes adenosine A2A receptor signaling, thereby influencing the intensity of Treg-mediated immunosuppression and indirectly shaping the functional balance among T cell subsets [78]. Taken together, these mechanisms provide a plausible immunological explanation for the observed association between tea consumption, suppression of pro-inflammatory Treg phenotypes, and reduced risk of dry AMD.  In addition to regulatory T cell subsets, tea consumption was associated with changes in CD45RA⁻CD4⁺ T cells, which largely represent antigen-experienced memory T cells and are widely regarded as markers of immunosenescence. Elevated levels of CD45RA⁻CD4⁺ memory T cells have been consistently reported in patients with AMD and have been linked to disease progression [79–81]. Experimental evidence suggests that caffeine can inhibit the differentiation of naïve T cells into memory T cells by interfering with adenosine receptor–mediated signaling pathways [82]. Moreover, tea polyphenols have been shown to suppress excessive T cell activation and limit the accumulation of memory T cells under chronic inflammatory conditions [83]. Such coordinated regulation may reduce the infiltration of senescent T cells into retinal tissue and contribute to improved immune microenvironment homeostasis [7,84].  In summary, the immune cell alterations identified through mediation MR analysis are biologically consistent with both the immunopathology of AMD and the established immunomodulatory actions of tea polyphenols and caffeine. Nevertheless, the current evidence is derived primarily from genetic inference, observational epidemiology, and preclinical studies. Future investigations should directly evaluate the effects of tea-derived compounds on retinal T cell infiltration and function using AMD animal models and human retinal samples, as well as explore potential interaction effects between caffeine and catechins in modulating retinal immune responses. |
|  | c) | Clinical relevance: Discuss whether the results have clinical or public policy relevance, and to what extent they inform effect sizes of possible interventions | 40 | These findings suggest that caffeine content alone may be insufficient to explain the observed protective association and that the combined effects of caffeine with non-caffeine bioactive compounds present in tea may be more relevant. Moreover, mediation MR analyses identified specific immune cell subsets—particularly secretory regulatory CD4+ T cells and CD45RA⁻ CD4+ T cells—as potential mediators, providing a biologically plausible immunological context for the association between tea intake and reduced dry AMD risk. Although the precise effector components and molecular mechanisms remain to be elucidated, this study offers novel epidemiological and genetic evidence supporting the importance of dietary source–specific effects in AMD and may inform future research on targeted dietary prevention strategies. |
| 17 | **Generalizability** | Discuss the generalizability of the study results (a) to other populations, (b) across other exposure periods/timings, and (c) across other levels of exposure | 39 | the genetic datasets used in the MR analyses were primarily derived from European-ancestry populations. Accordingly, the generalizability of these findings to other ethnic groups and dietary patterns warrants further investigation. |
|  | **OTHER INFORMATION** |  |  |  |
| 18 | **Funding** | Describe sources of funding and the role of funders in the present study and, if applicable, sources of funding for the databases and original study or studies on which the present study is based | 43 | Funding: This work was supported by the Tianjin Key Medical Discipline ( Specialty ) Construction Project（TJYXZDXK- 037A）. |
| 19 | **Data and data sharing** | Provide the data used to perform all analyses or report where and how the data can be accessed, and reference these sources in the article. Provide the statistical code needed to reproduce the results in the article, or report whether the code is publicly accessible and if so, where | 42, 43 | Availability of data and materials  The data used in this study, including publicly available genome-wide association study (GWAS) summary statistics and NHANES datasets, are accessible online. Specific dataset names and access links are provided in the main text or Supplementary Materials. NHANES data are available at https://www.cdc.gov/nchs/nhanes/. GWAS summary statistics for tea and coffee consumption were obtained from the UK Biobank and can be accessed by searching the relevant traits at https://gwas.mrcieu.ac.uk/. GWAS data for dry and wet AMD were obtained from the FinnGen consortium at https://r11.finngen.fi/. GWAS data for early AMD were derived from the study by Winkler et al, DOI: 10.1186/s12920-020-00760-7. Summary statistics for immune cell phenotypes are available through the GWAS Catalog at https://www.ebi.ac.uk/gwas/ using the corresponding IDs. All datasets were accessed on November 30, 2024. |
| 20 | **Conflicts of Interest** | All authors should declare all potential conflicts of interest | 43 | Financial Disclosures: The authors have declared that no conflicts of interest exist. |

This checklist is copyrighted by the Equator Network under the Creative Commons Attribution 3.0 Unported (CC BY 3.0) license.
